# Fc Receptor-Like 6 (FCRL6) Discloses Progenitor B Cell Heterogeneity That Correlates With Pre-BCR Dependent and Independent Pathways of Natural Antibody Selection

**DOI:** 10.3389/fimmu.2020.00082

**Published:** 2020-02-14

**Authors:** Kazuhito Honjo, Woong-Jai Won, Rodney G. King, Lara Ianov, David K. Crossman, Juliet L. Easlick, Mikhail A. Shakhmatov, Mohamed Khass, Andre M. Vale, Robert P. Stephan, Ran Li, Randall S. Davis

**Affiliations:** ^1^Department of Medicine, University of Alabama at Birmingham, Birmingham, AL, United States; ^2^Department of Microbiology, University of Alabama at Birmingham, Birmingham, AL, United States; ^3^Civitan International Research Center, University of Alabama at Birmingham, Birmingham, AL, United States; ^4^Department of Genetics, University of Alabama at Birmingham, Birmingham, AL, United States; ^5^Genetic Engineering and Biotechnology Division, National Research Center, Cairo, Egypt; ^6^Program in Immunobiology, Carlos Chagas Filho Institute of Biophysics, Federal University of Rio de Janeiro (UFRJ), Rio de Janeiro, Brazil; ^7^Department of Biochemistry and Molecular Genetics, University of Alabama at Birmingham, Birmingham, AL, United States; ^8^Comprehensive Cancer Center, University of Alabama at Birmingham, Birmingham, AL, United States

**Keywords:** B-1 cells, innate-like B cells, natural antibodies, antibody repertoire, lymphocyte development, lymphocyte selection, autoimmunity, chronic lymphocytic leukemia

## Abstract

B-1a cells produce “natural” antibodies (Abs) to neutralize pathogens and clear neo self-antigens, but the fundamental selection mechanisms that shape their polyreactive repertoires are poorly understood. Here, we identified a B cell progenitor subset defined by Fc receptor-like 6 (FCRL6) expression, harboring innate-like defense, migration, and differentiation properties conducive for natural Ab generation. Compared to FCRL6^−^ pro B cells, the repressed mitotic, DNA damage repair, and signaling activity of FCRL6^+^ progenitors, yielded V_H_ repertoires with biased distal *Ighv* segment accessibility, constrained diversity, and hydrophobic and charged CDR-H3 sequences. Beyond nascent autoreactivity, V_H_11 productivity, which predominates phosphatidylcholine-specific B-1a B cell receptors (BCRs), was higher for FCRL6^+^ cells as was pre-BCR formation, which was required for Myc induction and V_H_11, but not V_H_12, B-1a development. Thus, FCRL6 revealed unexpected heterogeneity in the developmental origins, regulation, and selection of natural Abs at the pre-BCR checkpoint with implications for autoimmunity and lymphoproliferative disorders.

## Introduction

B-1 B cells, and specifically B-1a cells that express CD5, are the primary source of “natural” antibodies (Abs) whose poly/autoreactive features provide homeostatic protection against bacterial and viral pathogens (1–3). Natural Abs also serve housekeeping functions by clearing apoptotic cells and neo self-antigens (Ags) (4). B-1a cells home to the spleen, as well as the peritoneal (PeC) and pleural cavities (1, 5), where a remarkably large proportion (~5–15%) express stereotypic B cell receptors (BCRs) restricted to V_H_11 and V_H_12, which both recognize phosphatidylcholine (PtC), a determinant present in cell membranes and certain bacteria (6–8). Consequently, the Ab repertoires of B-1a cells have a biased composition. Due to a lack of Tdt mediated N-addition during fetal life (9, 10), they generate germline-related Abs encoding CDR-H3 segments that are more hydrophobic than B-2 repertoires (11, 12). Splenic marginal zone (MZ) B cells similarly possess innate-like defense properties and fetal-related V_H_ repertoires enriched in charged CDR-H3 segments (11, 13, 14). The propensity to generate autoreactive Abs has thus implicated innate-like MZ and B-1 B cells in the pathogenesis of autoimmunity (AI) and malignancy, including the most common leukemia in Western countries, chronic lymphocytic leukemia (CLL) (15–17).

The extrafollicular localization and constrained repertoires of B-1 cells are in marked contrast to B-2 cells that participate in T cell-dependent responses in secondary lymphoid tissues and generate affinity-matured, highly-diversified Abs. However, the developmental origins and regulatory mechanisms governing the selection of B-1 cells have been discussed. The “lineage” hypothesis, proposes that B-1 cells differentiate from discrete progenitors chiefly during fetal development and early ontogeny (5, 18, 19). Alternatively, the “selection” hypothesis posits that the Ag reactivity of the BCR mediates lineage specification and compartmentalization (20, 21).

B-1a B cells mainly derive from the fetal liver (FL) and neonatal tissues, but can develop, albeit less efficiently, from adult bone marrow (BM) where B-2 differentiation predominates (1, 5, 19, 22–24). A temporal and anatomic switch in primary B cell development is mediated by the Let7-Lin28b-Arid3a axis that differentially regulates B-1 development during fetal vs. adult life (25–27). Several transcription factors can preferentially influence the B-1 pathway (28, 29). However, a model linking the cellular origins with selection mechanisms that generate and shape the characteristic poly/autoreactive repertoires of B-1a cells remains an important subject of investigation.

Based on conventional B-2 selection models, the pre-BCR establishes a critical checkpoint to promote the emergence of IgM heavy chains (μHC) with tyrosine-enriched CDR-H3 rearrangements, rather than charged or hydrophobic loops that could harbor self-reactivity (30–32). Hence, Hardy postulated that μHCs from natural Abs, and V_H_11 in particular (7, 33), pair less efficiently with the surrogate light chain (SLC) concluding that pre-BCR selection likely differs during the fetal vs. adult stages of ontogeny. Indeed, V_H_11 interactions with the SLC appear weaker than other V_H_ segments (34). There is also evidence that the initial waves of B lymphopoiesis may proceed independently of the SLC during embryogenesis and that kappa light chain expression can occur prior to heavy chain rearrangements (35, 36). Accordingly, recent work by the Skok group found that B-1 progenitor μHCs, including V_H_12 segments, may bypass pre-BCR selection by pairing with prematurely rearranged light chains (37).

Members of the Fc receptor-like (*FCRL*) gene family in humans and mice encode type I transmembrane glycoproteins with tyrosine-based activation (ITAM)-like or inhibitory (ITIM) motifs, are preferentially expressed by B lineage cells, and modulate BCR-mediated signaling (38). In mice, FCRL1 is a pan B cell marker that promotes BCR signaling via an intracellular ITAM-like sequence (39, 40). However, FCRL5 has a more selective expression pattern and distinctly marks innate-like splenic MZ and PeC B-1 B cells (41). Its possession of both cytoplasmic ITAM-like and ITIM sequences fosters subset-specific inhibitory influence on BCR signaling in these two cell types (42). Recent work has also detected FCRL5 on subpopulations of memory B cells in malaria models (43, 44). While the *Fcrl6* gene in mice was identified several years ago (45), little is known about its biology. Notably, the first FCRL family member identified was a rat FCRL6 ortholog, termed gp42 (46). Gp42 was discovered in a search for markers of lymphokine activated killer (LAK) cells and shares a similar pattern of expression to human FCRL6 by cytotoxic NK and T cells, but not B cells (46, 47).

Here we found that expression of Fc receptor-like 6 (FCRL6) distinguished subpopulations of B cell progenitors throughout ontogeny that correlate with fetal vs. adult B-1a developmental potential. FCRL6^+^ FL and BM pro B cells exhibited protracted differentiation and proliferation, including the generation of nascent μHCs harboring constrained diversity and autoreactive properties. Furthermore, FCRL6 discriminated pre-BCR dependent and independent selection pathways in B cell progenitors that differentially parallel V_H_11 and V_H_12 B-1a cell development. FCRL6^+^ progenitors exhibited distinct transcript signatures including attributes of TCF/LEF and nervous system developmental regulation as well as B-1a related defense, migration, and differentiation properties. These findings provide new insight into the heterogeneous origins and selection mechanisms underlying innate-like B cell and B-1a development and have implications for AI and CLL pathogenesis.

## Materials and Methods

### Mice

BALB/cJ and C57BL/6J, as well as μMT and *Rag2*^−/−^ mice on the C57BL/6 background, were purchased from Jackson Laboratories and bred and maintained in animal facilities at the University of Alabama at Birmingham (UAB). *Dntt*^−/−^ (Tdt), *Igll1*^−/−^ (λ5), and *Rag1*^−/−^ mice on the BALB/c background were generously provided by Dr. Harry Schroeder Jr. at UAB. Eμ-TCL1 Tg mice were kindly provided by Dr. Carlo Croce at Ohio State University. Unless otherwise specified, 8–12 week-old female mice were used for these studies. Embryonic fetal livers (FL) were obtained from timed pregnancies. Vaginal plug formation after mating was counted as day 0. All studies and procedures were approved by the UAB Institutional Animal Care and Use Committee (IACUC).

### Quantitative PCR

Total RNA was extracted from mouse tissues and single cell suspensions using RNeasy Plus kits (Qiagen). cDNAs were generated using SuperScript II (Invitrogen). cDNAs were mixed with primer pairs and amplified using SYBR green master mix and the 7900HT Fast real-time PCR system (Applied Biosystems). Iμ, μ0, and *Myc* primers have been published (48–50). *Fcrl6* qPCR primers were designed to hybridize with the first extracellular domain using primer express software (Applied Biosystems). Samples were normalized to *Polr2a (RNA polymerase II*) expression (51). Primer sequences were as follows:

*Fcrl6* F: 5′-CATGCTGCTCTGGATGGTTCT-3′
*Fcrl6* R: 5′-AGCTCAGGATTTGGGAACAACTC-3′
Iμ F: 5′-GGATACGCAGAAGGAAGGC-3′
Iμ R: 5′-GGTCATTACTGTGGCTGGAGAG-3′
μ0 F: 5′-TGCAGGTTCCTCTCTCGTTTCCTT-3′
μ0 R: 5′-TGGGCCCATCTGTAGGATGGTAAT-3′
*Myc* F: 5′-AACAGGAACTATGACCTCG-3′
*Myc* R: 5′-AGCAGCTCGAATTTCTTC-3′
*Polr2a* F: 5′-GACTCACAAACTGGCTGACAT-3′
*Polr2a* R: 5′-TACATCTTCTGCTATGACATGGG-3′

### Generation of Anti-mouse FCRL6 Antibodies (Abs)

Rat anti-mouse FCRL6-specific mAbs, 1C3 (IgG1κ) and 3C1 (IgG2aκ), were generated using *Escherichia coli*-derived His-tagged recombinant protein comprised of the two extracellular Ig domains of mouse FCRL6. Respective cDNA regions were PCR amplified and cloned into the pET24b vector (Novagen) for bacterial expression, as previously described (52). Fisher rats (Jackson Laboratory) were immunized at 3–4 day intervals over a 3 week period and popliteal nodes were fused with the mouse plasmacytoma Ag8.653 and plated for selection in 96-well-plates (41). At 10–14 days after fusion, hybridoma clones were screened for specificity and cross-reactivity by staining hemagglutinin (HA)-tagged FCRL1, FCRL5 (C57BL/6 and BALB/c alleles), and FCRL6 TM transductants generated as previously (41). Rabbit anti-FCRL6 polyclonal Abs were generated by hyperimmunizing New Zealand White rabbits (Charles River Laboratories) with *Escherichia coli*-derived His-tagged recombinant protein.

### Immunoprecipitation and Western Blotting

To analyze the molecular nature of FCRL6, HA-tagged BW5147 FCRL6 retroviral transductants (1 × 10^7^) were lysed in 1% NP-40 lysis buffer. Whole cell lysate proteins were quantitated using the BCA reagent (Pierce) and incubated at 4°C for 30 min with rat anti-mouse FCRL6 (3C1) or an isotype-matched control (rat IgG2aκ) mAb, followed by the addition of 30 μl of a 50% slurry of protein G beads (GE Healthcare), and incubation overnight at 4°C. The beads were washed five times with 1 ml of lysis buffer to reduce non-specific binding, resuspended in an equal volume of SDS sample buffer, and boiled. Proteins were resolved by SDS-PAGE and transferred to PDVF membranes (Millipore) before immunoblotting with anti-HA (12CA5, Roche) or rabbit anti-mouse FCRL6 polyclonal Abs, followed by rabbit anti-mouse or goat anti-rabbit HRP (Southern Biotech). Bound Abs were visualized using the ECL reagent (GE Healthcare) and detected by Biomax XAR film (Kodak).

### Flow Cytometry and Cell Sorting

Spleen, FL, and bone marrow (BM) cells were prepared as single cell suspensions after red blood cell lysis with ACK lysing buffer (Gibco). PeC cells were prepared by lavaging with complete 10% FCS RPMI-1640 medium. FL were obtained from timed pregnancies. Neonatal livers, spleens, and BM were harvested at specific days after birth. Cells were stained with antigen–specific or isotype-matched control Abs (Supplementary Table—Key Resources) after blocking with unlabeled anti-CD16/32 for 5 min.

AF647 and biotin-conjugated anti-mouse FCRL6 (1C3 and 3C1) were generated and labeled by our laboratory (Invitrogen and Thermo Scientific). Anti-V_H_11 [3H7 (7); a gift from K. Hayakawa], anti-V_H_12 [5C5 (53); a gift from K. Rajewsky], and anti-V_H_7 [TC68 (54), a gift from J. Kearney] were biotinylated in our laboratory (Thermo). Cells were analyzed using FACSCalibur (BD) or LSRII (BD) flow cytometry instruments and plotted with FlowJo software (Treestar).

For cell sorting, leg bones were pooled from 5 to 10 adult male and female mice and 7–10 FL were pooled from E18 fetuses isolated from the wombs of pregnant mothers according to the timing of vaginal plug formation. Single cell suspensions from the BM of BALB/cJ mice were prepared by flushing the femur and tibia bones with a 29 G needle syringe. Single suspensions of FL cells were prepared by crushing the tissue with a 1 ml syringe plunger and passage through a 40 μm strainer (BD). Single cell suspensions were treated with ACK lysing buffer and re-suspended with PBS containing 2% FCS. After Fc blockade with unlabeled anti-CD16/32, cells were stained with fluorochrome-labeled mAbs [FITC-CD43, BV421-CD19, PE-B220, PECy7-AA4.1/CD93, and Alexa647 FCRL6 (1C3)], as well as biotin-labeled mAbs [CD3 (145-2C11), Mac1 (M1/70), Gr1 (RB6-8C5), Ly6C (HK1.4), Ter119, and IgM (RMM-1)] to exclude T, myeloid, and erythrocyte lineage cells and immature/mature B cells, and counterstained with SA-BV570. FCRL6^+^/FCRL6^−^ pro B (AA4.1^+^Lin^−^CD43^+^B220^+^CD19^+^IgM^−^) cells from the FL and BM were sorted using a FACSAria II sorter (BD).

### *In vivo* Proliferation and Cell Cycle Analysis

Single cells were prepared from the FL and BM of BALB/cJ mice at 24 h after E17 pregnant dams or adult mice were injected i.p. with BrdU (twice at 12 h intervals). Single cell suspensions were stained with anti-mouse AA4.1, CD43, CD19, B220, IgM, and FCRL6 (1C3) for surface detection, then fixed and permeabilized, treated with DNase I, and stained with anti-BrdU and 7AAD to examine proliferation and cell cycle status. Stained cells were analyzed by FACS with an LSRII instrument and profiles were plotted with FlowJo software.

### Intracellular Staining

Single cells from FL and BM were stained for AA4.1, CD43, CD19, B220, IgM, and FCRL6 (3C1), and fixed with Cytofix (BD) for 15 min on ice, then permeabilized with Foxp3 Fixation/Permeabilization buffer (eBio) for 30 min on ice, and stained for either Ki-67, c-Myc, NFAT2, Ikaros, or Aiolos, along with F(ab′)_2_ goat anti-mouse IgM for 1 h at room temperature. Cells were examined using an LSRII cytometer and plotted with FlowJo software.

### Phospho-Flow Analysis

FACS sorted FCRL6^+^ and FCRL6^−^ pro B (CD43^+^CD19^+^B220^hi^IgM^−^) cells from adult BM were treated with the phosphatase inhibitor pervanadate (NaVO_4_) for 10 min. Stimulated cells were fixed with prewarmed Phosflow Lyse/Fix buffer (BD) at 37°C for 10 min and permeablized with Phosflow Perm Buffer III (BD). After Fc blockade (CD16/32), cells were stained with anti-phospho ERK pT202/pY204, STAT5 Y694, or isotype control mAbs (BD) for 30 min at room temperature. Phosphorylation was analyzed using a FACSCalibur flow cytometer (BD) and plotted with FlowJo software. The fold induction change in phosphorylation for FCRL6^−^ and FCRL6^+^ pro B cells was calculated by comparing the MFI ratios of FCRL6^−^/FCRL6^+^ pro B cells with and without stimulation.

### Pre-BCR and Intracellular IgM Staining

FCRL6^+^ and FCRL6^−^ pro Bcells (AA4.1^+^CD43^+^CD19^+^B220^hi^IgM^−^) stained for cell surface markers, were fixed with Cytofix/Cytoperm buffer (BD) for 20 min on ice and stained with anti-pre-BCR (SL156) and/or F(ab′)_2_ goat anti-mouse IgM for 1 h at room temperature. Cells were analyzed using an LSRII instrument and plotted with FlowJo software.

### Apoptosis Assays

Single cells from the FL and BM were stained for cell surface markers then washed twice in Annexin V binding buffer, followed by staining with anti-Annexin V for 15 min at room temperature. After incubation with the Annexin V binding buffer, cells were analyzed using an LSRII instrument and plotted with FlowJo software.

### *In vitro* Proliferation Assays

FACS-sorted FCRL6^+^ and FCRL6^−^ pro Bcells (AA4.1^+^CD43^+^CD19^+^B220^hi^Lin^−^IgM^−^) from BM were washed twice with complete RPMI 1640 media containing 10% FCS. Pro B cells (10^3^ cells per well) were loaded into round-bottom 96-well-plates in triplicate, in the presence or absence of different concentrations of TSLP (0–100 ng/ml). Cells were incubated at 37°C in a CO_2_ incubator for 4 days, total cell numbers from each well were counted, and live cells were enumerated by flow cytometry analysis using a FACScalibur instrument with dead cell exclusion by PI.

### Progenitor Differentiation Assays

**Figure 1:**
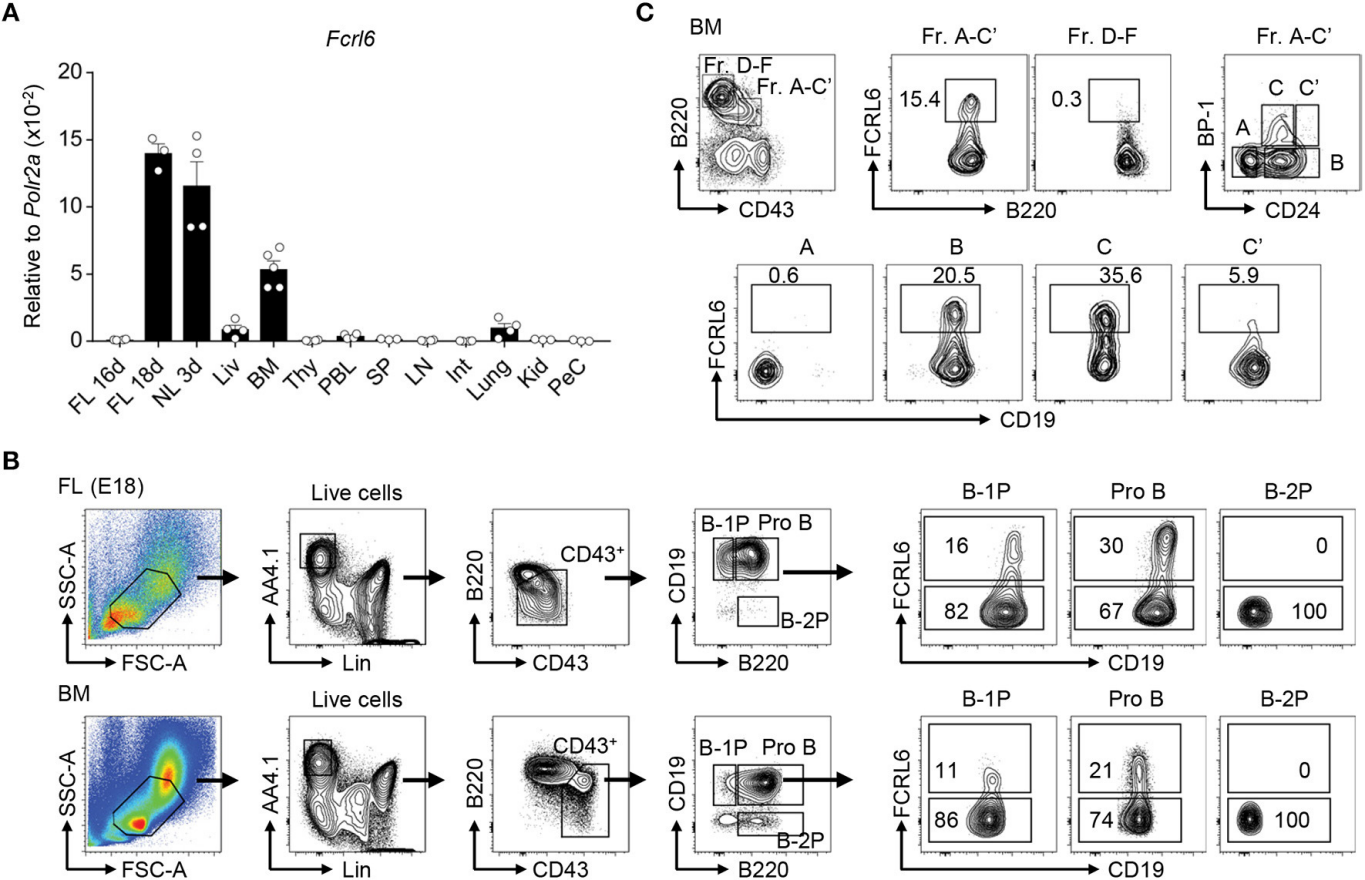
*Fcrl6* is restricted to primary B lymphopoietic tissues and segregates B lineage progenitors. **(A)** RQ-PCR analysis of *Fcrl6* expression in BALB/cJ tissues including embryonic day 16 (E16) and 18 (E18) fetal liver (FL), 3 day neonatal liver (NL), and adult (all others) liver (Liv), bone marrow (BM), thymus (Thy), peripheral blood leukocytes (PBL), spleen (SP), lymph node (LN), small intestine (Int), lung, kidney (Kid), and peritoneal cavity lavage cells (PeC). Primers were specific for regions of exons 1–3 encoding the signal peptide and first extracellular domain. Samples were normalized to *RNA polymerase II* (*Polr2a*) expression. **(B)** Flow cytometry with a receptor-specific mAb (1C3) analyzing FCRL6 surface expression by B-1 progenitor (B-1P), B-2 progenitor (B-2P) and pro B cell subpopulations in E18 FL and adult BM according to the scheme of (19). Numbers within the gates indicate the percentage of cells. **(C)** Flow cytometry analysis of adult BM B cell fractions (Fr) according to the differentiation scheme of (56). Numbers adjacent to the gates indicate the percentage of cells. Data shown are cumulative from the tissues of 3–5 mice amplified in duplicate **(A)**, representative of more than five independent experiments **(B)**, or three independent experiments **(C)**.

Stromal cell-dependent B cell progenitor differentiation assays were previously described by Montecino-Rodriguez et al. and performed as follows (19, 55). S17 stromal cells were freshly thawed and grown in complete RPMI with 10% FCS for 7–10 days. S17 cells were detached with 0.25% Trypsin-EDTA solution (Gibco), filtered with a 70 μm cell strainer (BD), and 2.5 × 10^4^ cells were added to 6-well-Biocoat Transwell insert plates (BD) for growth on inserts overnight. FACS-sorted FL or BM B-1P and pro B cells (0.5–1 x 10^4^ cells/well), gated as in Figure 1B, were added to the bottom chamber of the Transwell plate, separated from S17 stromal cells in the upper chamber, and cultured in αMEM media (Gibco) with 5% FCS and a cytokine cocktail including TSLP (10 ng/ml), IL-3 (5 ng/ml), IL-6 (10 ng/ml), SCF (10 ng/ml), and Flt3L (10 ng/ml). At day 4, 25% of the upper and lower culture media was replaced with fresh cytokine-containing media. At day 8, the cells were harvested and washed with PBS containing 2% FCS. Viability, proliferation, and differentiation into immature B220^+^IgM^+^ B cells were examined with an LSRII cytometer (BD) and analyzed with Flow-Jo software.

### *Ighv* Repertoire Sequencing and Analysis

RNA from FCRL6^+^ to FCRL6^−^ pro B cells sorted as in Figure 1B, from pooled E18 FL (*n* = 7) or adult BM samples (*n* = 8) from BALB/c mice, was isolated using TRIzol (Invitrogen) reagent and used to generate cDNA with the High-Capacity cDNA synthesis kit (Applied Biosystems). cDNAs were used as a template for PCR using the KOD high fidelity polymerase (Novagen) and a mixture of 8 V_H_ family and IgM-specific primers (listed below) containing 5′ adaptors to allow barcoding by the Illumina TruSeq multiplex PCR kit (Illumina). Resulting PCR products were subjected to 10 cycles of PCR using Illumina TruSeq indexing primers. The resulting amplicons were purified using AMPure (Beckman), submitted to the UAB Heflin Genomics core, and sequenced using Illumina's MiSeq Reagent Kit v3 2X300 kit in a Miseq next generation sequencer. The resulting FastQ files were filtered for quality (>Q30) and assembled using the vsearch suit of informatic tools (57). Fasta files containing the full length paired *Igh* sequences were submitted to the IMGT High-VQuest server for *Ighv, Ighd*, and *Ighj* identification, as well as *Ighv* junction and other bioinformatic analyses (58). IMGT files were visualized using R software (R Foundation for Statistical Computing) and the following software packages: vegan, plyr, plots, ggplots, ggrepel, ggseqlogo, pbmcapply, seqinr, and stringr. Some rarefaction curves were replicated with VDJtools. Sequences were deposited at NCBI accession: PRJNA547609.

*Ighv*-specific primer sequences:

An i5 linker (5′-CCCTACACGACGCTCTTCCGATCT-3′) was added to the 5′ end of the forward V_H_ primers and an i7 linker (5′-ATCTCGTATGCCGTCTTCTGCTTG-3′) to the 5′ end of the reverse Cμ primer.

VH1a F: 5′-CAGGTGTCCACTCCCAGGTCC-3′
VH1b F: 5′-CAGGTGTCCTCTCTGAGGTCCAG-3′
VH2/4 F: 5′-CAGGTGTCCTATCCCAGGTGCAGC-3′
VH3 F: 5′-GAAGGTATCCTGTCTGATGTGCAGC-3′
VH8 F: 5′-CAGATGTCCTGTCCCAGGTTACTC-3′
VH9/11/12 F: 5′-CAGGTGCCCAAGCACAGATCCAG-3′
VH5/6/7/10/13F:5′-CAGGTGTCCAGTGTGAAGTGCAGC-3′
VH14 F: 5′-CAGAGGTTCAGCTGCAGCAGTCTGG-3′
Cμ R: 5′-AGGGGGAAGACATTTGGGAAGGAC-3′

### Sequence Analysis of the CDR-H3 Regions

CDR-H3 analysis was performed as in Khass et al. (32). Briefly, the CDR-H3 was defined as the sequence starting immediately after the cysteine at the end of FR3 at position 96 and then through, but not including, the tryptophan that begins FR4. At positions 99 to 103, we calculated the frequency of amino acids for individual B cell subsets and common *Ighv* segments. Logo plots were prepared with the ggseqlogo R package (59).

### RNA-Seq Library Preparation and Analysis

RNA sequencing (RNA-seq) was performed at the UAB Heflin Center for Genomic Science. E18 FL and adult BM FCRL6^+/−^ pro B cells were sorted in duplicate in four independent experiments from BALB/cJ mice by flow cytometry as detailed above and shown in Figure 1B. mRNA was isolated using RNeasy Plus kits (Qiagen) and sequencing was performed on a Illumina NextSeq 500 (paired-end sequencing 2 × 75 bp) according to the manufacturer's guidelines. Briefly, the quality of the total RNA was assessed using the Agilent 2100 Bioanalyzer (all samples had RIN values of >9.9) followed by two rounds of poly(A) selection and conversion to cDNA. TruSeq library generation kits were constructed by random fragmentation of the polyA mRNA, followed by cDNA production using random primers (Agilent Technologies). The ends of the cDNA were repaired, A-tailed, and adaptors were ligated for flow cell attachment, sequencing, and indexing as per the manufacturer's instructions (Agilent Technologies). cDNA libraries were quantitated using qPCR in a Roche LightCycler 480 with the Kapa Biosystems kit for library quantitation (Kapa Biosystems) prior to sequencing.

All samples contained a minimum of 32 million reads with an average number of 42.9 million reads across all biological replicates. The FASTQ files were uploaded to the UAB High Performance Computer cluster for bioinformatics analysis with the following custom pipeline built in the Snakemake workflow system (v4.8.0) (60): first, quality and control of the reads were assessed using FastQC, and trimming of the bases with quality scores of <20 was performed with Trim_Galore! (v0.4.5). Following trimming, the reads were aligned with STAR (61) (v2.5.2a, during “runMode genomeGenerate,” the option “sjdbOverhang” was set to 74) to the Ensembl mouse genome (mm10), which resulted in an average of 80.3% uniquely mapped reads. BAM file indexes were generated with SAMtools (v1.6) and gene-level counts were generated using the function “featureCounts” from the R package, Rsubread (v1.26.1; r-base v3.4.1), with the “Mus_musculus.GRCm38.90.gtf” file from Ensembl. The parameters used in “featureCounts” included: isGTFAnnotationFile = TRUE, useMetaFeatures = TRUE, allowMultiOverlap = FALSE, isPairedEnd = TRUE, requireBothEndsMapped = TRUE, strandSpecific = 2, and autosort = TRUE. Additional parameters were kept as default. Logs of reports were summarized and visualized using MultiQC (v1.5) (62).

Count normalization and differential expression analysis were conducted with R software with the DESeq2 package (63) (v1.16.1). Following count normalization, principal component analysis (PCA) was performed (**Figure 3A**) and genes were defined as differentially expressed genes (DEGs) if they passed a statistical cutoff containing an adjusted *P* < 0.05 [Benjamini-Hochberg False Discovery Rate (FDR) method] and if they contained an absolute log_2_ fold change ≥1. Functional annotation enrichment analysis was performed in the NIH Database for Annotation, Visualization and Integrated Discovery (DAVID, v6.8) by submitting all DEGs identified. The Benjamini-Hochberg FDR correction was also applied to determine gene ontology (GO) terms with the cutoff of an adjusted *P* < 0.05.

RNA-seq files are available at the Gene Expression Omnibus under accession number GSE132438. Figures, including the heatmap, scatter plots, and volcano plot, were made with R software using the following packages: ggplot2 (v3.1.0) and ComplexHeatmap (v1.20.0).

### Statistical Analysis

Statistical analysis was performed using GraphPad Prism 7 software. A paired Student's *t*-test was used for comparing two experimental groups (FCRL6^+^ and FCRL6^−^ progenitors) for apoptosis, intracellular and surface staining, RQ-PCR, and differentiation assays. Unpaired Student's *t*-test was used for comparing cell populations in mutant mouse studies. Statistical analyses used in RNA-seq studies are detailed in the indicated section.

## Results

### FCRL6 Segregates Subsets of B Cell Progenitors Throughout Ontogeny

Following the discovery of human *FCRL1-6* (38, 64), we identified a mouse *Fcrl6* counterpart encoding putative type I transmembrane (TM), glycosylphosphatidylinositol (GPI)-linked, and secreted isoforms with two extracellular Ig-like domains (45). By RQ-PCR, we detected *Fcrl6* transcripts in primary lymphopoietic tissues, including embryonic day 18 (E18) FL, day 3 postnatal liver, and adult BM (Figure 1A). The generation of monoclonal Abs (mAbs) yielded two FCRL6-specific subclones (1C3 and 3C1) and identified that the TM isoform had a molecular weight of ~42 kD (Supplementary Figures 1A,B). Although FCRL6 is confined to natural killer (NK) and T cells in humans (47), by flow cytometry analysis from adult BALB/cJ BM, FCRL6 was restricted to early stage B cells (Supplementary Figure 1C and data not shown). Expression was not detected by other lineages or in any other hematopoietic tissue at homeostasis. FCRL6 shared a similar pattern of expression by FL and BM B cells bearing CD19 and B220 as well as AA4.1 and CD43, but little if any surface IgM (Supplementary Figures 2A,B). These observations indicated that 11–12% of FL and 1–2% of BM CD19^+^ B cells express FCRL6.

Because its frequency was about 10 times greater in the FL, we examined FCRL6 using a differentiation scheme that segregates precursors capable of reconstituting the B-1 or B-2 lineage (19). FCRL6 marked subsets of CD19^hi^B220^hi^ pro B cells in the FL and BM, as well as some CD19^hi^B220^lo^ B-1 precursors (B-1P), which are more frequent in the FL, but not CD19^lo^B220^+^ B-2 precursors (B-2P) that chiefly populate the BM (Figure 1B). In day 3 and 7 neonatal tissues, FCRL6^+^ B cells variably expanded in frequency with time in the liver, BM, and spleen (Supplementary Figure 2C). This pattern of tissue expression demonstrated that FCRL6 segregates subsets of progenitor B cells conserved throughout life.

We then examined adult BM according to Hardy's definition (56). By this scheme, only a minor subset of B cell progenitors, estimated at 7–15% of the total A-C′ fraction (Fr), expressed FCRL6 (Figure 1C). Further separation based on CD24/HSA and BP-1 disclosed hardly any FCRL6^+^ Fr. A cells, but the frequency increased in Fr. B, peaked in Fr. C, declined in Fr. C′, and very few cells were found beyond the Fr. D stage. Thus, FCRL6 expression was tightly regulated and predominantly restricted to Fr. B and C pro and pre B cells, developmental stages during which V(D)J recombination and pre-BCR selection occur.

### FCRL6 Progenitors Exhibit Features of Protracted Differentiation

**Figure 2:**
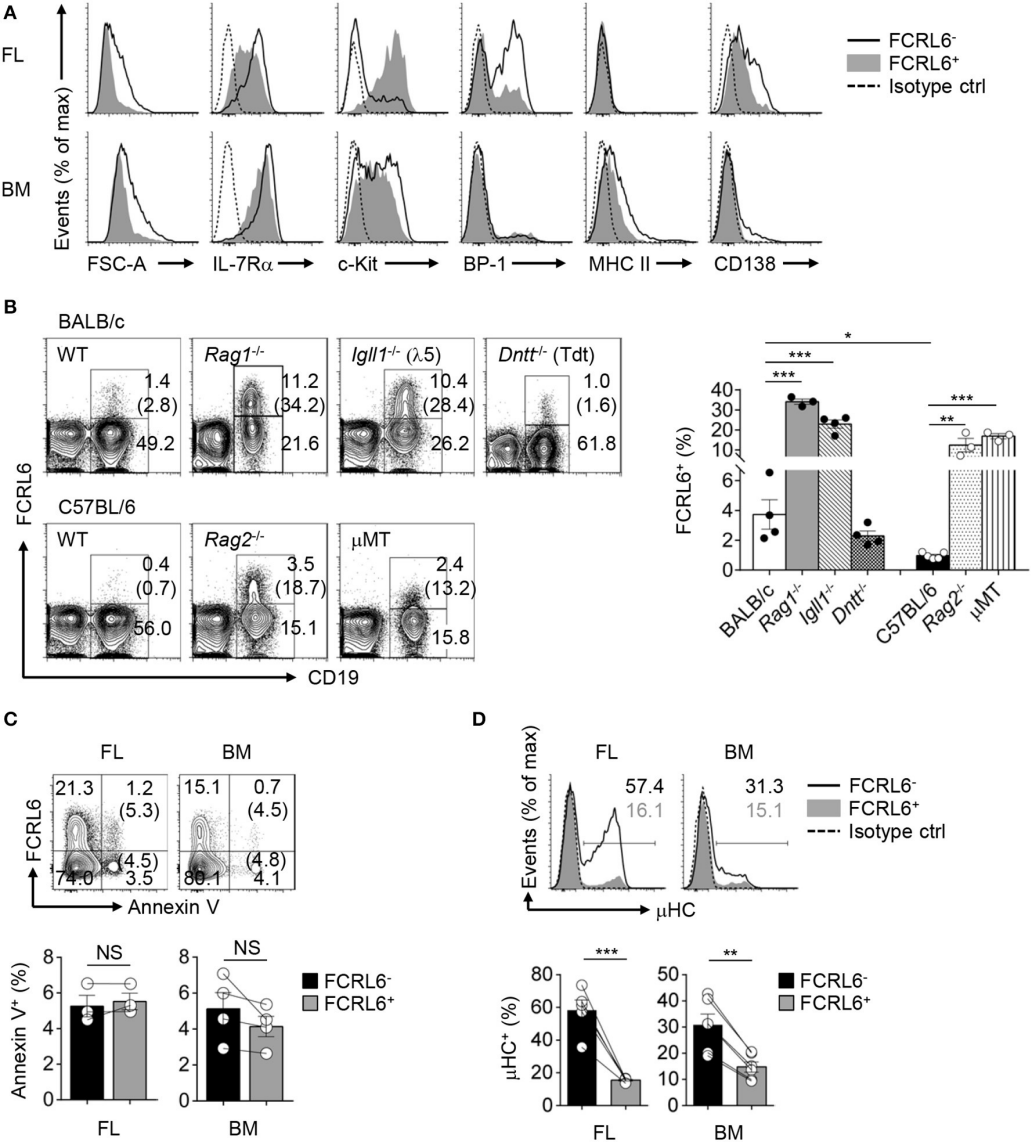
FCRL6^+^ pro B cells exhibit protracted development, expand in *Igh* defective mice, and generate little cytoplasmic IgM (μHC). **(A)** Flow cytometry analyzing FCRL6^+^ and FCRL6^−^ pro B cells in E18 FL (above) and adult BM (below) to assess cell size by forward scatter (FSC) and co-expression of indicated surface markers. **(B)** Flow cytometry of total BM cells isolated from adult WT BALB/c and C57BL/6 mice or genetic mutants (*n* = 3–5 per strain) on related background strains stained for FCRL6 and CD19 as well as IgM to dump immature/mature B cells. Numbers adjacent to the gates indicate the percent of cells among total lymphocytes. The frequency of FCRL6^+^/CD19^+^ B cells is indicated in parentheses and summarized in the bar graph to the right. **(C)** Survival differences of FL and BM FCRL6^+^ and FCRL6^−^ pro B cells evaluated by Annexin V staining and flow cytometry. The frequency of FCRL6^+^ or FCRL6^−^ Annexin V^+^ pro B cells is indicated in parentheses. **(D)** Representative intracellular IgM staining of FL and BM FCRL6^**−**^ and FCRL6^+^ pro B cells. Each symbol represents an individual mouse. Small horizontal lines **(B–D)** indicate s.e.m. NS, not significant (*P* > 0.05); *P < 0.05; **P < 0.01; ***P < 0.001 as determined by unpaired **(B)** or paired **(C,D)** Student's *t*-test. Data are representative of at least two experiments **(A–C)**, or more than 10 independent experiments **(D)**.

We next analyzed other phenotypic features in the FL and BM. By forward scatter, FCRL6^+^ pro B cells were smaller than FCRL6^−^ cells, suggesting they might proliferate more slowly (Figure 2A). The relatively lower density of CD127/IL-7Rα on FCRL6^+^ cells in both tissues indicated they might also be less responsive to IL-7. The early differentiation marker CD117/c-kit was uniformly upregulated by the FCRL6^+^ subset in FL, but not in the BM. BP-1 was also less abundant on FL FCRL6^+^ cells. Finally, MHCII and CD138, which can distinguish B-2 progenitors (65), were slightly higher on FCRL6^−^ pro B cells in the BM than FL. Together these results indicated that FCRL6 distinguished less differentiated pro B cells.

We then compared different mouse strains and models with defects in early B cell development. We first observed that the frequency of CD19^+^FCRL6^+^ cells in the BM was about 4-fold higher in WT BALB/c than C57BL/6 mice (Figure 2B). In *Rag1*^−/−^*, Rag2*^−/−^, and *Igll1* (λ5)^−/−^ mutants, B cell development is blocked at Fr. C and C′ resulting in the enrichment of Fr. B and C pro B cells. BM cells from *Rag1*^−/−^*, Rag2*^−/−^, *Igll1*^−/−^, and μMT mice of respective strains all had expanded frequencies of FCRL6^+^ B cells. In these models, CD19^+^FCRL6^+^ B cells were ~10 to 15-fold greater compared to WT mice. However, no difference was evident in *Tdt*^−/−^ mice. Thus, FCRL6^+^ cells emerged prior to RAG1/2 expression, and moreover V(D)J recombination, segregated a subset of pro B cells that varied by genetic background, and expanded following disruption of *Igh* rearrangement or pre-BCR/BCR assembly.

Because FCRL6^+^ pro B cells were relatively smaller and less differentiated, it was possible they represented defective cells destined for elimination. However, no differences in Annexin V reactivity, and thus survival status, were found (Figure 2C). We then analyzed their capacities for generating cytoplasmic μHC. Compared to FCRL6^−^ progenitors, frequencies of μHC^+^FCRL6^+^ cells were nearly 4-fold lower in the FL and 2-fold lower in the BM (Figure 2D). These results indicated marked mechanistic differences in μHC generation according to FCRL6 status.

### FCRL6 Defines Distinct Transcriptomic and Biologic Heterogeneity

**Figure 3:**
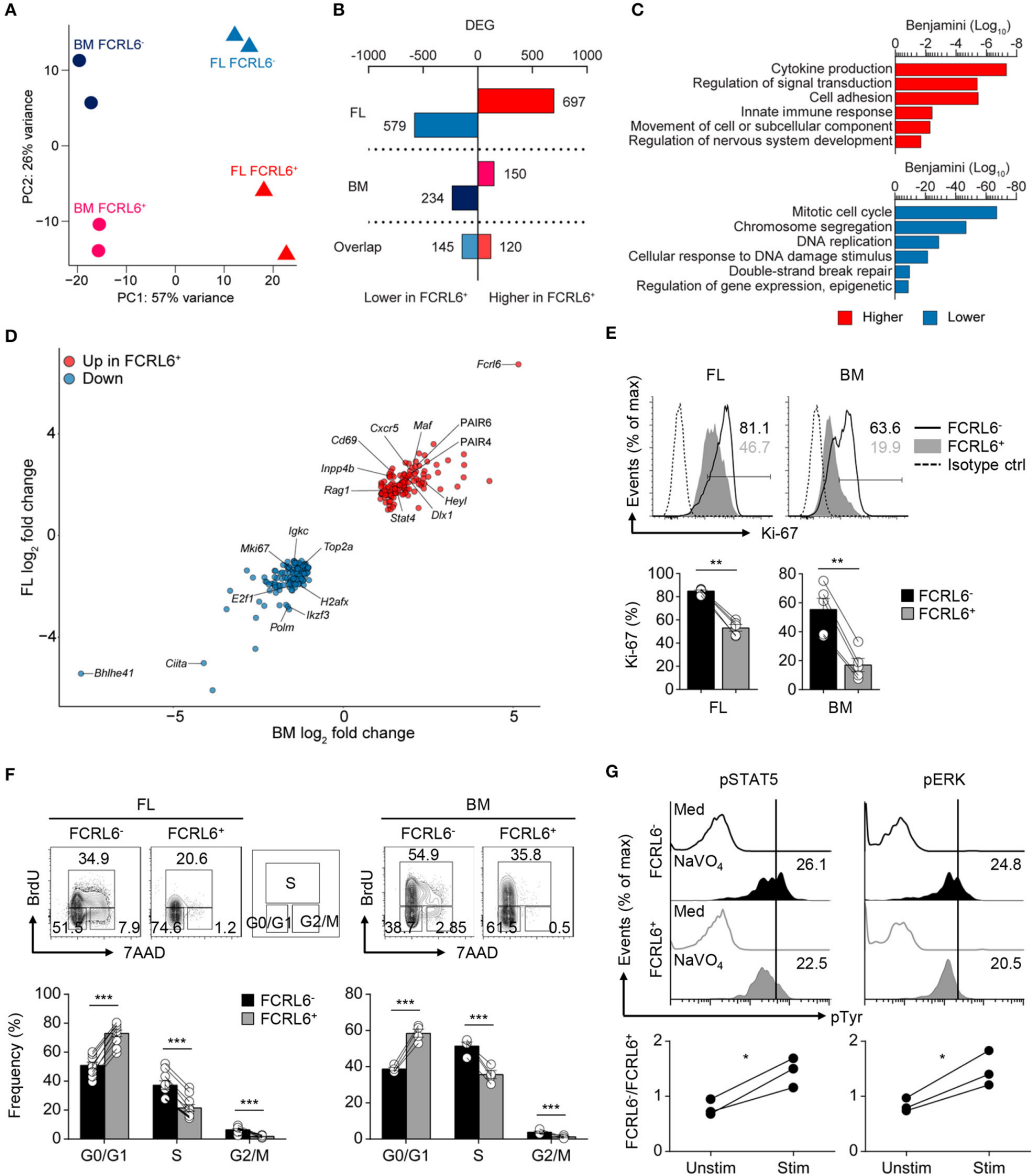
Transcriptomic and biologic heterogeneity of pro B cells. **(A)** Principal component (PC) analysis of RNA-seq performed on duplicate FL and BM FCRL6^+^ and FCRL6^−^ pro B cell samples sorted by flow cytometry as in Figure 1B. **(B)** Quantitation of differentially expressed genes (DEG) relative to FCRL6^+^ cells by tissue defined by a change in expression of 1-fold (log_2_ value) and FDR (<0.05). **(C)** Gene ontogeny (GO) pathways derived from FL DEGs. **(D)** Scatter plot of overlapping DEGs. Results are presented as the difference in expression (log_2_ transformed values) of FCRL6^+^ relative to FCRL6^**−**^ pro B cells from the FL (vertical) and BM (horizontal). **(E)** Intracellular staining and flow cytometry analysis of Ki-67 among pro B cell subsets (*n* = 5 each). Percentages of gated cells relative to an isotype-matched control are indicated in the histograms and summarized below. **(F)** Proliferation and cell cycle status of FL (*n* = 10) and BM (*n* = 4) pro B cell subsets assayed by staining with the thymidine analog BrdU and 7-AAD DNA intercalation dye before flow cytometry analysis. The percentages of G0/G1, S, and G2/M phase cells are shown adjacent to the contour plot gates and are summarized below. **(G)** Phospho (p)-flow cytometry analysis of sorted FCRL6^+^ and FCRL6^**−**^ BM pro B cells either unstimulated (medium) or stimulated with sodium pervanadate (NaVO_4_) for 10 min *ex vivo*. Cells were intracellularly stained with control Abs or those specific for pSTAT5 and pERK. Values in the histograms represent MFI ratios (test Ab MFI/control Ab MFI). Fold differences comparing FCRL6^−^ and FCRL6^+^ pro B cells are shown below. Each symbol **(E–G)** represents an individual mouse. Small horizontal lines **(E,F)** indicate s.e.m. *P < 0.05; **P < 0.01; ***P < 0.001 as determined by paired Student's *t*-test. Data are from four independent sorts and two per cell type **(A–D)**, one representative of two experiments **(E,F)**, and three independent experiments **(G)**.

We next performed high-throughput RNA sequencing (RNA-seq) to compare the gene expression profiles of FL and BM FCRL6^+^ to FCRL6^−^ pro B cells. By principle component analysis (PCA), duplicate samples clustered closely and segregated into four quadrants indicating distinct gene signatures for the four subsets (Figure 3A). This analysis identified a total of 1,276 differentially expressed genes (DEGs) in the FL and 384 in the BM [DEG criteria: a change in expression of 1-fold (log_2_ value); false discovery rate (FDR), < 0.05] (Figure 3B and Supplementary Figures 3A,B). By gene ontology (GO) analysis, cytokine production, adhesion, innate defense, and migration pathways were upregulated by FL FCRL6^+^ pro B cells (Figure 3C). Because these features were evocative of innate-like B cells, we compared DEGs from FL pro B cells with those from PeC B-1a and B-1b cells or splenic B-1a and MZ B cells extracted from the ImmGen database. Greater gene overlap (~1.5-fold) was observed with FCRL6^+^ upregulated DEGs (Supplementary Figure 3C). Distinct biologic differences related to FCRL6 expression were further implied by 265 DEGs that overlapped between the FL and BM (Figures 3B,D). Dissimilar proliferation kinetics in FCRL6^+^ cells were indicated by the strong repression of mitotic cell cycle genes, including transcripts for *Mki67* (encodes Ki-67). Intracellular staining confirmed higher frequencies of Ki-67^+^ FCRL6^−^ pro B cells (Figure 3E). Furthermore, an analysis of BrdU and 7-AAD status showed that most FCRL6^+^ cells had not entered the S/G2+M phase and were primarily in G0/G1 (Figure 3F). G2/M frequencies were 6.5 and 5.7-fold higher for FCRL6^−^ compared to FCRL6^+^ pro B cells in the FL and BM. These results demonstrated significantly lower proliferation and cell cycle activity for FCRL6^+^ cells *in vivo* regardless of their tissue of origin. By GO pathway analysis, genes for signal transduction regulation were also induced. Among DEGs encoding phosphatases, 14/20 in the FL and 4/5 in the BM were upregulated in FCRL6^+^ cells (Supplementary Figure 3D). We thus compared signaling features of sorted BM pro B cells by *ex vivo* treatment with the phosphatase inhibitor pervanadate. FCRL6^−^ cells had higher STAT5 and ERK phosphorylation (Figure 3G), indicating the diminished activation potential of FCRL6^+^ cells. Thus, in addition to other properties, FCRL6 defined pro B subsets with specific regulatory differences.

As expected, *Fcrl6* was the most upregulated overlapping DEG between tissues. *Bhlhe41*, a transcriptional repressor critical for B-1a development and Ab repertoire formation (28), proved the most downregulated (Figure 3D). Furthermore, GO pathway components associated with nervous system development were upregulated. These included overlapping DEGs (*Heyl, Dlx1, Aph1b*) and pathways relevant for Notch, Wnt, stem cell, EMT biology, and TCF/LEF (Supplementary Figure 3E). By flow cytometry, intracellular LEF1 expression was indeed elevated in FCRL6^+^ compared to FCRL6^−^ progenitors in day 3 neonatal tissues (data not shown). These findings collectively demonstrated divergent developmental and regulatory features for FCRL6^+^ progenitors.

### Disparate DNA Repair, *Ighv* Accessibility, and μHC Repertoires

**Figure 4:**
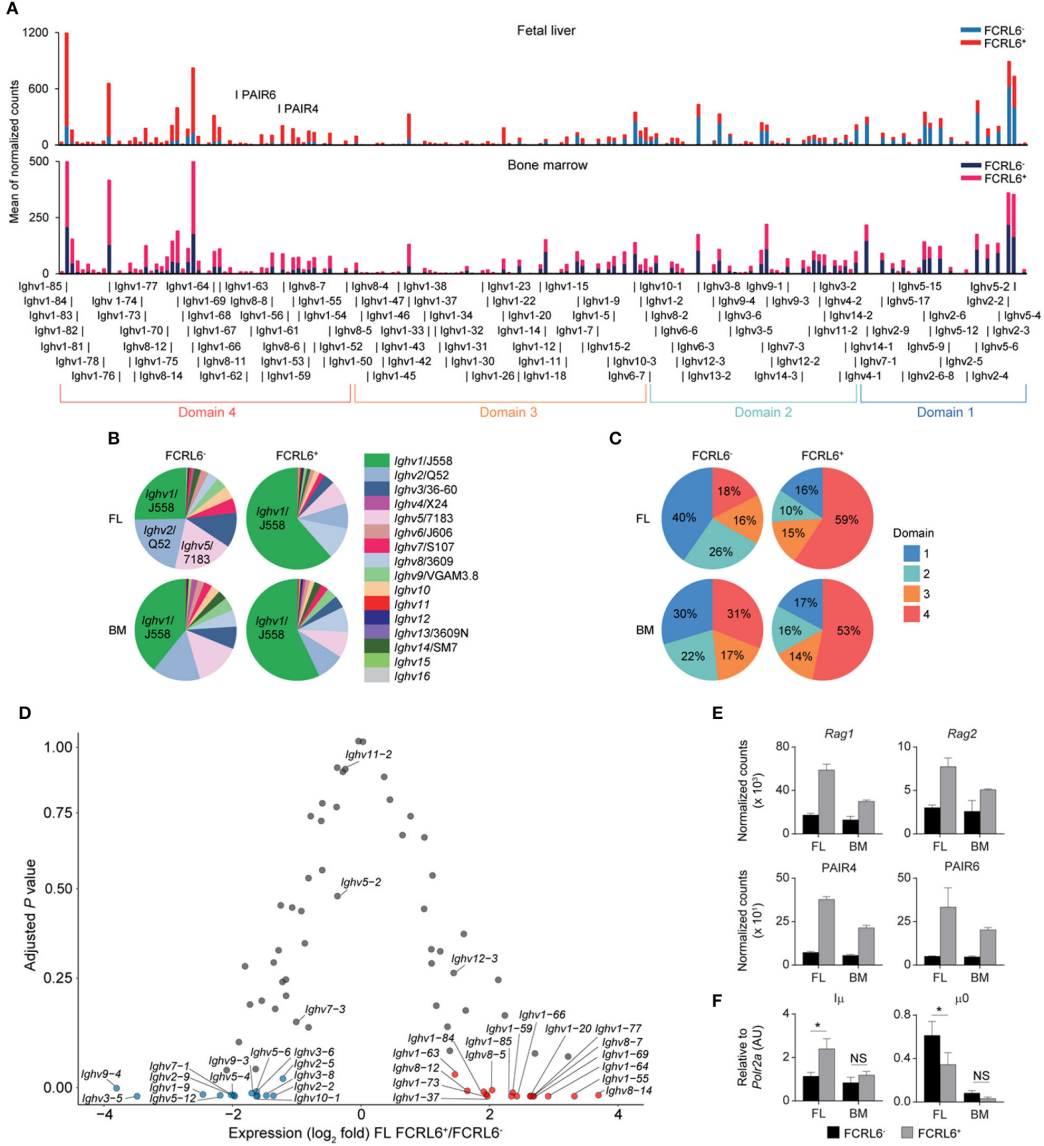
FCRL6 discriminates pro B cells with divergent *Ighv* locus accessibility. **(A)** Individual segment usage across the *Ighv* locus based on the means of normalized counts from duplicate FL and adult BM pro B cell subsets. The locus is segregated into four domains based on Choi et al. (68). The PAIR4 (ENSMUSG00000104373/Gm37511) and PAIR6 (ENSMUSG00000104373/Gm37511-Gm30996) ncRNA elements are indicated in domain 4. **(B)** Frequency of *Ighv* family usage among the four subsets based on the summation of the means of normalized counts from total *Ighv* segments of each subfamily. **(C)** Frequency of domain usage based on the summation of the means of normalized counts from grouped *Ighv* locus genes segregated as indicated in **(A)**. **(D)** Volcano plot detailing RNA-seq analysis of *Ighv* genes from sorted FL FCRL6^+^ and FCRL6^−^ pro B cells. Results are presented as the difference in fold expression (log_2_ transformed values) of FCRL6^+^ relative to FCRL6^−^ pro B cells and plotted against adjusted *P*-values. **(E)** RNA-seq analysis of coding and non-coding genes relevant to V(D)J rearrangement and locus contraction from the four FL and BM FCRL6^+^ and FCRL6^−^ pro B cell subsets. Note *Rag2* is among FL DEGs, while the other DEGs overlap between the FL and BM. **(F)** RQ-PCR of non-coding transcripts involved in initiating locus recombination relative to *Polr2a*. Small horizontal lines **(E,F)** indicate s.e.m. *P < 0.05 as determined by paired Student's *t*-test. Data are from four independent sorts and two per cell type as in Figures 3A–E and three independent sorts per subset performed in duplicate **(F)**.

Because V(D)J recombination and DNA damage repair are coupled to the cell cycle (66, 67), enrichment in the G0/G1 phase indicated that FCRL6^+^ pro B cells might be more actively undergoing HC rearrangement. Indeed, the RNA-seq GO pathway analysis revealed the downregulation of DNA damage repair programs (Figure 3C). To assess the spectrum of transcription, we grouped the *Ighv* locus into four domains according to the locations of CTCF binding sites that organize locus contraction and transcript accessibility during V(D)J recombination [(68); Figure 4A]. PCA of normalized *Ighv* segments showed partitioning of duplicate samples into quadrants, but the variance between BM sets was smaller than that of FL samples (Supplementary Figure 4A). By unsupervised Euclidian clustering, we found that the *Ighv* signature of FL FCRL6^+^ pro B cells segregated independently, suggesting that this subset generated a distinct complement of V_H_ segments (Supplementary Figure 4B). FCRL6^+^ cells primarily upregulated distal *Ighv1*/J558 segments from domain 4, but downregulated proximal domain 1 and 2 *Ighv* genes (Figures 4A–C). Indeed, multiple V_H_ segments from these domains were among FL DEGs (Figure 4D). A similar trend of differential locus accessibility was evident in the BM, but did not reach the same threshold of significance (data not shown). Notably, no differences were evident for *Ighv5-2*/V_H_81X or segments encoding PtC-reactive natural Abs, *Ighv12-3* and *Ighv11-2*. Of two phosphorylcholine (PC)-reactive HCs, *Ighv7-1* was downregulated in FCRL6^+^ cells, but *Ighv7-3* was not.

We next examined how *Ighv* accessibility was differentially regulated. First, recombinatorial activity was elevated. FCRL6^+^ pro B cells demonstrated upregulation of both *Rag1* and *Rag2* transcripts by RNA-seq (Figure 4E). Second, non-coding RNAs (ncRNA) critical for *Igh* locus contraction were investigated (69). Among the 14 PAX5-associated intergenic repeat (PAIR) elements that serve as CTCF, PAX5, and E2A binding sites (70), ncRNA transcripts for PAIR4 and PAIR6, which are distally positioned in domain 4, were among upregulated, overlapping DEGs in FCRL6^+^ cells (Figures 3D, 4E). Within the proximal J_H_-Cμ region, Iμ transcripts initiate from the Eμ intronic enhancer (71) and form long-range DNA loops that tether proximal and distal ends of the *Igh* locus via direct binding to both PAIR4 and PAIR6 in a YY1-dependent fashion (72, 73). μ0 transcripts arise just upstream of the most 3′ D_H_ gene (DQ52) (74). RQ-PCR indicated higher Iμ, but lower μ0 transcripts in FCRL6^+^ FL pro B cells (Figure 4F). With respect to accessibility factors, transcripts for *Spi1* (PU.1), *Tcf3* (E2A), *Ikzf1* (Ikaros), *Ikzf3* (Aiolos), *Ctcf* , *Rad21*, and *Ezh2* were downregulated, whereas *Ebf1* was upregulated, but *Pax5* and *Yy1* did not differ (Supplementary Figure 4C). Collectively, these data indicated that FCRL6 expression marked disparate regulation of *Ighv* accessibility that may relate to Eμ-dependent (long-range) vs. independent (local) mechanisms of locus contraction (69, 73). Thus, FCRL6 status indicated heterogeneity of V_H_ gene segment usage among progenitor B cell subsets.

### FCRL6^+^ Cells Harbor Constrained Repertoire Diversity and CDR-H3 Autoreactivity

**Figure 5:**
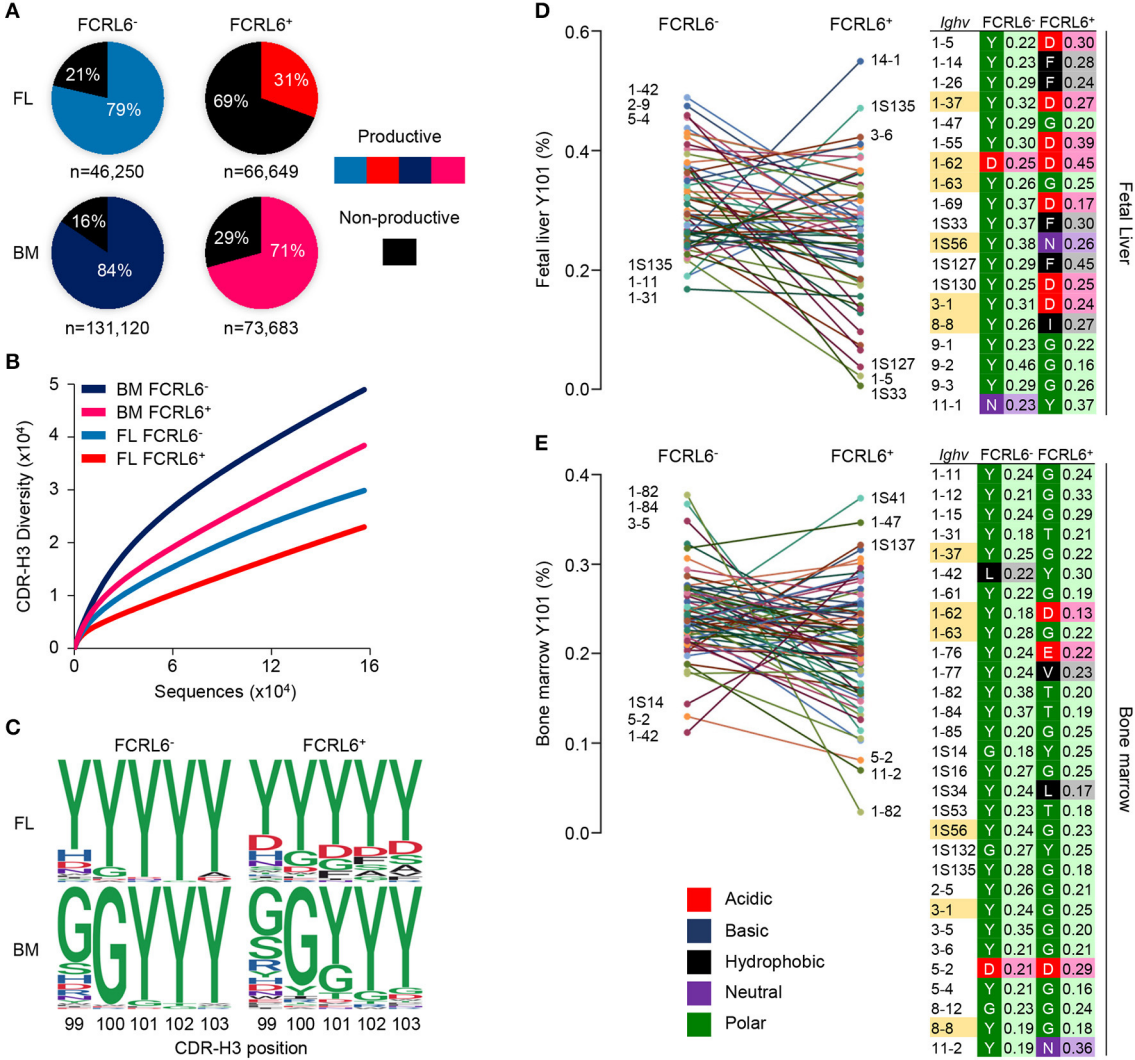
Constrained μHC productivity and repertoire diversity yields hydrophobic and charged CDR-H3s. μHC V(D)J sequences from FCRL6^+^ and FCRL6^−^ pro B cells, sorted from pooled FL E18 or adult BM BALB/c mice by flow cytometry as in Figure 1B, were amplified using 8 V_H_ family consensus and IgM-specific primers, sequenced by Illumina MiSeq, and analyzed with IMGT and R-based software. **(A)** Productivity of total dereplicated unique rearrangements from four FL and BM FCRL6^+^ and FCRL6^−^ pro B cell subsets. Frequencies are indicated within each pie chart and numbers of sequences analyzed per subset are indicated below. **(B)** Rarefaction analysis of CDR-H3 segment diversity defined by Shannon index for total replicated sequences. Numbers of sequences analyzed are listed in Supplementary Figure 5A. **(C)** Logo plots indicating the probability of amino acid usage at positions 99-103 of the CDR-H3 according to PDB numbering of encoded sequences (sequence numbers, Supplementary Figure 6A). Amino acid biochemical characteristics (color coded) are indicated in **(E)**. **(D,E)** Comparisons of tyrosine usage frequency at CDR-H3 position 101 (Y101) for 60 FL **(D)** and 74 BM **(E)** *Ighv* genes with >50 unique productive sequences common between FCRL6^+^ and FCRL6^−^ pro B cell subsets (Supplementary Table 1). The top and bottom three *Ighv* segments are indicated for each subset. A list of *Ighv* genes (right) that predominantly harbor non-Y101 amino acids (FL, *n* = 19 and BM, *n* = 30) are shown according to subset and tissue along with their biochemical characteristics (color-coded) and relative frequencies. *Ighv* segments (*n* = 6) highlighted yellow were common to both tissues.

To investigate *Ighv* sequences from these pro B cells, V(D)J rearrangements were amplified from the four sorted populations. After annotating the amplicons using the IMGT database, we identified 317,702 unique dereplicated sequences that included representation of 8/15 V_H_ families (Supplementary Figures 5A–C). The most striking finding was the low frequency of productive rearrangements among unique dereplicated *Ighv* sequences (both non-productive and productive) generated from FCRL6^+^ vs. FCRL6^−^ pro B cells in the FL (31 vs. 79%) (Figure 5A). In the BM, where Tdt is operative (10), productivity was higher for cells marked by FCRL6, but was still lower than FCRL6^−^ pro B cells. Notably, *Dntt* (Tdt) expression did not differ according to FCRL6 status by RNA-seq. By dividing the number of unique productive sequences from unique dereplicated sequences among the eight amplified V_H_ families, we found that *Ighv1*/J558 was the most prolific in FCRL6^+^ cells (42.6% in FL and 51.1% in BM) (Supplementary Figure 5C). However, relative to all unique dereplicated sequences, the frequency of productive *Ighv1*/J558 rearrangements in FCRL6^+^ cells was very low in FL, but increased in BM (Supplementary Figure 5B, top). Surprisingly, the most productive FL FCRL6^+^ cell rearrangements derived from the small *Ighv11* family (49.5%). However, *Ighv11-2* productivity in the BM was similar for both subsets. *Ighv5-2/*V_H_81X was the most proficient segment among FL FCRL6^+^ cells (70.8%), but was similar in FCRL6^−^ pro B cells (70.3%). However, V_H_81X productivity globally dropped in the BM. Among D and J segments, productivity for FCRL6^+^ cells was higher for D-less joins in the FL and *Ighd1*/DFL16.1 in the BM, whereas *Ighd2*/DSP segments were generally less favored (Supplementary Figures 5D,E). *Ighj1-4* segment usage did not markedly differ by FCRL6 status. The CDR-H3 diversity of rearrangements in FCRL6^+^ cells was relatively lower in both tissues (Figure 5B). This was coincident with generally decreased CDR-H3 length and increased J trimming in the FL (Supplementary Figure 5B). Hence, the generally lower productivity and diversity of V(D)J rearrangements in FCRL6^+^ cells indicated significant mechanistic differences compared to their FCRL6^−^ counterparts.

At the pre-BCR checkpoint, the SLC proteins, λ5 and VpreB, make extensive contacts with the CDR-H3 of the nascent μHC by forming a “sensing site” to survey its biochemical properties (31). Recent work has demonstrated that tyrosine enrichment at position 101 of the CDR-H3, which interacts with three residues in VpreB, favors positive selection and checkpoint passage (32). We thus compared unique productive CDR-H3 sequences from the four subsets by determining amino acid usage at positions 99–103 relative to the cysteine at position 96 (PDB numbering). While variability was evident between tissues, tyrosine enrichment at these five positions was highest for FCRL6^−^ pro B cells (Figure 5C and Supplementary Figure 6A). In contrast, tyrosine content at residue 101 (Y101) was about 1/3 lower for FCRL6^+^ CDR-H3 sequences.

We then inspected the CDR-H3 Y101 frequency by comparing shared *Ighv* segments between FCRL6^+^ and FCRL6^−^ pro B cells with at least 50 productive sequences each (Supplementary Table 1). Marked differences were identified in the range of Y101 usage. Among 60 common FL *Ighv* genes, a comparably narrower spectrum of Y101 composition was evident for FCRL6^−^ pro B cell rearrangements (16.8–48.9%) vs. FCRL6^+^ cells (0.6–54.9%) (Figure 5D). This trend was also apparent for 74 *Ighv* genes shared for the BM (Figure 5E). We then analyzed common sets of *Ighv* genes to assess amino acid predominance at the CDR-H3 101 position. This breakdown identified 19/60 *Ighv* genes in the FL that preferentially utilized non-Y101 amino acids (Figure 5D, right). Surprisingly, while only two FCRL6^−^ *Ighv* sequences exhibited this feature (*Ighv1-62* and *Ighv11-1*), non-Y101 usage was evident for 18 FCRL6^+^ *Ighv* genes. In the BM, 30/74 common *Ighv* genes were predominantly non-Y101 (Figure 5E, right). Although five of these were FCRL6^−^ derived, 27 came from FCRL6^+^ cells. Additionally, six non-Y101 enriched *Ighv* genes were shared between FL and BM FCRL6^+^ cells. These findings indicated that the CDR-H3 features of FCRL6^+^ pro B cells were disproportionately hydrophobic, charged, and less tyrosine enriched. Hence, FCRL6 marked progenitors predisposed to generating rearrangements with altered categories of diversity akin to the characteristics of Abs enriched in innate-like B cells (11).

We next considered *Ighv* sequence data from B-1a cells recently analyzed by the Herzenberg group (12) and CLL clones from different mouse models and strains (Supplementary Figure 6A and Supplementary Tables 2, 3). Among 150 B-1a and 291 CLL *Ighv* sequences, *Ighv1*/J558 segments were the most common and mainly domain 4 derived (Supplementary Figures 6B,C). *Ighv11* was the second most favored among B-1a cells and fourth among CLL clones. CDR-H3 analyses disclosed that, like FCRL6^+^ pro B cells, tyrosine content was generally low and particularly poor at position 101 for both B-1a (42%) and CLL (35%) sequences (Supplementary Figures 6A,D). Collectively, these results demonstrated a progressively lower gradient of Y101 frequencies associated with FCRL6 expression and tissue of origin compared to B-1a and CLL cells (Supplementary Figure 6E). We concluded that the emerging μHC repertoire in FCRL6^+^ pro B cells, which was inefficient, less diverse, and autoreactive, would disfavor pre-BCR formation and positive selection.

### Higher SLC, V_H_11, and Pre-BCR Formation by FCRL6^+^ Progenitors

**Figure 6:**
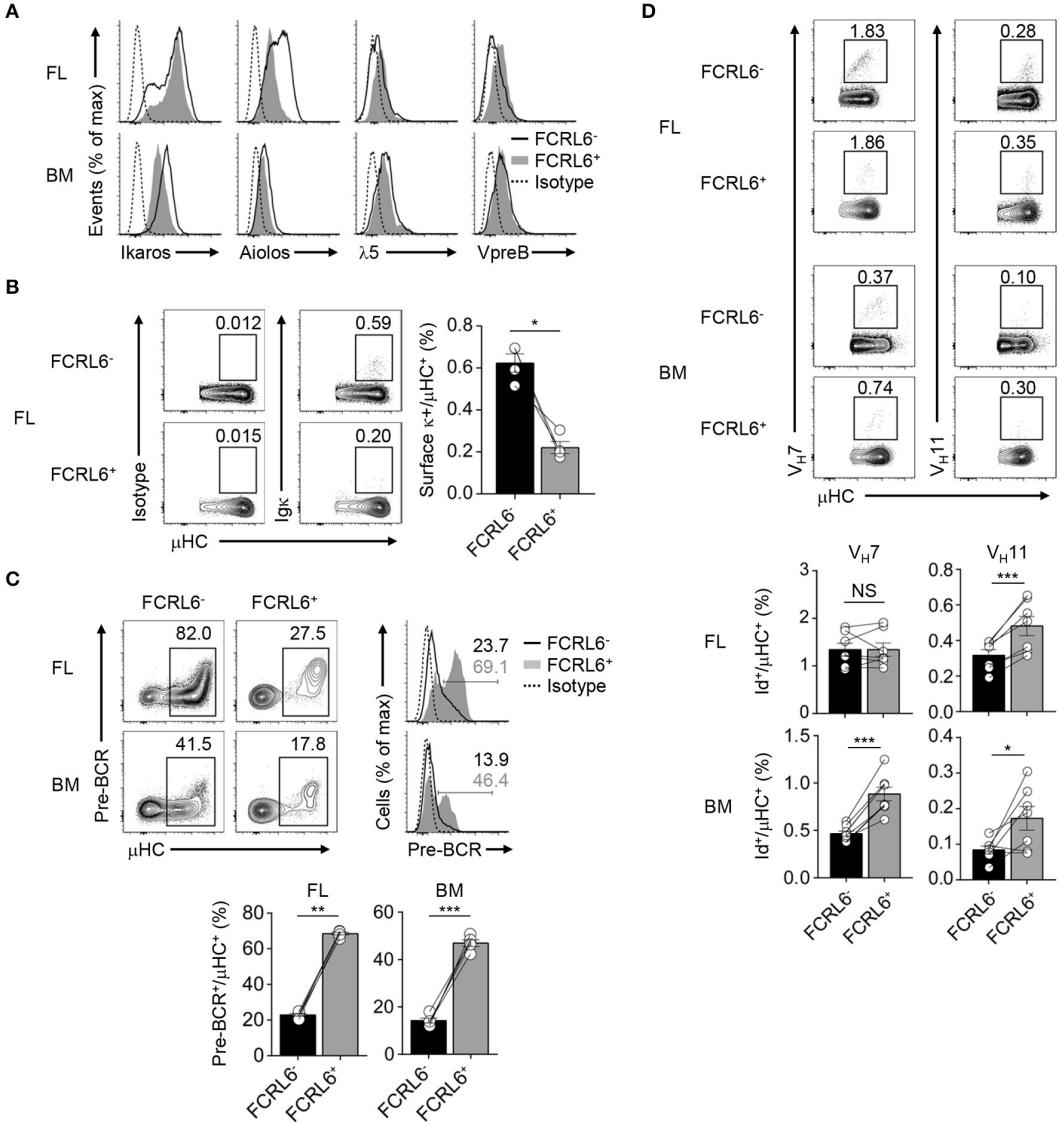
FCRL6 segregates pre-BCR dependent and independent selection and innate-like Ab generation. **(A)** Intracellular and surface staining of transcription factors and SLC components by flow cytometry from FL and BM FCRL6^+^ and FCRL6^−^ pro B cell subsets. **(B)** Flow cytometry analysis of surface κLC expression by μHC^+^ FL pro B cells subsets. Frequencies are indicated adjacent to gated populations. **(C)** Intracellular quantitation of pre-BCR formation by μHC^+^ FL and BM pro B cells. **(D)** Intracellular detection of nascent V_H_7 and V_H_11 within the μHC pool by staining with anti-Id mAbs. Each symbol **(B–D)** represents an individual mouse. Small horizontal lines **(B–D)** indicate s.e.m. NS, not significant; *P < 0.05, **P < 0.01, and ***P < 0.001 as determined by paired Student's *t*-test. Data are from 3 to 5 individual mice and are representative of at least two independent experiments **(A,B)**, three experiments **(C)**, or two (FL) and three (BM) experiments **(D)**.

Given the unconventional CDR-H3 features in FCRL6^+^ pro B cells and the possibility that alternative selection processes may regulate early B-1 cell development (37), we focused on the pre-BCR. By RNA-seq, transcripts for the SLC encoding genes *Igll1* and *VpreB1*, were upregulated in FCRL6^+^ cells, whereas *Ikzf1* and *Ikzf3*, which encode factors (Ikaros and Aiolos) that repress SLC genes (75, 76), were downregulated (Figure 3D and Supplementary Figures 3A, 4C). Intracellular and surface staining validated these relationships, which were variably more pronounced in the FL than BM (Figure 6A). Aiolos also induces exit from the cell cycle to promote LC rearrangement (75, 76). Recent studies proposed that premature LC rearrangement by FL pro B cells may obviate pre-BCR formation, leading to direct BCR generation of potentially autoreactive rearrangements, including V_H_12 (37). Notably, *Igkc* transcripts were among downregulated overlapping DEGs (Figure 3D). Accordingly, the frequencies of surface κLC were 3-fold higher among μHC^+^ FL FCRL6^−^ pro B cells (Figure 6B). This finding indicated differences in the selection of progenitor B cells that correlated with FCRL6 expression.

We then examined pre-BCR formation. By co-staining for μHC, we detected 2 to 3-fold higher pre-BCR levels in FCRL6^+^ compared to FCRL6^−^ pro cells (Figure 6C). Given their unfavorable CDR-H3/Y101 biochemical features, this finding was unexpected. Because *Ighv11* rearrangements were among the most productive in FL FCRL6^+^ cells (Supplementary Figures 5B,C), we employed anti-idiotype (Id) mAbs to detect the fraction of V_H_11 or V_H_7 μHCs that formed BCRs with emerging PtC or PC reactivity. Although V_H_11 detection was generally rare within the μHC pool (Figure 6D), frequencies were indeed higher among FCRL6^+^ vs. FCRL6^−^ cells. V_H_12 staining was extremely rare among progenitors (data not shown). V_H_7 was more readily detected and while it did not markedly differ between FL subsets, its expression by BM FCRL6^+^ pro B cells was comparatively higher. Thus, associated with their disadvantageous CDR-H3 composition, FCRL6^+^ progenitors exhibited higher SLC, V_H_11, and pre-BCR formation, but lower κLC production than their FCRL6^−^ counterparts. These findings suggested the existence of pre-BCR dependent and independent selection processes that varied according to FCRL6 status.

### Myc Induction and V_H_11 B-1a Development Are Pre-BCR-Dependent

**Figure 7:**
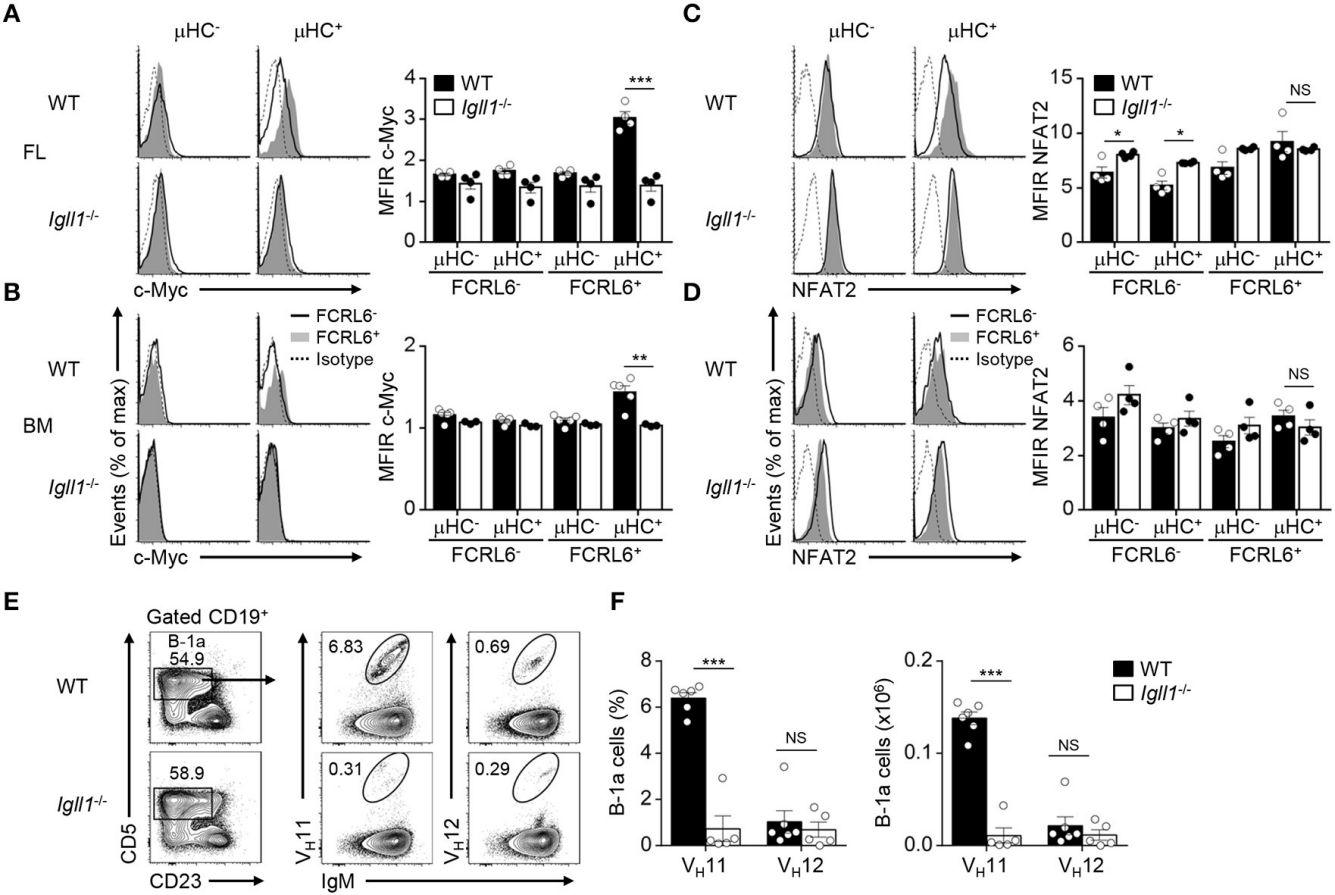
Myc induction by FCRL6^+^ progenitors and V_H_11^+^ B-1a cell development are pre-BCR dependent. Flow cytometry analysis of FL **(A)** and BM **(B)** pro B cells segregated by FCRL6 expression from WT BALB/c and *Igll1*^−/−^ mice intracellularly stained for c-Myc and μHC. **(C,D)** Flow cytometry analysis of pro B cells as in **(A,B)** intracellularly stained for NFAT2 and μHC. Data are shown as MFI ratio (MFIR) (test Ab MFI/control Ab MFI). **(E)** Distribution of V_H_11^+^ and V_H_12^+^ B-1a cells from the PeC of WT and *Igll1*^−/−^ mice by surface staining with anti-Id mAbs. Numbers in the contour plots indicate frequencies of gated populations from representative mice. **(F)** Frequencies and absolute numbers of V_H_11-Id^+^ and V_H_12-Id^+^ B-1a cells. Each symbol represents an individual mouse. Small horizontal lines **(A–D)** indicate s.e.m. NS, not significant; *P < 0.05, **P < 0.01, and ***P < 0.001 as determined by paired **(A–D)** or unpaired Student's *t*-test **(E,F)**. Data are from 3 to 5 mice per strain and are representative of at least two independent experiments **(A–F)**.

Although FCRL6^+^ cells were mitotically repressed (Figures 3C–F), we were surprised to find that *Myc* transcripts were upregulated in FL FCRL6^+^ cells by RNA-seq and RQ-PCR (Supplementary Figure 7A). Myc has important roles in stimulating early B cell development and proliferation at the pro to pre B cell transition (77). Notably, Myc is repressed by Ikaros and Aiolos, promotes both B-1 and B-2 lineage development, and is induced in CLL (15, 76, 77). We confirmed that intracellular Myc expression was elevated in splenic WT B-1a cells and CLL expansions in Eμ-TCL1 Tg mice (Supplementary Figure 7B). We then investigated Myc as a function of μHC status. While Myc was not appreciably induced in μHC^−^ progenitors, it was upregulated by μHC^+^ FCRL6^+^ B-1P and pro B cells (Supplementary Figure 7C). These findings were common to both tissues, but were more robust in the FL. To determine if Myc activation was pre-BCR dependent, we analyzed *Igll1* (λ5)^−/−^ mice. These results confirmed that λ5, and thus pre-BCR assembly, was required for Myc activation by FCRL6^+^ μHC^+^ pro B cells (Figures 7A,B). Myc also amplifies calcium signaling in early B cells to promote NFAT activation (77), which is required for B-1a development and the generation of PtC-specific reactivity (78). NFAT2 was generally higher in FL FCRL6^+^ cells, but levels peaked in FCRL6^+^ μHC^+^ cells (Supplementary Figure 7D). In the BM, NFAT2 levels were globally lower and differences according to FCRL6 were not as pronounced. However, in contrast to Myc, NFAT2 induction in the FL and BM was independent of pre-BCR formation (Figures 7C,D). Finally, we considered the influence of the pre-BCR on B-1a cell V_H_11 repertoire development. In the C57BL/6 strain, V_H_12 usage between WT and *Igll1* (λ5)^−/−^ B-1a cells did not differ (37). We confirmed these findings for V_H_12, but found that V_H_11 reactivity was dramatically lower in both frequency and absolute number in λ5-deficient mice (Figures 7E,F and Supplementary Figure 7E). In summary, these findings indicate that Myc-induction in FCRL6^+^ progenitor B cells and the development of V_H_11^+^ B-1a cells is pre-BCR dependent.

### FCRL6^+^ Progenitors Exhibit Enhanced CXCR5 Migration and TSLP Differentiation

Resemblance of the FCRL6^+^ pro B cell μHC repertoire and transcriptome to innate-like B cells was further underscored by the induction of gene programs encoding components of innate defense, including Toll-like (*Tlr1, Tlr2, Tlr4, Tlr5*), DNA (*Aim2, Ddx58*), and NOD-like (*Nlrc3*) pathogen recognition receptors, as well as cytokine production (*Maf* , *Stat4, Ccl3, Ccl4*) (Figures 3C,D and Supplementary Figures 3A,C). Migration and adhesion programs were also upregulated. DEGs encoding the LFA-1 (*Itgb2*) and VLA-4 (*Itga4, Itgb1*) integrins, which direct migration and adhesion to follicular dendritic (FDC) and endothelial cells (79), were elevated in FL and BM FCRL6^+^ cells (Supplementary Figure 8A). CD69 and *Slamf1*/CD150 were also among overlapping DEGs with higher expression by FCRL6^+^ cells. In contrast, we noted that *Ccr7*, which encodes a chemokine that mediates trafficking to T-cell enriched zones (80), was downregulated in FL FCRL6^+^ cells and elevated on FL FCRL6^−^ pro B cells (Supplementary Figures 3A, 8B). These findings indicated different migration properties for these two FL subsets.

**Figure 8:**
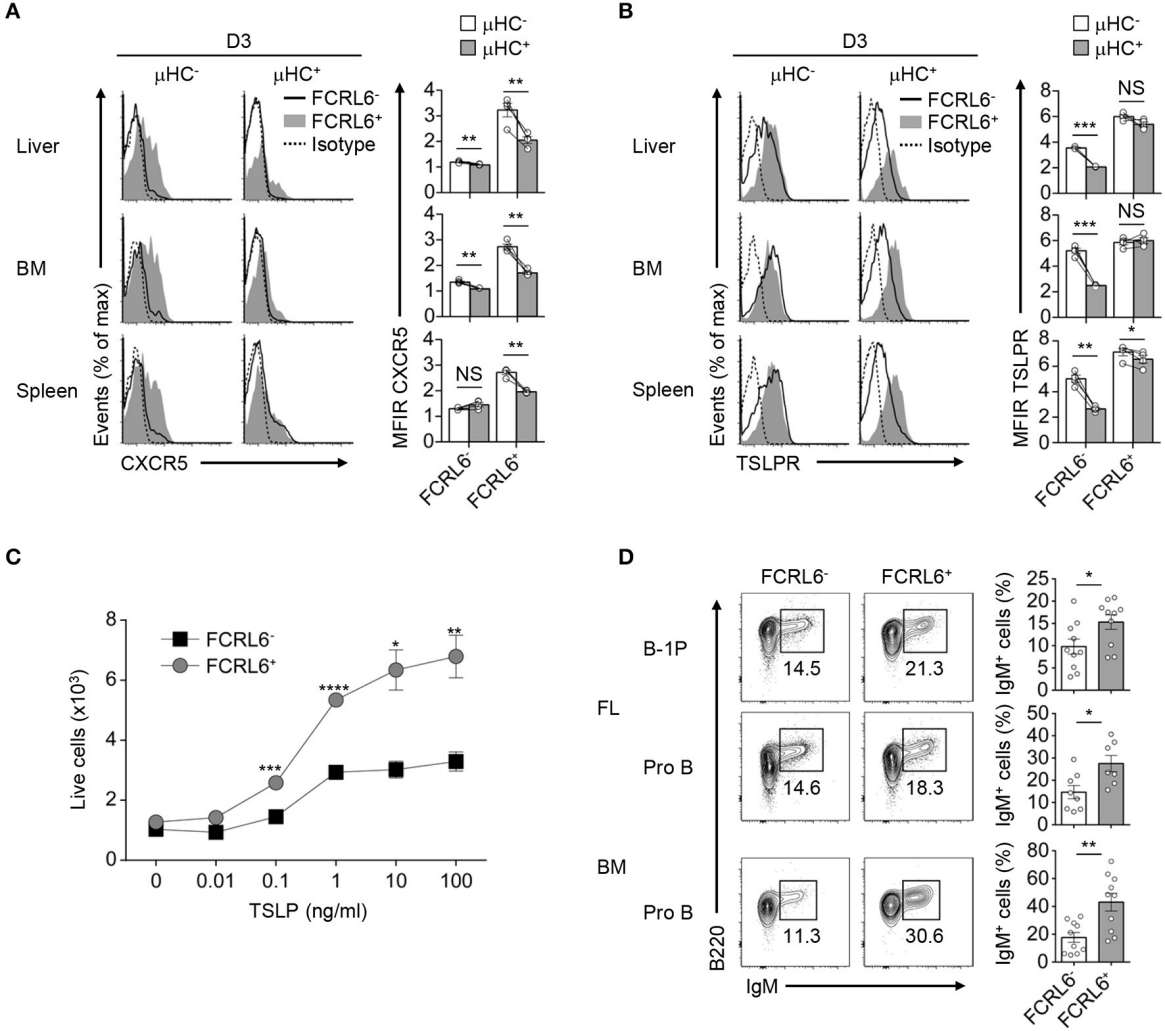
Migration and differentiation of FCRL6^+^ pro B cells is linked to μHC status. Flow cytometry analysis of surface CXCR5 **(A)** or TSLPR **(B)** and intracellular μHC expression in indicated tissues from day 3 (D3) neonatal mice. Data are shown as MFI ratio (MFIR). **(C)** Dose-dependent survival of sorted FCRL6^+^ and FCRL6^−^ BM pro B cells cultured with or without varying concentrations of TSLP (0–100 ng/ml). **(D)** Differentiation of sorted FL and BM FCRL6^+^ and FCRL6^−^ progenitor B cell subsets cultured in transwell plates over 7–8 days with the S17 stromal cell line and cytokines according to (55). Small horizontal lines indicate s.e.m. *P < 0.05, **P < 0.01, ***P < 0.001, and ****P < 0.0001 as determined by paired Student's *t*-test. Data are representative of pooled tissues from one of two independent experiments **(A,B)**, three independent experiments **(C)**, and cumulative data from 5 to 6 individual experiments **(D)**.

B-1a cells express CXCR5 and home to the PeC and body cavities in a CXCL13-dependent fashion (81). Although *Cxcr5* was among upregulated overlapping DEGs, it was not detected on FCRL6^+^ pro B cells (Figure 3D and Supplementary Figure 8B). However, CXCR5^+^ transitional B cells entering the spleen 18 h before birth acquire postnatal CXCL13 responsiveness to initiate white pulp formation (82). Thus, we postulated that CXCR5 might be dynamically induced to direct migration after parturition. We therefore examined CXCR5 in the liver, BM, and spleen from day 3 and 7 neonates. CXCR5 surface density and cell frequency was uniformly higher on FCRL6^+^ progenitors compared to FCRL6^−^ cells among day 3 tissues (Supplementary Figure 8C). Although FCRL6^−^ pro B cells had lower CXCR5, it escalated on subpopulations in the spleen > BM > liver. At day 7, largely homogenous CXCR5 expression was still evident for liver FCRL6^+^ pro B cells, but it bifurcated in the BM and was nearly lost in the spleen. Subpopulations of CXCR5^+^ FCRL6^−^ pro B cells were detectable at day 7 in liver and BM, but a distinct subset emerged in the spleen. Given the impact of pre-BCR formation on Myc induction in FCRL6^+^ cells, we examined CXCR5 as a function of μHC status. At day 3, CXCR5 levels on FCRL6^−^ pro B cells were generally low regardless of μHC expression (Figure 8A). In contrast, elevated CXCR5 expression by FCRL6^+^ μHC^−^ cells markedly declined in μHC^+^ cells. These results indicated that CXCR5 and μHC production were linked in FCRL6^+^ cells, and that migrating μHC^−^ cells repress CXCR5 upon becoming μHC^+^.

We then considered the impact of TSLP, a trophic factor capable of driving differentiation of fetal B cell progenitors to surface IgM^+^ cells (83). FCRL6^−^ pro B cells demonstrated higher IL-7Rα expression, but TSLPR levels did not markedly differ (Figure 2A and data not shown). TSLPR expression by FCRL6^+^ and FCRL6^−^ B cell progenitors was similar among day 3 and 7 postnatal tissues (Supplementary Figure 8D). However, with respect to μHC status, μHC^+^ FCRL6^−^ cells strongly downmodulated the TSLPR (Figure 8B). In contrast, sustained TSLPR expression by μHC^+^ FCRL6^+^ cells indicated that responses to this cytokine differed between these subpopulations. To investigate this, we first sorted BM FCRL6^+^ and FCRL6^−^ pro B cells and performed *in vitro* growth assays in the presence of TSLP. Survival of both subsets increased in a dose-dependent manner; however, FCRL6^+^ pro B cells exhibited greater TSLP responsivity (Figure 8C). We next examined differentiation by adopting an *in vitro* co-culture system established by the Dorshkind group that includes TSLP (19). In initial studies, we detected few IgM^+^ B cells at day 4 and by day 12, most cells were dead (data not shown). At day 8, however, sorted FCRL6^+^ FL B-1P and FL and BM pro B cells were consistently more proficient than FCRL6^−^ cells in differentiating to IgM^+^ cells (Figure 8D). Thus, FCRL6^+^ progenitors exhibited preferential CXCR5- and TSLPR-mediated homing and differentiation potential.

## Discussion

Here FCRL6 distinguished a subset of B cell progenitors that is evident from embryogenesis through adulthood and thus appears to be conserved throughout ontogeny. FCRL6 expression delineated two pro B cell subpopulations with marked differences in their molecular signatures, biology, and emerging μHC repertoires. Their distinct rearrangement activities, migration potential, and differentiation pathways culminated in pre-BCR dependent and independent modes of selection. This disparity further correlated with the developmental potential of two B-1a stereotypic clones that give rise to natural Abs.

FCRL6^+^ FL B cells are smaller, almost uniformly c-Kit^+^, exhibit less BP-1 and IL-7Rα expression, but have higher levels of SLC and pre-BCR formation despite lower μHC production. Thus, FCRL6 primarily marks a subset of progenitors harboring features of Hardy Fr B and C cells or late pro B/pre B-I stages according to the Basel nomenclature (56, 84). Consequently, c-Kit is a distinguishing marker of B-1 progenitors (85). Although the ImmGen database indicates that *Fcrl6* transcripts are upregulated by CD43^−^ Fr D FL cells, FCRL6 was largely absent from this population in our analysis. This discrepancy could be technical and/or due to differences in transcript vs. protein regulation. Furthermore, by RNA-seq analysis, *Dntt* (Tdt), *Cd24a* (CD24/HSA), and *Il2ra* (CD25) were not among DEGs, but *Rag1/2* transcripts were significantly higher in FCRL6^+^ cells. By comparison, the FCRL6^−^ progenitor pool includes pro B as well as some cells of larger size akin to pre B or Fr C′ cells. It is possible that this latter subpopulation could account for findings of relatively less cell division and proliferation among FCRL6^+^ cells. However, the collective disparity in SLC expression, pre-BCR formation, innate-like gene transcription, V_H_ family usage, repertoire diversity, and Myc induction, as well as migration and differentiation potential, indicate that FCRL6 expression segregates biologically different B cell progenitors.

An analysis of two common laboratory mouse strains revealed greater frequencies of FCRL6^+^ pro B cells in the BM of WT BALB/c compared to C57BL/6 mice. A recent study by Fransen et al. showed a relative increase in B-1 B cells in the BALB/c vs. C57BL/6 strain (86). This finding correlated with IgA abundance and polyreactivity, microbiota diversity, and host defense responses to Salmonella. How and if a difference in the quantity of FCRL6^+^ pro B cells between these strains differentially impacts B cell development is not yet known. However, it suggests that there may be a link between FCRL6 progenitor frequencies and B-1 abundance. Given that FCRL6^+^ progenitors make up ~11% of the FL and ~1–2% of the BM CD19^+^ cells, this relationship may also extend to tissue-based differences in B-1 output.

Significant progress has been made in identifying B-1 progenitors (19) and regulatory factors that drive the development of these cells (28, 29). Studies of the Let7-Lin28b-Arid3a axis have provided supportive evidence for a developmental switch (5, 25–27). However, *in vitro* provision of these elements did not recapitulate expected biases of natural Ab repertoires enriched with the PtC-reactive V_H_11 and V_H_12 families (26). Thus, while the Let7 developmental circuit may differentially impact features of B-1 vs. B-2 differentiation that predominate in fetal and adult life (i.e., phenotype, tissue localization), some programmatic regulation (i.e., repertoire formation and selection) may be independent of this governance. Accordingly, these FCRL6-related studies, which disclose a progenitor subpopulation possessing characteristics favorable for natural Ab formation, may provide new insight into the mechanisms responsible for ensuring the generation of these stereotypic Abs.

Beyond their different transcription signatures and repertoire characteristics, these findings imply that the disparate expression of certain regulatory factors by FCRL6^+^ progenitors may dictate differences in their development. This is further highlighted by: (i) the increased efficiency of V_H_11 μHC generation and pre-BCR formation by FCRL6^+^ cells, and (ii) the marked loss of V_H_11, but not V_H_12 B-1a development in *Igll1*^−/−^ mice. Hence, these data indicate that differences in Ab generation and selection maybe hard wired. The regulatory factors responsible may relate to nervous system-related DEGs identified by the GO pathway analysis. *Tcf7*/*Lef1* genes, which regulate Wnt/β-catenin signaling and self-renewal, also have roles in early B cell development and could be attractive candidates (87, 88). *Bcl11a, Foxo1*, and *Klf3* were also among DEGs, but how these factors influence *Igh* repertoire formation with respect to the newfound heterogeneity disclosed here, remains unclear.

The B-1a BCR repertoire is understood to be driven by Ag-dependent positive selection (89, 90). Here, we identified pro B cell subset-specific *Igh* repertoire skewing that became evident from the onset of recombination through pre-BCR selection. Despite elevated *Rag1*/*2* expression and cell cycle enrichment in G0/G1, DNA repair was globally repressed in FCRL6^+^ progenitors. Furthermore, clear differences in proximal vs. distal *Ighv* locus accessibility, V(D)J productivity, CDR-H3 composition, diversity, and μHC expression, illustrated their developmental divergence. These findings imply that FCRL6^+^ cells may serve an ancient, but fundamental role as specialized generators of constrained Ig rearrangements that contribute to the formation of natural Abs. For example, V_H_11 sequences derived from B-1a cells lack N-addition and thus likely originate from a progenitor that does not express Tdt. However, the capacity to rearrange this V_H_ family may not be exclusive to cells marked by FCRL6, as we detected V_H_11-Id^+^ μHC in FCRL6^−^ progenitors. Evidently, mechanistic restrictions inherent to FCRL6^+^ cells serve to restrain Ab diversification by sculpting rearrangements that encode charged or hydrophobic CDR-H3, features that are enriched in innate-like MZ and B-1a B cells (11). Because FCRL6 expression is lost following positive checkpoint selection, we did not examine μHC sequences after this point in differentiation. However, to investigate their developmental potential, we performed transfer studies with sorted FL populations that indicated both FCRL6^+^ and FCRL6^−^ pro B cells can give rise to B-1 cells (data not shown). One complicating factor in these experiments is the current inability to specifically trace progenitors in terms of their history of *Fcrl6* expression. Secondly, the direct transfer of FL B cell progenitors into adult immunodeficient mice does not recapitulate the dynamic microenvironmental interactions these cells experience during ontogeny. Thus, distinguishing FCRL6^+^ from FCRL6^−^ progenitors following this development stage and dissecting their fate will require lineage tracing using genetically engineered models.

These studies also highlight the developmental heterogeneity of the B-1 repertoire by showing a requirement for SLC, and specifically λ5, in V_H_11^+^ B-1a cell differentiation. In contrast, we and others (37) found that V_H_12^+^ B-1a cells were not impacted in *Igll1*^−/−^ mice. More than 20 years ago, Wasserman et al. established that paring between SLC and V_H_11-μHCs was weak, but higher for λ5 than VpreB (33). This finding stimulated a mechanistic hypothesis that selection mechanisms at the pre-BCR stage of development may differ in fetal vs. adult life. Yoshikawa et al. also found generally diminished binding for V_H_11-μHCs with SLC that was higher for J_H_1 containing rearrangements (34). However, their studies demonstrated that pre-BCR assembly was indeed required for positive selection of V_H_11^+^ B cells. One feature that may impair efficient interactions with SLC, and was evident for the FCRL6^+^ progenitor CDR-H3 repertoire generally, is a deficiency of Y101 among V_H_11 sequences. Thus, several mechanisms appear to impact selection at this stage of early B cell development that differ according to V_H_ family as well as ontogenetic stage. Recent studies by the Barton group have demonstrated that certain TLRs influence B-1a cell heterogeneity by shaping the Ab repertoire and promoting a division of labor between cells that harbor reactivity to self (e.g., V_H_11) vs. microbiota-derived antigens (91).

The increased efficiency of pre-BCR formation, Myc induction, and consequences of μHC expression (e.g., migration, differentiation), indicate positive selection and differentiation of FCRL6^+^ cells along a distinct developmental path. However, the promotion of μHCs harboring CDR-H3s with disadvantageous composition through the pre-BCR checkpoint, contrasts with the notion that such autoreactive features trigger negative selection to maintain central tolerance (30). This unexpected result, in light of the importance of the Y101 residue (31, 32), might suggest that non-CDR-H3 interactions contribute. It is intriguing that autonomous BCR signaling in CLL involves an FR2 epitope (92). Perhaps, nascent μHC/SLC interactions of low affinity are compensated by altered stoichiometry. Ikaros and Aiolos repression correlated with VpreB/λ5 upregulation in FCRL6^+^ cells. Emerging CDR-H3s with unfavorable H-bond potential could become saturated by excess SLC that precipitates pre-BCR formation. Evidence that premature κLC rearrangement obviates this checkpoint (37), was also verified here, but appears to differ according to FCRL6 expression. Thus, at least two pathways may promote the development of μHC^+^ innate-like progenitors. Furthermore, these modes of pre-BCR dependent and independent passage correlate with V_H_11 and V_H_12 B-1a development and Myc induction.

Progenitors marked by FCRL6 exhibited a strong transcriptomic, phenotypic, and *Ighv* repertoire resemblance to CLL cells. In addition to their emerging autoreactivity, CLL-related transcription factors (LEF-1, NFAT2), innate-defense components (TLRs), chemokines/cytokines (Ccl3, Ccl4), migration elements (CXCR5), and Myc expression were also shared. Hayakawa found that both BCR autoreactivity and B-1a physiology were required for CLL leukemogenesis in mouse models (15). Our data demonstrate that FCRL6^+^ progenitors exhibit both these properties and have the potential to home to sites where chronic stimulation and innate inflammation could fuel their transformation over time.

The interplay between developing B cell progenitors and the microenvironment into which they are born and ultimately home to during ontogeny is dynamic and physiologically complex. Based on the marked shift in biology triggered by μHC expression and pre-BCR assembly on Myc, CXCR5, and TSLPR, it appears that perinatal FCRL6^+^ cells are poised for migration even prior to μHC expression. These findings are in line with those by Wen et al. who demonstrated the association of CXCR5^hi^-expressing V_H_11^+^ B-1a cells with white pulp FDCs in the neonatal spleen (93). In this fetal microenvironment, FCRL6^+^ cells could contribute to both architectural changes of developing secondary lymphoid tissues and innate-like humoral defense prior to parturition.

These results raise the question of whether progenitors marked by FCRL6 in the FL as well as the BM represent and/or derive from a shared developmental pathway. While there are many compartment-specific differences, we favor the hypothesis that FCRL6 distinguishes a progenitor B cell subset with distinct mechanistic properties that is conserved during early B lymphopoiesis throughout ontogeny. This postulate is based on data resulting from several different experimental approaches including 265 overlapping DEGs identified by RNA-seq, cell cycle G0/G1 phase enrichment, constrained diversity of *Ighv* sequences, and preferential TSLP-mediated differentiation. One limitation for making direct comparisons between FL and BM B cell progenitors is the disparity in surface expression of certain differentiation markers such as those employed by the Hardy scheme (e.g., CD43). However, the development of an FCRL6-specific mAb enabled direct isolation of this minority subset from both tissues. A major difference between these compartments is the expression of Tdt in the BM. Despite the global impact of this enzyme on promoting productivity and repertoire diversity, similar constrained features were found for BM FCRL6^+^ *Ighv* sequences relative to FCRL6^−^ progenitors. Lower productivity and diversity, as well as predominant CDR-H3 non-Y101 usage, was evident for FCRL6^+^ compared to FCRL6^−^ progenitor sequences regardless of their tissue of origin. Indeed, the induction of Myc and NFAT2 expression in BM FCRL6^+^ cells was less robust than their FL counterparts. However, these findings were consistent for B-1P and pro B cells, and in the case of Myc, validated by signal attenuation in *Igll1*(λ5)^−/−^ mice. The blunted effects observed in BM FCRL6^+^ cells may relate to microenvironmental differences or the diversity imposed by Tdt. SLC as well as Ikaros and Aiolos expression were also relatively lower in the BM. Future work will be required to address the regulation and fate of BM-derived FCRL6^+^ progenitors.

The function of FCRL6, its ligand(s), and regulatory influence as a defining marker is unknown. Ig binding studies with transductants and by surface plasmon resonance did not reveal evidence of Ab binding (data not shown). The lack of a canonical tyrosine-based cytoplasmic motif suggests that FCRL6 likely recruits different intracellular effectors than other FCRL family members. Whether FCRL6 directly influences SLC interactions with non-Y101 containing μHC rearrangements or pre-BCR formation via its ectodomain and/or through intracellular signaling is currently unknown. Thus, it is not yet clear whether FCRL6 serves an active regulatory role in modulating progenitor development or is simply a marker for this cell subset. A hurdle in modeling its role is a lack of cell lines that express it and thus *in vivo* systems will be required to define its physiological functions.

In summary, we have taken an unbiased approach to investigating a distinguishing marker of early B lineage differentiation. This work has yielded important insight into the developmental heterogeneity, origins, and selection of innate-like B cells. The frequencies of FCRL6^+^ cells reflect the B-1 potential of fetal and adult tissues and their biased polyreactive repertoires, genetic programs, and migratory features are shared with MZ, B-1a, and CLL cells. These findings introduce evidence for pre-BCR dependent and independent pathways of B-1 development and provide new insight into mechanisms governing selection from the earliest stages of innate-like B cell differentiation.

## Data Availability Statement

RNA-seq transcriptome data have been deposited in GEO under the accession code: GSE132438. *Igh* sequences have been deposited in the NCBI BioSample database under SRA accession: PRJNA547609.

## Ethics Statement

This animal study was reviewed and approved by the UAB Institutional Animal Care and Use Committee (IACUC). UAB IACUC approval protocols IACUC-10208, IACUC-20627, and IACUC-7947.

## Author Contributions

KH and W-JW performed the majority of experiments and wrote the manuscript. RD, RS, and RL assisted with the generation and characterization of anti-FCRL6 mAbs. JE and MS performed RNA isolation and RQ-PCR analysis. DC and LI analyzed and interpreted the RNA-seq data. RK, MK, MS, and AV generated *Ighv* sequences, analyzed the data, and provided scientific input. RD conceived the project, directed research, analyzed data, and wrote the manuscript.

### Conflict of Interest

The authors declare that the research was conducted in the absence of any commercial or financial relationships that could be construed as a potential conflict of interest.
