# Supplementary material for: Fc Receptor-Like 6 (FCRL6) Discloses Progenitor B Cell Heterogeneity That Correlates With Pre-BCR Dependent and Independent Pathways of Natural Antibody Selection
Source: Front Immunol. 2020 Feb 14;11:82. doi: 10.3389/fimmu.2020.00082 (PMC7033751; doi:10.3389/fimmu.2020.00082)
Supplement: Supplementary file 1 [file Presentation_1.pdf]

Supplementary Figure 1

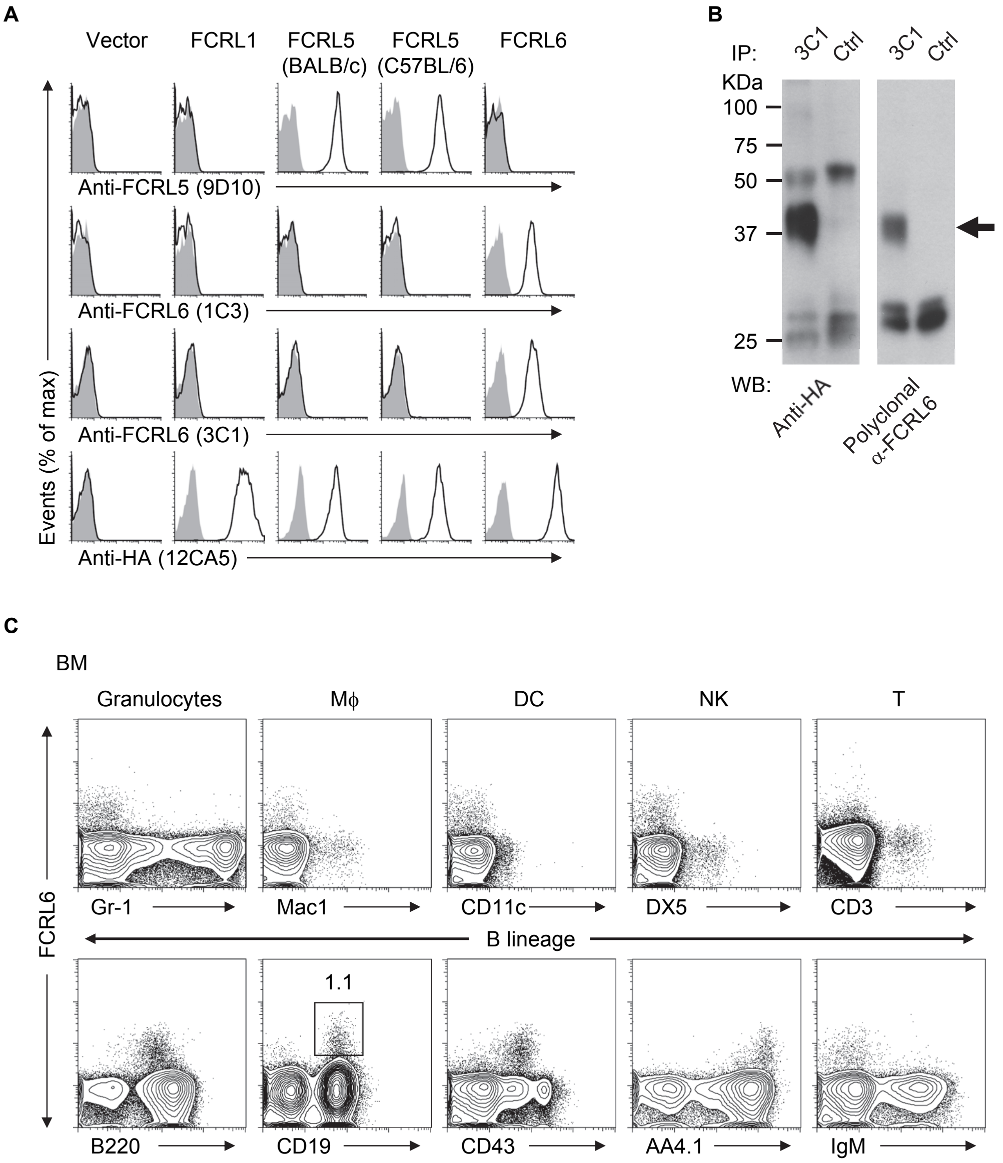

Supplementary Figure 1. Specificity of FCRL6 monoclonal antibodies (mAb) and early B cell restricted expression. (**A**) Specificity of rat anti-mouse FCRL6 mAbs. *E. coli*-derived recombinant protein of the two FCRL6 Ig-like extracellular domains was injected into Fisher rats and mAbs were generated by standard techniques as previously (**Won et al., 2006**). Specificities of the 1C3 and 3C1 subclones were tested for cross-reactivity with a panel of BW5147 retroviral transductants expressing HA-tagged mouse FCRLs by staining with the indicated mAbs prior to flow cytometry analysis (black lines) or an isotype-matched control (gray shade). (**B**) Molecular nature of FCRL6 (indicated by the arrow). BW5147 FCRL6 transductants were immunoprecipitated with the 3C1 mAb or an isotype control (rat IgG2aκ) and blotted with an anti-HA (12CA5) mAb or rabbit anti-FCRL6 polyclonal Abs. (**C**) Analysis of FCRL6 expression by bone marrow (BM) cells from adult BALB/c mice co-stained with the indicated myeloid, lymphoid, and B lineage differentiation markers and anti-FCRL6 (1C3). Number adjacent to the gate indicates the percentage of $CD19^{+}FCRL6^{+}$ lymphocytes. Macrophage (Mϕ), dendritic cells (DC), and natural killer (NK) cells.

Supplementary Figure 2

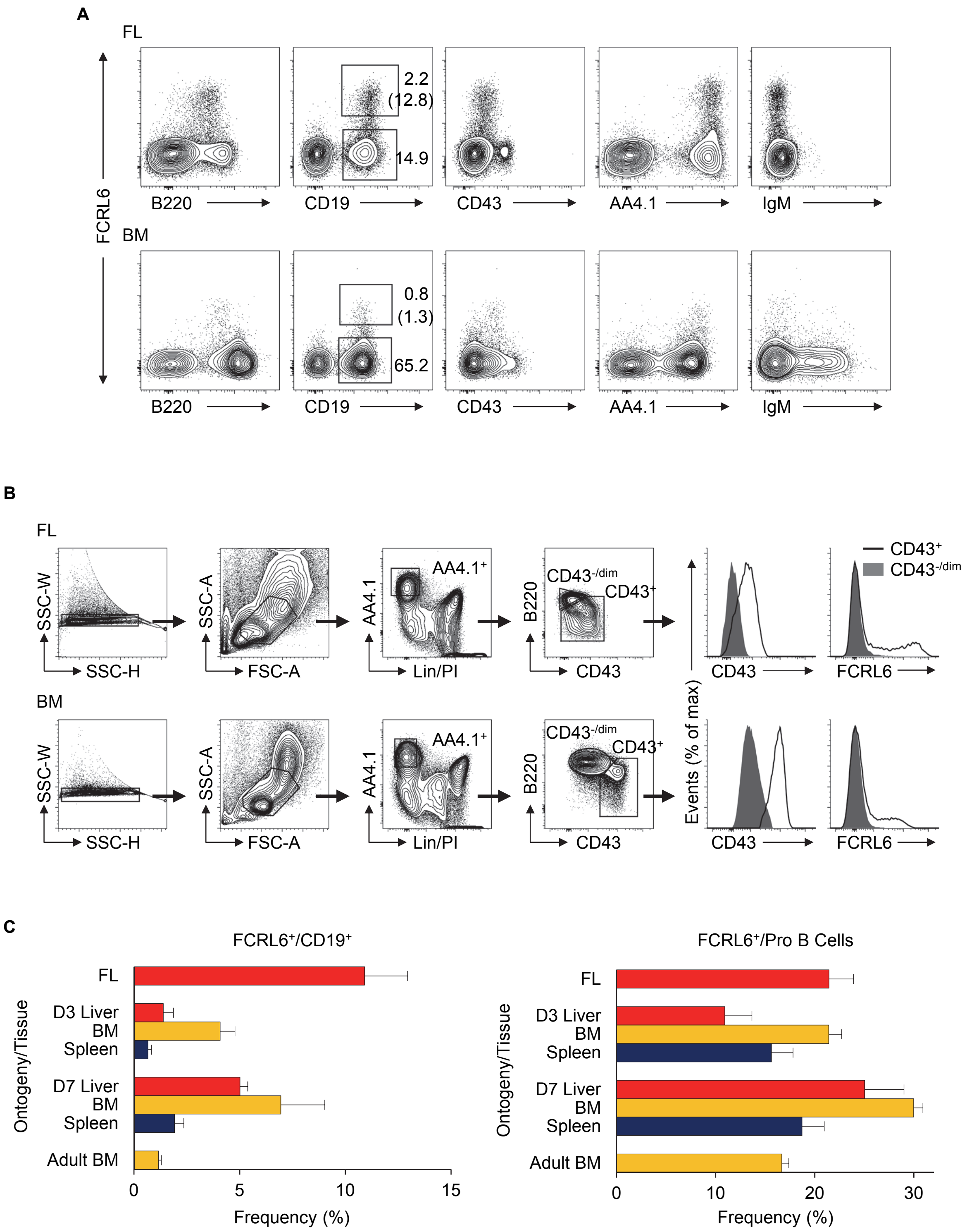

Supplementary Figure 2. FCRL6 is expressed by B cell progenitors throughout ontogeny. (**A**) Analysis of FCRL6 expression by E18 fetal liver (FL) and adult BM from BALB/c mice co-stained with B lineage differentiation markers and anti-FCRL6 (1C3). Frequencies are indicated adjacent to the gates. The numbers in parentheses indicate the percentage of $FCRL6^{+}$/$CD19^{+}$ lymphocytes. (**B**) Analysis of FCRL6 expression by FL and BM B cells according to CD43 status. (**C**) Frequencies of $FCRL6^{+}$ cells among total B (gated as in **A** above) and pro B cells (gated as in **Figure 1B**) determined by staining E18 FL (*n* = 6), adult BM (*n* = 6), and indicated tissues from 3 (*n* = 7) or 7 (*n* = 4) day old neonates. Small horizontal lines indicate s.e.m.

Supplementary Figure 3

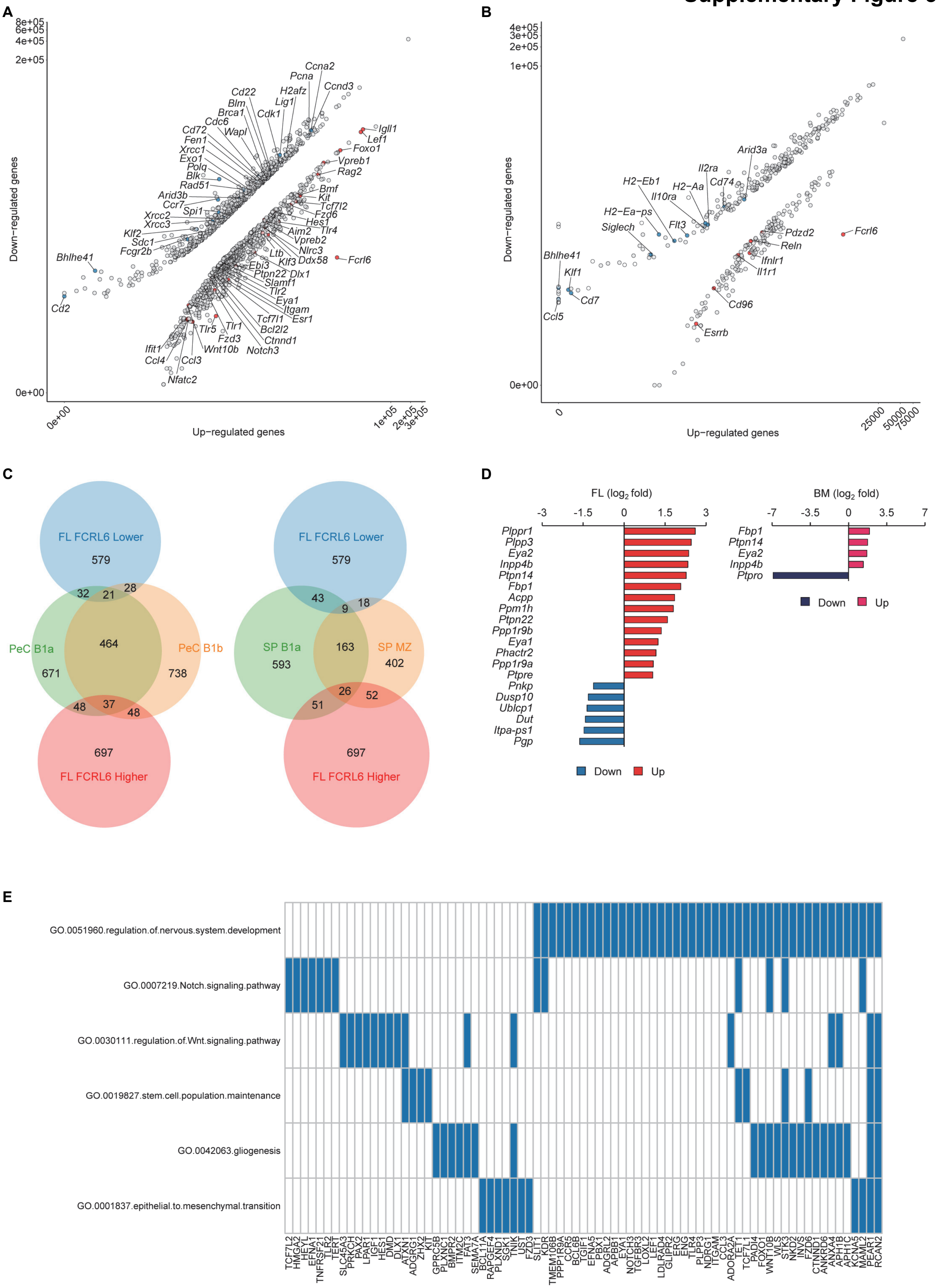

Supplementary Figure 3. Biologic discrimination of FCRL6$^+$ FL and BM pro B cell gene expression profiles. (**A**-**B**) Scatter plots showing differential expression of genes by FL (**A**) and BM (**B**) pro B cells according to up or downregulation of FCRL6 expression. (**C**) Scaled Venn diagrams (https://www.stefanjol.nl/venny) showing gene overlap between up or downregulated DEGs from FCRL6$^+$ and FCRL6$^-$ FL pro B cells (from **Figure 3B**) and peritoneal cavity (PeC) B-1a and B-1b or spleen (SP) B-1a and marginal zone (MZ) B cell subsets extracted from the Immgen database (https://www.immgen.org). Upregulated genes (>1.4 fold) from the indicated innate-like B cell subsets were determined relative to follicular B cells from respective tissues using the Immgen population comparison database. (**D**) DEGs encoding phosphatases by FCRL6$^+$ FL and BM pro B cells. (**E**) Correlation matrix detailing FL DEGs upregulated by the regulation of nervous system development GO pathway (GO:0051960) that are shared with related pathways.

Supplementary Figure 4

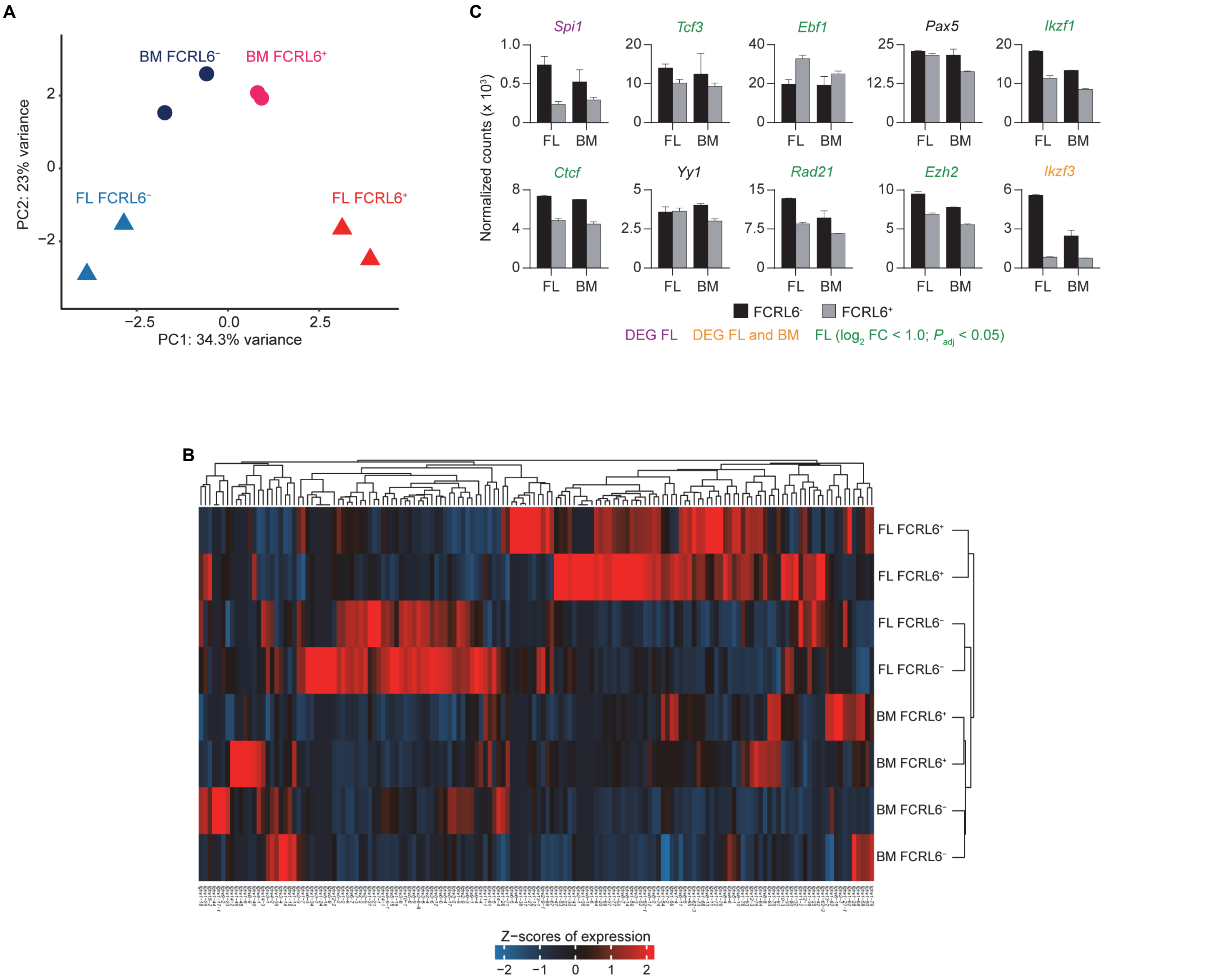

Supplementary Figure 4. Analysis of *Ighv* repertoire and transcription/ accessibility factor gene expression by pro B cell subsets. (**A**) PCA plot of *Ighv* segments expressed by the four pro B cell subsets from FL and BM according to FCRL6 status determined by comparing normalized counts by RNA-seq analysis in duplicate. (**B**) Heat map of *Ighv* segments determined by unsupervised Euclidian clustering. (**C**) Normalized transcript expression of selected transcription and regulatory factors that modulate locus accessibility. Small horizontal lines indicate s.e.m. Note genes labeled in green are significant in FL ($P_{adj} < 0.05$), but did not reach the DEG threshold (one-fold change in $\log_2$ value).

Supplementary Figure 5

A

Subset	Total replicates	Unique dereplicated	Unique productive	Unique productive (%)	Clonotypes
FL FCRL6$^-$	62,764	46,250	36,358	0.79	11,521
FL FCRL6$^+$	114,212	66,649	20,386	0.31	11,255
BM FCRL6$^-$	176,410	131,120	110,731	0.84	42,148
BM FCRL6$^+$	117,692	73,683	52,148	0.71	10,455
Total	471,078	317,702	219,623		75,379

B

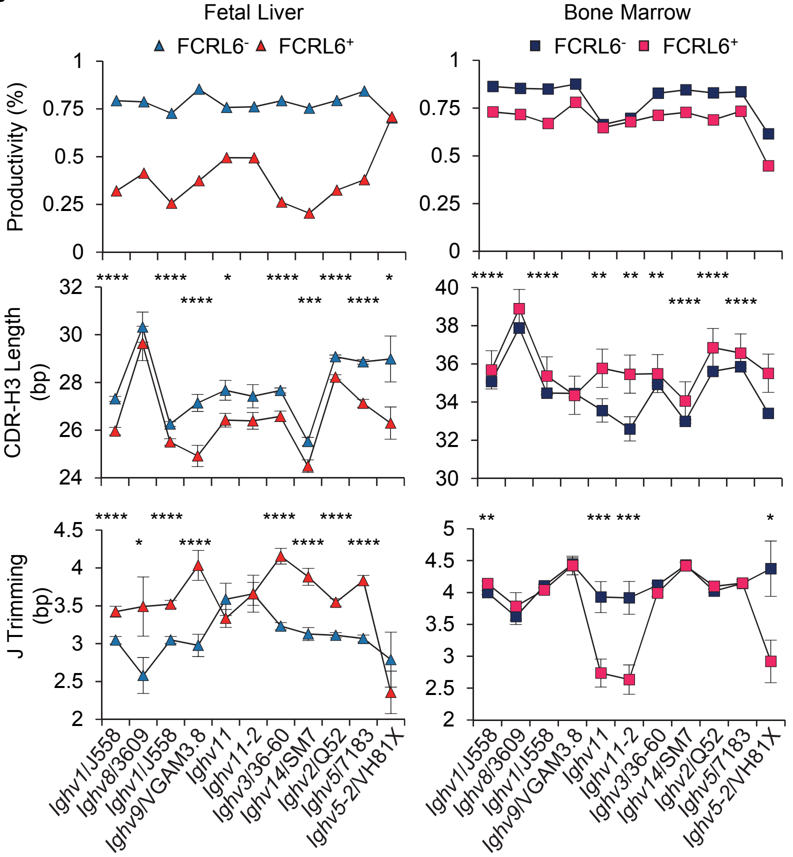

C

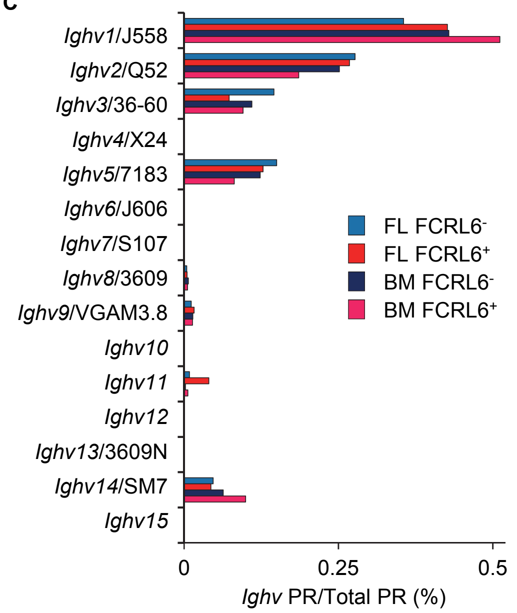

D

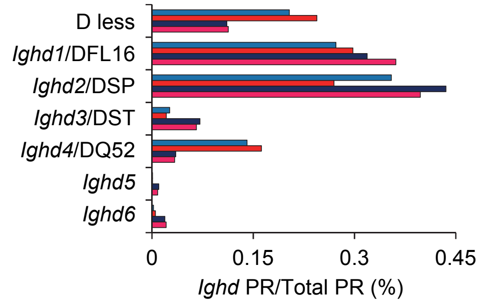

E

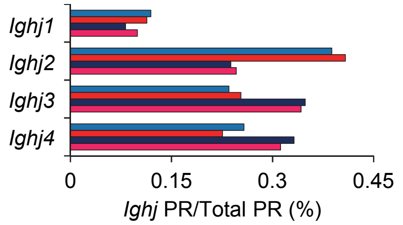

Supplementary Figure 5. *Ighv* repertoire analysis of FL and BM pro B cells according to FCRL6 status. (**A**) Summary of sequence numbers and characteristics from the four subsets quantitated by replication status and productivity. Clonotypes were defined by identical VJ and CDR-H3 length and ≥90% bp identity. No *Ighv7* sequences were amplified and for *Ighv4*, *6*, *10*, *12*, *13*, and *15,* <100 total dereplicated sequences were identified. (**B**) Productivity, CDR-H3, and J trimming features of unique dereplicated productive sequences of eight *Ighv* families and notable segments (*11-2* and *5-2*) according to locus position. The *Ighv1*/J558 family in (**B**) was segregated according the location of segments within the *Ighv* locus that derive from domains 3 or 4 as in **Figure 4A**. Small horizontal lines indicate s.e.m. **P* < 0.05, ***P* < 0.01, ****P* < 0.001 and *****P* < 0.0001 as determined by paired Student's *t*-test. (**C**-**E**) Frequencies of productive *Ighv* (**C**), *Ighd* (**D**), and *Ighj* (**E**) genes relative to total productive reads (PR) among the four pro B cell subsets.

Supplementary Figure 6

A

	99		100		101		102		103		Sequences
FL FCRL6⁻	Y	0.73	Y	0.87	Y	0.96	Y	0.99	Y	0.92	35920
BM FCRL6⁻	G	0.62	G	0.96	Y	0.93	Y	0.95	Y	0.95	94419
FL FCRL6⁺	Y	0.61	Y	0.74	Y	0.69	Y	0.71	Y	0.66	19870
BM FCRL6⁺	G	0.41	G	0.81	Y	0.64	Y	0.85	Y	0.82	45361
B-1a	Y	0.23	Y	0.32	Y	0.42	Y	0.41	Y	0.25	150
All CLL	D	0.24	Y	0.33	Y	0.35	G	0.37	Y	0.45	258
CLL Y101-	D	0.26	Y	0.26	D	0.21	G	0.28	Y	0.57	172
CLL Y101+	D	0.21	Y	0.49	Y	1.00	G	0.53	S	0.42	86

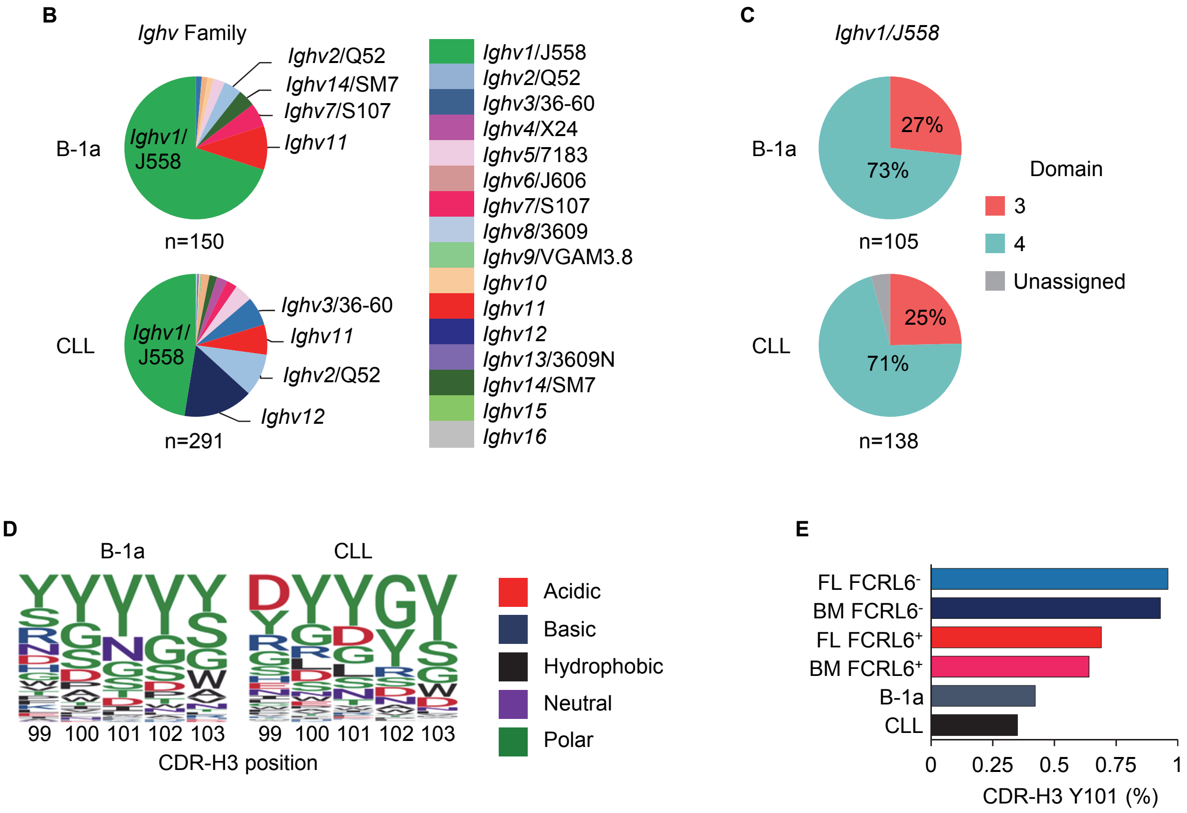

Supplementary Figure 6. Analysis of *Ighv* usage and CDR-H3 composition from FCRL6^{+} and FCRL6^{-} FL and BM pro B cells, B-1a, and CLL *Igh* sequences. (**A**) Summary of amino acid usage frequency by CDR-H3 position for the four pro B cell subsets, B-1a cells (**Yang et al., 2015 – Supplementary Table 2**), and CLL expansions (total, Y101 or non-Y101) from published sequences and unpublished CLL sequences (**Supplementary Table 3**). The total numbers of encoded CDR-H3 sequences analyzed for each subpopulation are indicated in the right column. (**B**) *Ighv* usage for B-1a and CLL sequences by family. The numbers of sequences analyzed are shown below the pie charts. (**C**) *Ighv1*/J558 family usage from B-1a and CLL sequences segregated by *Ighv* locus domain. (**D**) Logo plots detailing the probability of amino acid usage by CDR-H3 amino acid position for B-1a and CLL sequences. (**E**) Relative frequency of Y101 among productive unique sequences (enumerated in **A**) from the four FCRL6^{+} and FCRL6^{-} pro B cell subsets, as well as B-1a cells, and CLL expansions.

Supplementary Figure 7

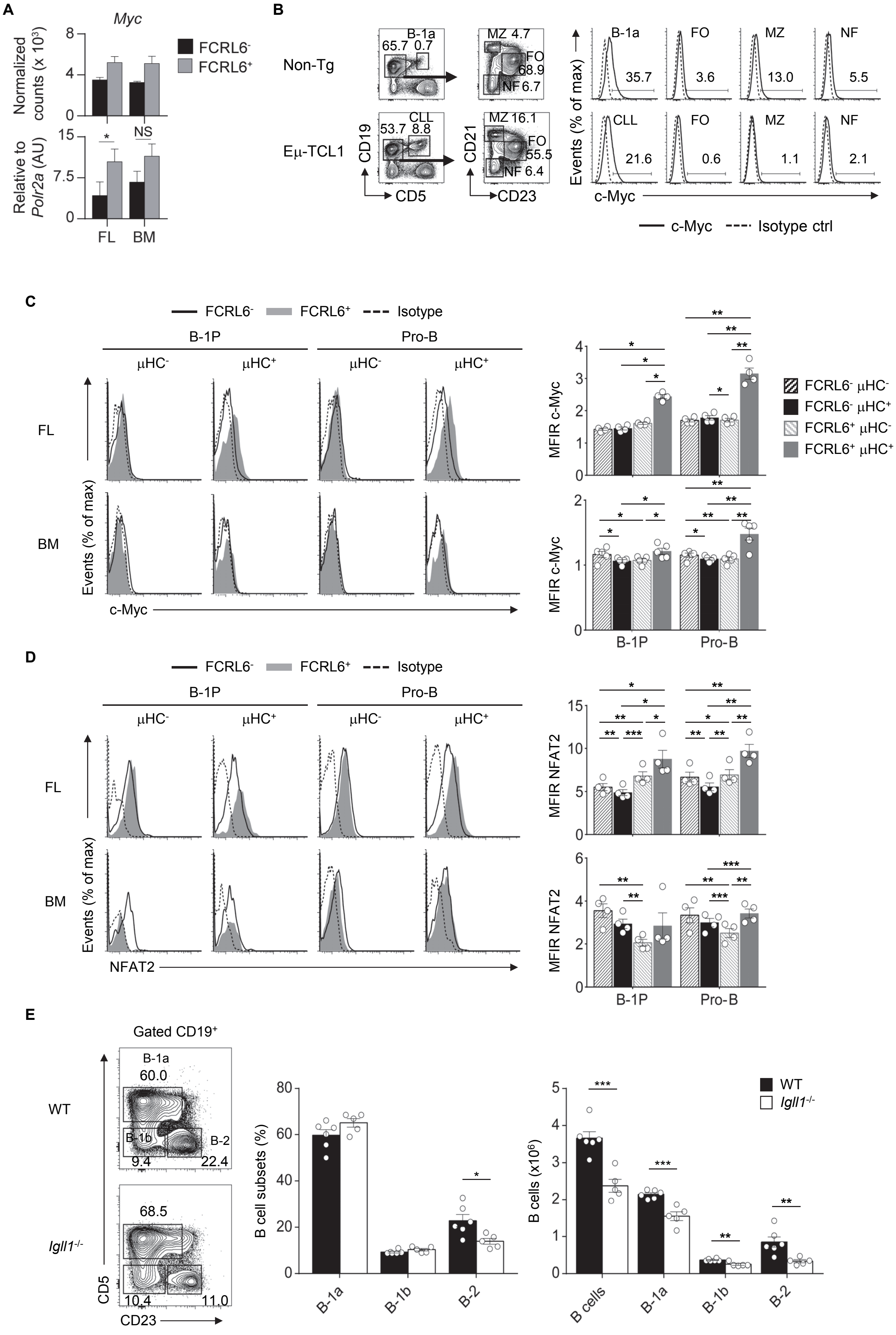

Supplementary Figure 7. Progenitor B cell transcription factor expression and PeC B cell constitution in λ5 KO mice. (**A**) RNA-seq (above) and RQ-PCR (below) analysis of *Myc* expression by FCRL6$^+$ and FCRL6$^-$ pro B cell subsets. *Myc* transcripts by RNA-seq (FL 0.57 x $\log_2$; P_{adj} = 0.01) represent normalized mean values from samples in duplicate and by RQ-PCR from three individually sorted samples performed in duplicate. (**B**) Intracellular staining of c-Myc in splenocyte subsets from C57BL/6 and Eμ-TCL1 Tg mice and analysis by flow cytometry. Numbers indicate the percentage of gated cells. Intracellular co-expression of c-Myc (**C**) or NFAT2 (**D**) and μHC in progenitor B cell subsets from FL and BM. (**E**) Analysis of PeC B cell subsets from WT BALB/c and *Igll1*$^{-/-}$ mice. Numbers adjacent to gates indicate frequencies. Each symbol represents an individual mouse. Small horizontal lines indicate s.e.m. *P < 0.05; **P < 0.01 and ***P < 0.001 as determined by paired (**A**, **C**-**D**) or unpaired Student's *t*-test (**E**). Data are representative of at least two independent experiments (**B**-**E**).

Supplementary Figure 8

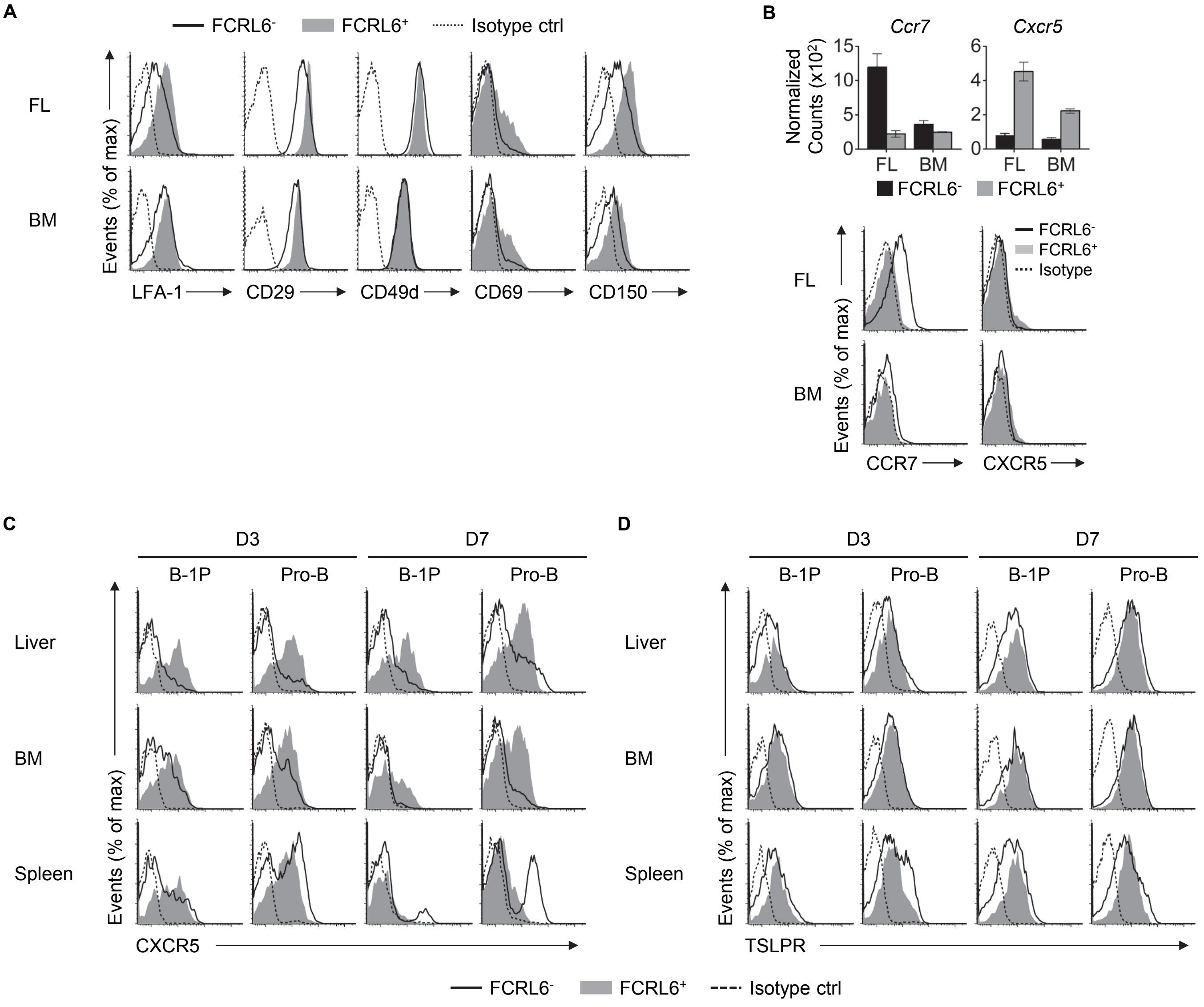

Supplementary Figure 8. Expression of migration, adhesion, and differentiation factors by FCRL6$^+$ and FCRL6$^-$ B cell progenitors. (**A**) Flow cytometry analysis of indicated surface markers from E18 FL and adult BM FCRL6$^+$ and FCRL6$^-$ pro B cells. (**B**) Normalized counts from RNA-seq (above) and surface staining by flow cytometry analysis (below) of CCR7 and CXCR5 by FL and BM pro B cells. Flow cytometry analysis of CXCR5 (**C**) and TSLPR (**D**) surface expression by D3 and D7 FCRL6$^+$ and FCRL6$^-$ progenitor B cell subpopulations from the indicated tissues. Small horizontal lines indicate s.e.m. Data are representative of at least two independent experiments (**A**-**B**) and pooled tissues from one of two independent experiments (**C**-**D**).
