# Supplementary material for: Fc Receptor-Like 6 (FCRL6) Discloses Progenitor B Cell Heterogeneity That Correlates With Pre-BCR Dependent and Independent Pathways of Natural Antibody Selection
Source: Front Immunol. 2020 Feb 14;11:82. doi: 10.3389/fimmu.2020.00082 (PMC7033751; doi:10.3389/fimmu.2020.00082)
Supplement: Supplementary file 3 [file Table_2.pdf]

Table supplement 2

Yang, Y, et al. *eLife* 4: e09083 (2015) doi: 10.7554/eLife.09083, PMID: 26422511

Splenic B-1a sequences (*n* = 150) from Tables 2 (T2), 3 (T3) and 7 (T7)

Source of clone	IGHV family	IGHV allele	IGHD	IGHJ	CDR-H3
T2-sB1a-11168-2w-1	IGHV1/J558	IGHV1-53	N/A	IGHJ2	ANDY
T2-sB1a-11168-2w-2	IGHV2/Q52	IGHV2-9	IGHD2-9	IGHJ4	AKHGYDAMDY
T2-sB1a-11168-2w-3	IGHV1/J558	IGHV1-55	IGHD1-1	IGHJ1	ARRYYGSSYWYFDV
T2-sB1a-11168-2w-4	IGHV1/J558	IGHV1-53	IGHD4-1	IGHJ2	ANWDY
T2-sB1a-11168-2w-5	IGHV11	IGHV11-2	IGHD2-6	IGHJ1	MRYSNYWYFDV
T2-sB1a-11168-2w-6	IGHV7/S107	IGHV7-1	N/A	IGHJ1	ARDAYYWYFDV
T2-sB1a-11168-2w-7	IGHV1/J558	IGHV1-26	N/A	IGHJ4	ATDYYAMDY
T2-sB1a-11168-2w-8	IGHV1/J558	IGHV1-55	IGHD1-1	IGHJ4	ARFYYYGSSYAMDY
T2-sB1a-11168-2w-9	IGHV1/J558	IGHV1-53	IGHD2-8	IGHJ2	AIYYLDY
T2-sB1a-11168-2w-10	IGHV2/Q52	IGHV2-6-2	IGHD1-1	IGHJ1	ARHYGSSYWYFDV
T2-sB1a-10654-3w-1	IGHV1/J558	IGHV1-55	IGHD1-1	IGHJ1	ARRYYGSSYWYFDV
T2-sB1a-10654-3w-2	IGHV1/J558	IGHV1-76	IGHD2-6	IGHJ4	ARSYSNYVMDY
T2-sB1a-10654-3w-3	IGHV7/S107	IGHV7-3	IGHD1-1	IGHJ2	ARYYGSNYFDY
T2-sB1a-10654-3w-4	IGHV1/J558	IGHV1-55	IGHD2-6	IGHJ3	ARGASYYSNWFAY
T2-sB1a-10654-3w-5	IGHV1/J558	IGHV1-53	IGHD4-1	IGHJ3	ALTGTAY
T2-sB1a-10654-3w-6	IGHV5/7183	IGHV5-9	IGHD4-1	IGHJ1	ARAGAGWYFDV
T2-sB1a-10654-3w-7	IGHV6/J606	IGHV6-6	IGHD2-6	IGHJ2	TYSNY
T2-sB1a-10654-3w-8	IGHV1/J558	IGHV1-53	IGHD4-1	IGHJ2	ARTGTYYFDY
T2-sB1a-10654-3w-9	IGHV1/J558	IGHV1-64	IGHD2-9	IGHJ2	AMVDY
T2-sB1a-10654-3w-10	IGHV1/J558	IGHV1-7	IGHD1-1	IGHJ2	ARWGTTVVGY
T2-sB1a-7632-2m-1	IGHV11	IGHV11-2	IGHD2-8	IGHJ1	MRYGNYWYFDV
T2-sB1a-7632-2m-2	IGHV11	IGHV11-2	IGHD2-6	IGHJ1	MRYSNYWYFDV
T2-sB1a-7632-2m-3	IGHV11	IGHV11-2	IGHD1-1	IGHJ1	MRYGSSYWYFDV
T2-sB1a-7632-2m-4	IGHV1/J558	IGHV1-55	N/A	IGHJ2	ATFSY
T2-sB1a-7632-2m-5	IGHV1/J558	IGHV1-55	IGHD1-1	IGHJ4	ARFYYYGSSYAMDY
T2-sB1a-7632-2m-6	IGHV1/J558	IGHV1-55	IGHD4-1	IGHJ1	ARIPNWVWYFDV
T2-sB1a-7632-2m-7	IGHV1/J558	IGHV1-7	IGHD1-1	IGHJ2	ARWDTTVVAPYYFDY
T2-sB1a-7632-2m-8	IGHV1/J558	IGHV1-26	IGHD1-1	IGHJ1	ARDYYGSSWYFDV
T2-sB1a-7632-2m-9	IGHV14/SM7	IGHV14-4	IGHD2-4	IGHJ4	TYYDYDLYAMDY
T2-sB1a-7632-2m-10	IGHV1/J558	IGHV1-9	IGHD1-1	IGHJ1	ARFITTVVATRYWYFDV
T2-sB1a-8699-4m-1	IGHV1/J558	IGHV1-64	IGHD2-4	IGHJ1	ARSADYGGYFDV
T2-sB1a-8699-4m-2	IGHV1/J558	IGHV1-80	N/A	IGHJ2	ARGAY
T2-sB1a-8699-4m-3	IGHV1/J558	IGHV1-76	IGHD2-4	IGHJ3	ARSYYDYPWFAY
T2-sB1a-8699-4m-4	IGHV1/J558	IGHV1-9	IGHD2-9	IGHJ4	ARRWLLNAMDY
T2-sB1a-8699-4m-5	IGHV1/J558	IGHV1-69	IGHD1-1	IGHJ3	ARPYYYGSSPWFAY
T2-sB1a-8699-4m-6	IGHV1/J558	IGHV1-4	IGHD2-4	IGHJ1	ARNDYPYWYFDV
T2-sB1a-8699-4m-7	IGHV1/J558	IGHV1-64	N/A	IGHJ2	ARSGDY
T2-sB1a-8699-4m-8	IGHV1/J558	IGHV1-53	IGHD2-14	IGHJ4	ARVIGDY
T2-sB1a-8699-4m-9	IGHV1/J558	IGHV1-55	N/A	IGHJ3	ARANY
T2-sB1a-8699-4m-10	IGHV1/J558	IGHV1-84	IGHD4-1	IGHJ4	AVNWDYAMDY
T2-sB1a-8708-5m-1	IGHV1/J558	IGHV1-55	N/A	IGHJ2	ASLTY
T2-sB1a-8708-5m-2	IGHV14/SM7	IGHV14-4	IGHD2-8	IGHJ4	TCNYH

T2-sB1a-8708-5m-3	IGHV1/J558	IGHV1-55	IGHD2-14	IGHJ2	LIGRNY
T2-sB1a-8708-5m-4	IGHV11	IGHV11-2	IGHD2-6	IGHJ1	MRYSNYWYFDV
T2-sB1a-8708-5m-5	IGHV2/Q52	IGHV2-3	IGHD1-1	IGHJ1	AKQPYYGSSYWYFDV
T2-sB1a-8708-5m-6	IGHV1/J558	IGHV1-66	IGHD1-1	IGHJ2	AGSSYAYYFDY
T2-sB1a-8708-5m-7	IGHV1/J558	IGHV1-26	IGHD2-8	IGHJ4	ARRGIDLLWYHYYAMDY
T2-sB1a-8708-5m-8	IGHV7/S107	IGHV7-3	IGHD3-2	IGHJ4	ARKSSGSRAMDY
T2-sB1a-8708-5m-9	IGHV7/S107	IGHV7-3	N/A	IGHJ4	ASYAMDY
T2-sB1a-8708-5m-10	IGHV1/J558	IGHV1-55	IGHD2-8	IGHJ1	ARLYYGNSYWYFDV
T2-sB1a-9867-6m-1	IGHV1/J558	IGHV1-55	IGHD1-1	IGHJ1	ARKYYPSWYFDV
T2-sB1a-9867-6m-2	IGHV1/J558	IGHV1-7	N/A	IGHJ2	AREGGKFY
T2-sB1a-9867-6m-3	IGHV1/J558	IGHV1-55	IGHD3-2	IGHJ4	AKSSGYAMDY
T2-sB1a-9867-6m-4	IGHV1/J558	IGHV1-85	IGHD1-1	IGHJ1	ARWVITTVARYFDV
T2-sB1a-9867-6m-5	IGHV1/J558	IGHV1-80	N/A	IGHJ2	ARGFY
T2-sB1a-9867-6m-6	IGHV1/J558	IGHV1-55	IGHD1-2	IGHJ4	AKEGGYYVRAMDY
T2-sB1a-9867-6m-7	IGHV1/J558	IGHV1-80	N/A	IGHJ4	ARSMDY
T2-sB1a-9867-6m-8	IGHV1/J558	IGHV1-64	N/A	IGHJ4	ASAMDY
T2-sB1a-9867-6m-9	IGHV1/J558	IGHV1-53	IGHD2-4	IGHJ3	TKGGYHDYDDGAWFVY
T2-sB1a-9867-6m-10	IGHV1/J558	IGHV1-55	N/A	IGHJ3	ARKFYPSWYFDV
T3-sB1a-1	IGHV6/J606	IGHV6-6	N/A	IGHJ2	TRWDY
T3-sB1a-2	IGHV11	IGHV11-2	IGHD2-6	IGHJ1	MRYSNYWYFDV
T3-sB1a-3	IGHV11	IGHV11-2	IGHD2-8	IGHJ1	MRYGNYWYFDV
T3-sB1a-4	IGHV11	IGHV11-2	IGHD1-1	IGHJ1	MRYGSSYWYFDV
T3-sB1a-5	IGHV10	IGHV10-1	IGHD1-1	IGHJ2	VRHYGSSYFDY
T3-sB1a-6	IGHV5/7183	IGHV5-6-1	IGHD1-1	IGHJ2	ARHYYGSSYYFDY
T3-sB1a-7	IGHV1/J558	IGHV1-53	N/A	IGHJ2	ARLDY
T3-sB1a-8	IGHV7/S107	IGHV7-1	IGHD1-1	IGHJ1	ARDYYGSSYWYFDV
T3-sB1a-9	IGHV1/J558	IGHV1-26	IGHD1-1	IGHJ1	ARDYYGSSWYFDV
T3-sB1a-10	IGHV14/SM7	IGHV14-3	IGHD4-1	IGHJ2	ANWDY
T3-sB1a-11	IGHV1/J558	IGHV1-19	IGHD4-1	IGHJ3	ATGTWFAY
T3-sB1a-12	IGHV7/S107	IGHV7-3	IGHD1-1	IGHJ4	ARYYYGSSYAMDY
T3-sB1a-13	IGHV1/J558	IGHV1-39	IGHD2-6	IGHJ4	ARYSNYYAMDY
T3-sB1a-14	IGHV1/J558	IGHV1-64	N/A	IGHJ2	ARDFDY
T3-sB1a-15	IGHV1/J558	IGHV1-9	IGHD2-6	IGHJ1	ARYYSNYWYFDV
T3-sB1a-16	IGHV1/J558	IGHV1-39	IGHD2-4	IGHJ4	ARYDYDYAMDY
T3-sB1a-17	IGHV2/Q52	IGHV2-6-2	IGHD1-1	IGHJ1	ARHYYGSSYWYFDV
T3-sB1a-18	IGHV1/J558	IGHV1-55	IGHD1-1	IGHJ4	ARFYYYGSSYAMDY
T3-sB1a-19	IGHV1/J558	IGHV1-7	N/A	IGHJ2	ARWDFDY
T3-sB1a-20	IGHV1/J558	IGHV1-80	N/A	IGHJ3	ARGAY
T3-sB1a-21	IGHV1/J558	IGHV1-26	N/A	IGHJ3	ARRFAY
T3-sB1a-22	IGHV1/J558	IGHV1-55	N/A	IGHJ2	ARRDY
T3-sB1a-23	IGHV1/J558	IGHV1-55	IGHD2-9	IGHJ1	ASYDGYYWYFDV
T3-sB1a-24	IGHV7/S107	IGHV7-3	N/A	IGHJ4	ASYAMDY
T3-sB1a-25	IGHV1/J558	IGHV1-78	N/A	IGHJ2	ARRYYFDY
T3-sB1a-26	IGHV1/J558	IGHV1-53	IGHD1-2	IGHJ2	ARNYYYFDY
T3-sB1a-27	IGHV3/36-60	IGHV3-8	IGHD2-8	IGHJ1	ARYYGNYWYFDV
T3-sB1a-28	IGHV1/J558	IGHV1-55	IGHD1-1	IGHJ1	ARRYYGSSYWYFDV
T3-sB1a-29	IGHV1/J558	IGHV1-22	N/A	IGHJ2	ARRLDY
T3-sB1a-30	IGHV1/J558	IGHV1-80	N/A	IGHJ3	ARFAY
T7-sB1a-GF1 -4m-1	IGHV11	IGHV11-2	IGHD1-1	IGHJ1	MRYGSSYWYFDV

T7-sB1a-GF1 -4m-2	IGHV1/J558	IGHV1-80	N/A	IGHJ2	ARGAY
T7-sB1a-GF1 -4m-3	IGHV2/Q52	IGHV2-2	IGHD2-9	IGHJ4	ARNPDGYYTYYYAMDY
T7-sB1a-GF1 -4m-4	IGHV5/7183	IGHV5-16	IGHD1-1	IGHJ1	ARDPFYYYGSSYWYFDV
T7-sB1a-GF1 -4m-5	IGHV11	IGHV11-2	IGHD2-6	IGHJ1	MRYSNYWYFDV
T7-sB1a-GF1 -4m-6	IGHV1/J558	IGHV1-55	N/A	IGHJ3	AITRAY
T7-sB1a-GF1 -4m-7	IGHV1/J558	IGHV1-55	IGHD1-1	IGHJ1	ARRYYGSSYWYFDV
T7-sB1a-GF1 -4m-8	IGHV1/J558	IGHV1-26	IGHD1-1	IGHJ2	ARSDYYGSSSLSY
T7-sB1a-GF1 -4m-9	IGHV1/J558	IGHV1-75	N/A	IGHJ2	ASGGNYFDY
T7-sB1a-GF1 -4m-10	IGHV1/J558	IGHV1-9	N/A	IGHJ2	ARSLYN
T7-sB1a-GF2 -4m-1	IGHV1/J558	IGHV1-53	IGHD1-1	IGHJ2	ARNYGSSYDY
T7-sB1a-GF2 -4m-2	IGHV14/SM7	IGHV14-4	IGHD1-1	IGHJ2	TRPSYYGSDY
T7-sB1a-GF2 -4m-3	IGHV5/7183	IGHV5-9-1	IGHD2-9	IGHJ4	TRESYDGYYVWYAMDY
T7-sB1a-GF2 -4m-4	IGHV14/SM7	IGHV14-3	N/A	IGHJ2	ARGDY
T7-sB1a-GF2 -4m-5	IGHV1/J558	IGHV1-53	IGHD4-1	IGHJ2	ASNWAY
T7-sB1a-GF2 -4m-6	IGHV11	IGHV11-2	IGHD2-6	IGHJ1	MRYSNYWYFDV
T7-sB1a-GF2 -4m-7	IGHV1/J558	IGHV1-9	IGHD1-1	IGHJ2	AKGDYYGSSYYFDY
T7-sB1a-GF2 -4m-8	IGHV10	IGHV10-1	IGHD3-2	IGHJ2	VRHGPRAFDY
T7-sB1a-GF2 -4m-9	IGHV1/J558	IGHV1-69	N/A	IGHJ2	ARLNGDY
T7-sB1a-GF2 -4m-10	IGHV11	IGHV11-2	IGHD2-8	IGHJ1	MRYGNYWYFDV
T7-sB1a-SPF1CT -4m-1	IGHV3/36-60	IGHV3-6	IGHD2-6	IGHJ1	ASYSNSDV
T7-sB1a-SPF1CT -4m-2	IGHV14/SM7	IGHV14-3	IGHD2-6	IGHJ4	ARVSYSRAMDY
T7-sB1a-SPF1CT -4m-3	IGHV1/J558	IGHV1-7	IGHD2-8	IGHJ4	ARSGNYGAMDY
T7-sB1a-SPF1CT -4m-4	IGHV2/Q52	IGHV2-6-8	IGHD1-1	IGHJ3	ASRLRSTFAY
T7-sB1a-SPF1CT -4m-5	IGHV1/J558	IGHV1-55	IGHD1-1	IGHJ4	ARVTTVHAMDY
T7-sB1a-SPF1CT -4m-6	IGHV1/J558	IGHV1-53	IGHD1-1	IGHJ1	ARNYGSSYWYFDV
T7-sB1a-SPF1CT -4m-7	IGHV1/J558	IGHV1-55	IGHD4-1	IGHJ4	ARTPNWEARDY
T7-sB1a-SPF1CT -4m-8	IGHV1/J558	IGHV1-55	IGHD1-1	IGHJ1	ARRYYGSSYWYFDV
T7-sB1a-SPF1CT -4m-9	IGHV1/J558	IGHV1-75	IGHD2-6	IGHJ2	ARPLLYRYYFDY
T7-sB1a-SPF1CT -4m-10	IGHV1/J558	IGHV1-9	IGHD1-1	IGHJ1	ARNYGSSYDWYFDV
T7-sB1a-SPF1S -4m-1	IGHV1/J558	IGHV1-64	IGHD2-4	IGHJ1	ARSADYGGYFDV
T7-sB1a-SPF1S -4m-2	IGHV1/J558	IGHV1-80	N/A	IGHJ2	ARGAY
T7-sB1a-SPF1S -4m-3	IGHV1/J558	IGHV1-76	IGHD2-4	IGHJ3	ARSYYDYPWFAY
T7-sB1a-SPF1S -4m-4	IGHV1/J558	IGHV1-9	IGHD2-9	IGHJ4	ARRWLLNAMDY
T7-sB1a-SPF1S -4m-5	IGHV1/J558	IGHV1-69	IGHD1-1	IGHJ3	ARPYYYGSSPWFAY
T7-sB1a-SPF1S -4m-6	IGHV1/J558	IGHV1-4	IGHD2-4	IGHJ1	ARNDYPYWYFDV
T7-sB1a-SPF1S -4m-7	IGHV1/J558	IGHV1-64	N/A	IGHJ2	ARSGDY
T7-sB1a-SPF1S -4m-8	IGHV1/J558	IGHV1-53	IGHD2-14	IGHJ4	ARVIGDY
T7-sB1a-SPF1S -4m-9	IGHV1/J558	IGHV1-55	N/A	IGHJ3	ARANY
T7-sB1a-SPF1S -4m-10	IGHV1/J558	IGHV1-84	IGHD4-1	IGHJ4	AVNWDYAMDY
T7-sB1a-SPF2CT -4m-1	IGHV1/J558	IGHV1-55	IGHD2-4	IGHJ4	ARGGIYYDYDEVYYYAMDY
T7-sB1a-SPF2CT -4m-2	IGHV11	IGHV11-2	IGHD2-6	IGHJ1	MRYSNYWYFDV
T7-sB1a-SPF2CT -4m-3	IGHV1/J558	IGHV1-26	IGHD1-1	IGHJ1	ARDYYGSSWYFDV
T7-sB1a-SPF2CT -4m-4	IGHV11	IGHV11-2	IGHD2-8	IGHJ1	MRYGNYWYFDV
T7-sB1a-SPF2CT -4m-5	IGHV11	IGHV11-2	IGHD1-1	IGHJ1	MRYGSSYWYFDV
T7-sB1a-SPF2CT -4m-6	IGHV1/J558	IGHV1-26	IGHD2-4	IGHJ4	ARYYDGYYGYYAMDY
T7-sB1a-SPF2CT -4m-7	IGHV1/J558	IGHV1-78	IGHD1-2	IGHJ1	ALITTWYFDV
T7-sB1a-SPF2CT -4m-8	IGHV1/J558	IGHV1-53	IGHD1-1	IGHJ2	ARHYYGSSWGY
T7-sB1a-SPF2CT -4m-9	IGHV1/J558	IGHV1-26	N/A	IGHJ2	ARSFSPYYFDY
T7-sB1a-SPF2CT -4m-10	IGHV1/J558	IGHV1-54	IGHD2-9	IGHJ2	ARSHGYYPFDY

T7-sB1a-SPF2S -4m-1	IGHV1/J558	IGHV1-80	N/A	IGHJ2	ARGNY
T7-sB1a-SPF2S -4m-2	IGHV1/J558	IGHV1-54	IGHD1-1	IGHJ1	ARWVYYGSSSYWYFDV
T7-sB1a-SPF2S -4m-3	IGHV1/J558	IGHV1-78	IGHD2-11	IGHJ4	ARSSNYAMDY
T7-sB1a-SPF2S -4m-4	IGHV7/S107	IGHV7-3	IGHD1-1	IGHJ4	ARYYYGSNYAMDY
T7-sB1a-SPF2S -4m-5	IGHV1/J558	IGHV1-80	N/A	IGHJ2	ARGAY
T7-sB1a-SPF2S -4m-6	IGHV1/J558	IGHV1-55	IGHD1-1	IGHJ1	ARRYYGSSYWYFDV
T7-sB1a-SPF2S -4m-7	IGHV1/J558	IGHV1-72	IGHD2-6	IGHJ1	ARSPYYSNYEGYFDV
T7-sB1a-SPF2S -4m-8	IGHV1/J558	IGHV1-55	IGHD1-1	IGHJ1	ARKNYGSSYWYFDV
T7-sB1a-SPF2S -4m-9	IGHV1/J558	IGHV1-80	IGHD2-8	IGHJ2	ARLEIYYGNYGRVFDV
T7-sB1a-SPF2S -4m-10	IGHV1/J558	IGHV1-9	IGHD1-1	IGHJ3	ARRDYYGSSYVLAY
