# Supplementary material for: Fc Receptor-Like 6 (FCRL6) Discloses Progenitor B Cell Heterogeneity That Correlates With Pre-BCR Dependent and Independent Pathways of Natural Antibody Selection
Source: Front Immunol. 2020 Feb 14;11:82. doi: 10.3389/fimmu.2020.00082 (PMC7033751; doi:10.3389/fimmu.2020.00082)
Supplement: Supplementary file 4 [file Table_3.pdf]

Table supplement 3

Published and unpublished mouse CLL sequences (Total clones *n* = 291 and with CDR-H3 sequences *n* = 258)

Clone	V gene	D gene	J gene	CDR-H3 amino acid seq	PubMed ID	Reference
XJY-NC TCL1_13	IGHV4-1	IGHD1-1	IGHJ2	ARHYYGSSYFDY	N/A	Chiorazzi lab unpublished
XJY-NC TCL_11	IGHV11-2	IGHD2-3	IGHJ1	MRYDGYYWYFDV	N/A	Chiorazzi lab unpublished
XJY-NC TCL1_G8_7	IGHV11-2	IGHD2-1	IGHJ1	MRYGNYWYFDV	N/A	Chiorazzi lab unpublished
XJY-NC TCL1_12-1	IGHV4-1	IGHD1-1	IGHJ3	ARHYYGSSWFAY	N/A	Chiorazzi lab unpublished
XJY-NC TCLBCG8-4	IGHV1-55	IGHD1-1	IGHJ2	AREENGLYYFDY	N/A	Chiorazzi lab unpublished
XJY-NC TCL1_G8_5	IGHV3-6	IGHD1-1	IGHJ2	ARANYYGSSYLYFDY	N/A	Chiorazzi lab unpublished
XJY-NC TCL1-HI-34	IGHV1-36	IGHD2-1	IGHJ2	ARSGNYDFDY	N/A	Chiorazzi lab unpublished
XJY-NC TCL1_18	IGHV1-85	IGHD1-1	IGHJ4	ARSGNYYGSDGVDY	N/A	Chiorazzi lab unpublished
XJY-NC TCL1_42	IGHV5-17	IGHD4-1	IGHJ4	AINWEGDYYAMDY	N/A	Chiorazzi lab unpublished
XJY-NC TCLBCG8-12	IGHV12-3	IGHD3-2	IGHJ1	AGDSSGYWYFDV	N/A	Chiorazzi lab unpublished
XJY-NC TCL-1	IGHV1-7	IGHD1-1	IGHJ2	APNYYGSSYFDY	N/A	Chiorazzi lab unpublished
XJY-NC TCL1-001	IGHV12-3	N/A	IGHJ1	AGDRRGYWYFDV	16864779	Yan et al. PNAS 99:11713-11718 (2006)
XJY-NC TCL1-002	IGHV12-3	IGHD4-1	IGHJ1	AGDRTGYWYFDV	16864779	Yan et al. PNAS 99:11713-11718 (2006)
XJY-NC TCL1-003	IGHV4-1	IGHD1-1	IGHJ2	ARHYYGSSYFDY	16864779	Yan et al. PNAS 99:11713-11718 (2006)
XJY-NC TCL1-004	IGHV4-1	IGHD1-1	IGHJ1	ARHYYGSSYFDY	16864779	Yan et al. PNAS 99:11713-11718 (2006)
XJY-NC TCL1-005	IGHV11-2	IGHD1-1	IGHJ1	MRYGSSYWYFDV	16864779	Yan et al. PNAS 99:11713-11718 (2006)
XJY-NC TCL1-006	IGHV11-2	IGHD2-5	IGHJ1	MRYGSNYWYFDV	16864779	Yan et al. PNAS 99:11713-11718 (2006)
XJY-NC TCL1-007	IGHV1-55	IGHD1-1	IGHJ4	ARIYYYGSSYAMDY	16864779	Yan et al. PNAS 99:11713-11718 (2006)
XJY-NC TCL1-008	IGHV1S61	IGHD1-1	IGHJ4	ARSYYDGSSYAMDY	16864779	Yan et al. PNAS 99:11713-11718 (2006)
XJY-NC TCL1-009	IGHV1-55	IGHD1-1	IGHJ1	ARRYYGSSWYFDV	16864779	Yan et al. PNAS 99:11713-11718 (2006)
XJY-NC TCL1-010	IGHV1-55	IGHD1-1	IGHJ1	ARIYYGSSYWYFDV	16864779	Yan et al. PNAS 99:11713-11718 (2006)
XJY-NC TCL1-011	IGHV1-55	IGHD2-3	IGHJ3	ARRWLLLFAY	16864779	Yan et al. PNAS 99:11713-11718 (2006)
XJY-NC TCL1-012	IGHV1-70	IGHD4-1	IGHJ3	ARLGGTLTGTSWFAY	16864779	Yan et al. PNAS 99:11713-11718 (2006)
XJY-NC TCL1-013	IGHV1S16	IGHD4-1	IGHJ2	TIYWVFDY	16864779	Yan et al. PNAS 99:11713-11718 (2006)
XJY-NC TCL1-014	IGHV1-82	IGHD2-12	IGHJ2	ARSYYSYYSYFDY	16864779	Yan et al. PNAS 99:11713-11718 (2006)
XJY-NC TCL1-015	IGHV1-42	IGHD4-1	IGHJ4	ALTGTDY	16864779	Yan et al. PNAS 99:11713-11718 (2006)
XJY-NC TCL1-016	IGHV1-15	IGHD1-1	IGHJ1	TRQKIWWQGYFDV	16864779	Yan et al. PNAS 99:11713-11718 (2006)
XJY-NC TCL1-017	IGHV3-6	IGHD2-3	IGHJ1	ARGDGYYVAY	16864779	Yan et al. PNAS 99:11713-11718 (2006)
XJY-NC TCL1-018	IGHV5-9	IGHD4-1	IGHJ2	ARNWDAFDY	16864779	Yan et al. PNAS 99:11713-11718 (2006)
XJY-NC TCL1-019	IGHV6-3	IGHD4-1	IGHJ2	TSWDVGY	16864779	Yan et al. PNAS 99:11713-11718 (2006)
XJY-NC TCL1-020	IGHV7-1	IGHD4-1	IGHJ4	ARDAGNWDYAMDY	16864779	Yan et al. PNAS 99:11713-11718 (2006)
IF-KR X12388 (10B10S)	IGHV1-39	IGHD2-1	IGHJ2	ARCRIYYGNYYFDY	3264787	Forster et al. EMBO 7:3693-3703 (1988)
IF-KR X12389 (10H12S)	IGHV1-59	IGHD2-13	IGHJ2	ASXFGDYPYYFDY	3264787	Forster et al. EMBO 7:3693-3703 (1988)
IF-KR X12390 (12B11S)	IGHV2-3	N/A	IGHJ4	APCSFAMDY	3264787	Forster et al. EMBO 7:3693-3703 (1988)
IF-KR X12391 (12F10S)	IGHV1-55	IGHD4-1	IGHJ2	ARVGWDGYYFDY	3264787	Forster et al. EMBO 7:3693-3703 (1988)
IF-KR X12392 (13G3S)	IGHV1-26	IGHD1-1	IGHJ2	ARAHYYGSSSPYYFDY	3264787	Forster et al. EMBO 7:3693-3703 (1988)
IF-KR X12393 (1A1PC)	IGHV1-55	IGHD1-1	IGHJ2	ARKDYYGSGDY	3264787	Forster et al. EMBO 7:3693-3703 (1988)
IF-KR X12394 (1B1S)	IGHV1-26	N/A	IGHJ2	AGNFDY	3264787	Forster et al. EMBO 7:3693-3703 (1988)
IF-KR X12395 (3B9PC)	IGHV1-53	N/A	IGHJ1	ARGGRYFDV	3264787	Forster et al. EMBO 7:3693-3703 (1988)
IF-KR X12396 (3D11PC)	IGHV1-54	IGHD2-1	IGHJ1	AREGLYYDWYFDV	3264787	Forster et al. EMBO 7:3693-3703 (1988)
IF-KR X12397 (6C7S)	IGHV1-52	IGHD1-1	IGHJ1	AYYGSSYWYFDV	3264787	Forster et al. EMBO 7:3693-3703 (1988)
IF-KR X12398 (7E9S)	IGHV1-55	IGHD4-1	IGHJ4	AVNWDDAMDY	3264787	Forster et al. EMBO 7:3693-3703 (1988)
IF-KR X12399 (8B3S)	IGHV1-20	IGHD2-4	IGHJ3	ARGSYYDYFAY	3264787	Forster et al. EMBO 7:3693-3703 (1988)
IF-KR X12412 (B16.2)	IGHV1-55	IGHD2-3	IGHJ1	ASYDGYYWYFDV	3264787	Forster et al. EMBO 7:3693-3703 (1988)
KH-RH T1-TC+ St1-1	IGHV12-3	IGHD2-3	IGHJ1	AGDYDGYWYFDV	27899442	Hayakawa et al. JEM 213:3007-3024 (2016)
KH-RH T1-TC+ St1-2	IGHV12-3	IGHD2-3	IGHJ1	AGDYDGYWYFDV	27899442	Hayakawa et al. JEM 213:3007-3024 (2016)
KH-RH T1-TC+ St1-3	IGHV12-3	IGHD2-3	IGHJ1	AGDYDGYWYFDV	27899442	Hayakawa et al. JEM 213:3007-3024 (2016)
KH-RH T1-TC+ St1-4	IGHV12-3	IGHD2-3	IGHJ1	AGDYDGYWYFDV	27899442	Hayakawa et al. JEM 213:3007-3024 (2016)
KH-RH T1-TC+ St1-5	IGHV12-3	IGHD2-3	IGHJ1	AGDYDGYWYFDV	27899442	Hayakawa et al. JEM 213:3007-3024 (2016)
KH-RH T1-TC+ St1-6	IGHV12-3	IGHD2-3	IGHJ1	AGDYDGYWYFDV	27899442	Hayakawa et al. JEM 213:3007-3024 (2016)
KH-RH T1-TC+ St1-7	IGHV12-3	IGHD2-3	IGHJ1	AGDYDGYWYFDV	27899442	Hayakawa et al. JEM 213:3007-3024 (2016)
KH-RH T1-TC+ St1-8	IGHV12-3	IGHD2-3	IGHJ1	AGDYDGYWYFDV	27899442	Hayakawa et al. JEM 213:3007-3024 (2016)
KH-RH T1-TC+ St1-9	IGHV12-3	IGHD2-3	IGHJ1	AGDYDGYWYFDV	27899442	Hayakawa et al. JEM 213:3007-3024 (2016)
KH-RH T1-TC+ St1-10	IGHV12-3	IGHD2-3	IGHJ1	AGDYDGYWYFDV	27899442	Hayakawa et al. JEM 213:3007-3024 (2016)
KH-RH T1-TC+ St1-11	IGHV12-3	IGHD2-3	IGHJ1	AGDYDGYWYFDV	27899442	Hayakawa et al. JEM 213:3007-3024 (2016)
KH-RH T1-TC+ St1-12	IGHV12-3	IGHD2-3	IGHJ1	AGDYDGYWYFDV	27899442	Hayakawa et al. JEM 213:3007-3024 (2016)
KH-RH T1-TC+ St1-13	IGHV12-3	IGHD2-3	IGHJ1	AGDYDGYWYFDV	27899442	Hayakawa et al. JEM 213:3007-3024 (2016)
KH-RH T1-TC+ St1-14	IGHV12-3	IGHD2-3	IGHJ1	AGDYDGYWYFDV	27899442	Hayakawa et al. JEM 213:3007-3024 (2016)
KH-RH T1-TC+ St1-15	IGHV12-3	IGHD2-3	IGHJ1	AGDYDGYWYFDV	27899442	Hayakawa et al. JEM 213:3007-3024 (2016)
KH-RH T1-TC+ St1-16	IGHV12-3	IGHD2-3	IGHJ1	AGDYDGYWYFDV	27899442	Hayakawa et al. JEM 213:3007-3024 (2016)
KH-RH T1-TC+ St1-17	IGHV12-3	IGHD2-3	IGHJ1	AGDYDGYWYFDV	27899442	Hayakawa et al. JEM 213:3007-3024 (2016)
KH-RH T1-TC+ St1-18	IGHV12-3	IGHD2-3	IGHJ1	AGDYDGYWYFDV	27899442	Hayakawa et al. JEM 213:3007-3024 (2016)
KH-RH T1-TC+ St1-1	IGHV12-3	IGHD2-3	IGHJ1	AGDEDGYWYFDV	27899442	Hayakawa et al. JEM 213:3007-3024 (2016)
KH-RH T1-TC+ St1-2	IGHV12-3	IGHD2-3	IGHJ1	AGDRDGYWYFDV	27899442	Hayakawa et al. JEM 213:3007-3024 (2016)
KH-RH T1-TC+ St1-3	IGHV12-3	IGHD2-3	IGHJ1	AGDRYGYWYFDV	27899442	Hayakawa et al. JEM 213:3007-3024 (2016)
KH-RH T1-TC+ St1-4	IGHV12-3	IGHD2-3	IGHJ1	AGDRYGYWYFDV	27899442	Hayakawa et al. JEM 213:3007-3024 (2016)
KH-RH T1-TC+ St1-5	IGHV12-3	IGHD2-7	IGHJ1	AGDLYGYWYFDV	27899442	Hayakawa et al. JEM 213:3007-3024 (2016)
KH-RH T1-TC+ St1-6	IGHV12-3	IGHD2-7	IGHJ1	AGDLYGYWYFDV	27899442	Hayakawa et al. JEM 213:3007-3024 (2016)
KH-RH T1-TC+ St1-7	IGHV12-3	IGHD2-7	IGHJ1	AGDLYGYWYFDV	27899442	Hayakawa et al. JEM 213:3007-3024 (2016)
KH-RH T1-TC+ St1-8	IGHV12-3	IGHD2-7	IGHJ1	AGDRSGYWYFDV	27899442	Hayakawa et al. JEM 213:3007-3024 (2016)
KH-RH T1-TC+ St1-9	IGHV12-3	IGHD1-2	IGHJ1	AGDYYGYWYFDV	27899442	Hayakawa et al. JEM 213:3007-3024 (2016)
KH-RH T1-TC+ St1-10	IGHV12-3	IGHD2-9	IGHJ1	AGDYYGYWYFDV	27899442	Hayakawa et al. JEM 213:3007-3024 (2016)
KH-RH T1-TC+ St1-11	IGHV12-3	IGHD4-1	IGHJ1	AGDNLGYWYFDV	27899442	Hayakawa et al. JEM 213:3007-3024 (2016)
KH-RH T1-TC+ St1-12	IGHV12-3	IGHD4-1	IGHJ1	AGDRTGYWYFDV	27899442	Hayakawa et al. JEM 213:3007-3024 (2016)
KH-RH T1-TC+ St1-13	IGHV12-3	IGHD4-1	IGHJ1	AGDRTGYWYFDV	27899442	Hayakawa et al. JEM 213:3007-3024 (2016)
KH-RH T1-TC+ St1-14	IGHV12-3	IGHD4-1	IGHJ1	AGDLTGYWYFDV	27899442	Hayakawa et al. JEM 213:3007-3024 (2016)
KH-RH T1-TC+ St1-15	IGHV12-3	IGHD4-1	IGHJ1	AGDNWGYWYFDV	27899442	Hayakawa et al. JEM 213:3007-3024 (2016)
KH-RH T1-TC+ St1-16	IGHV12-3	IGHD3-2	IGHJ1	AGDRGGYWYFDV	27899442	Hayakawa et al. JEM 213:3007-3024 (2016)

KH-RH T1-TC+ St1-17	IGHV12-3	IGHD3-3	IGHJ1	AGDRLGYWYFDV	27899442	Hayakawa et al. JEM 213:3007-3024 (2016)
KH-RH T1-TC+ St1-18	IGHV12-3	IGHD3-2	IGHJ1	AGDSSGYWYFDV	27899442	Hayakawa et al. JEM 213:3007-3024 (2016)
KH-RH T1-TC+ St1-19	IGHV12-3	IGHD2-1	IGHJ2	AGDRYGNYYFDY	27899442	Hayakawa et al. JEM 213:3007-3024 (2016)
KH-RH T1-TC+ St1-20	IGHV12-3	IGHD2-3	IGHJ3	AGDRDGYYFFAY	27899442	Hayakawa et al. JEM 213:3007-3024 (2016)
KH-RH T1-TC+ St1-21	IGHV12-3	IGHD4-1	IGHJ4	AGDGTGYYAMDY	27899442	Hayakawa et al. JEM 213:3007-3024 (2016)
KH-RH T1-TC+ St1-22	IGHV12-3	IGHD2-3	IGHJ4	AGDRSGYYAMDY	27899442	Hayakawa et al. JEM 213:3007-3024 (2016)
KH-RH T1-TC+ St2-1	IGHV1-55	IGHD1-1	IGHJ1	ARCYYGSSYWYFDV	27899442	Hayakawa et al. JEM 213:3007-3024 (2016)
KH-RH T1-TC+ St2-2	IGHV1-55	IGHD1-1	IGHJ1	ARCYYGSSYWYFDV	27899442	Hayakawa et al. JEM 213:3007-3024 (2016)
KH-RH T1-TC+ St2-3	IGHV1-55	IGHD1-1	IGHJ1	ARRYYGSSYWYFDV	27899442	Hayakawa et al. JEM 213:3007-3024 (2016)
KH-RH T1-TC+ St2-4	IGHV1-55	IGHD1-1	IGHJ1	ARIYYGSSYWYFDV	27899442	Hayakawa et al. JEM 213:3007-3024 (2016)
KH-RH T1-TC+ St2-5	IGHV1-55	IGHD1-1	IGHJ1	ARRDYGSSYWYFDV	27899442	Hayakawa et al. JEM 213:3007-3024 (2016)
KH-RH T1-TC+ St2-6	IGHV1-55	IGHD1-1	IGHJ1	ARKGYYGSSWYFDV	27899442	Hayakawa et al. JEM 213:3007-3024 (2016)
KH-RH T1-TC+ St2-7	IGHV1-55	IGHD1-1	IGHJ1	AREVYGSSLWFFDV	27899442	Hayakawa et al. JEM 213:3007-3024 (2016)
KH-RH T1-TC+ St2-8	IGHV1-55	IGHD1-1	IGHJ1	ARRNYGSSYWYFDV	27899442	Hayakawa et al. JEM 213:3007-3024 (2016)
KH-RH T1-TC+ St2-9	IGHV1-55	IGHD1-1	IGHJ1	ARLDYGSSYWYFDV	27899442	Hayakawa et al. JEM 213:3007-3024 (2016)
KH-RH T1-TC+ St2-10	IGHV1-55	IGHD1-1	IGHJ1	ARLRYGSSYWYFDV	27899442	Hayakawa et al. JEM 213:3007-3024 (2016)
KH-RH T1-TC+ St2-11	IGHV1-55	IGHD1-1	IGHJ1	ARRYYYGSSWYFDV	27899442	Hayakawa et al. JEM 213:3007-3024 (2016)
KH-RH T1-TC+ St2-12	IGHV1-55	IGHD1-1	IGHJ4	ARFYYYGSSYAMDY	27899442	Hayakawa et al. JEM 213:3007-3024 (2016)
KH-RH T1-TC+ St2-13	IGHV1-55	IGHD1-1	IGHJ4	ARFYYYGSSYAMDY	27899442	Hayakawa et al. JEM 213:3007-3024 (2016)
KH-RH T1-TC+ St2-14	IGHV1-55	IGHD1-1	IGHJ4	ARFYYGSSYAMDY	27899442	Hayakawa et al. JEM 213:3007-3024 (2016)
KH-RH T1-TC+ St2-15	IGHV1-55	IGHD1-1	IGHJ4	ARDFYGSSYAMDY	27899442	Hayakawa et al. JEM 213:3007-3024 (2016)
KH-RH T1-TC+ St2-16	IGHV1-55	IGHD1-1	IGHJ4	ARDFYYGSSYAMDY	27899442	Hayakawa et al. JEM 213:3007-3024 (2016)
KH-RH T1-TC+ St2-17	IGHV1-55	IGHD1-1	IGHJ2	ARFYYYGSSYYAMDY	27899442	Hayakawa et al. JEM 213:3007-3024 (2016)
KH-RH T1-TC+ St2-18	IGHV1-55	IGHD1-1	IGHJ2	ARRDYYGSSYYFDY	27899442	Hayakawa et al. JEM 213:3007-3024 (2016)
KH-RH T1-TC+ St2-19	IGHV1-55	IGHD1-1	IGHJ2	ARRYYYGSSYYFDY	27899442	Hayakawa et al. JEM 213:3007-3024 (2016)
KH-RH T1-TC+ St3-1	IGHV2-9	IGHD2-3	IGHJ4	AKRLLRYYAMDY	27899442	Hayakawa et al. JEM 213:3007-3024 (2016)
KH-RH T1-TC+ St3-2	IGHV2-9	IGHD2-3	IGHJ4	AKRLLRYYAMDY	27899442	Hayakawa et al. JEM 213:3007-3024 (2016)
KH-RH T1-TC+ St3-3	IGHV2-9	IGHD2-3	IGHJ4	AKRLLRYYAMDY	27899442	Hayakawa et al. JEM 213:3007-3024 (2016)
KH-RH T1-TC+ St3-4	IGHV2-9	IGHD2-3	IGHJ4	AKRLLRYYAMDY	27899442	Hayakawa et al. JEM 213:3007-3024 (2016)
KH-RH T1-TC+ St3-5	IGHV2-9	IGHD2-3	IGHJ4	AKRLLRYYAMDY	27899442	Hayakawa et al. JEM 213:3007-3024 (2016)
KH-RH T1-TC+ St3-6	IGHV2-9	IGHD2-3	IGHJ4	AKRLLRYYAMDY	27899442	Hayakawa et al. JEM 213:3007-3024 (2016)
KH-RH T1-TC+ St3-7	IGHV2-9	IGHD2-3	IGHJ4	AKRLLRYYAMDY	27899442	Hayakawa et al. JEM 213:3007-3024 (2016)
KH-RH T1-TC+ St3-8	IGHV2-9	IGHD2-3	IGHJ4	AKRLLRYYAMDY	27899442	Hayakawa et al. JEM 213:3007-3024 (2016)
KH-RH T1-TC+ St3-9	IGHV2-9	IGHD2-3	IGHJ4	AKRLLRYYAMDY	27899442	Hayakawa et al. JEM 213:3007-3024 (2016)
KH-RH T1-TC+ St3-10	IGHV2-9	IGHD2-3	IGHJ4	AKRLLRYYAMDY	27899442	Hayakawa et al. JEM 213:3007-3024 (2016)
KH-RH T1-TC+ St3-11	IGHV2-9	IGHD2-3	IGHJ4	AKRLLRYYAMDY	27899442	Hayakawa et al. JEM 213:3007-3024 (2016)
KH-RH T1-TC+ St3-12	IGHV2-9	IGHD1-1	IGHJ4	AKRLLRYYAMDY	27899442	Hayakawa et al. JEM 213:3007-3024 (2016)
KH-RH T1-TC+ St3-13	IGHV2-9	IGHD1-1	IGHJ4	AKRLLRYYAMDY	27899442	Hayakawa et al. JEM 213:3007-3024 (2016)
KH-RH T1-TC+ St3-14	IGHV2-9	IGHD1-1	IGHJ4	AKRLLRYYAMDY	27899442	Hayakawa et al. JEM 213:3007-3024 (2016)
KH-RH T1-TC+ St3-15	IGHV2-9	IGHD2-3	IGHJ3	AKSRGWLLPFAY	27899442	Hayakawa et al. JEM 213:3007-3024 (2016)
KH-RH T1-TC+ St4-1	IGHV1-52	IGHD2-1	IGHJ1	ARYYGNYWYFDV	27899442	Hayakawa et al. JEM 213:3007-3024 (2016)
KH-RH T1-TC+ St4-2	IGHV1-52	IGHD2-1	IGHJ1	ARYYGNYWYFDV	27899442	Hayakawa et al. JEM 213:3007-3024 (2016)
KH-RH T1-TC+ St4-3	IGHV1-52	IGHD2-1	IGHJ1	ATYYGNYWYFDV	27899442	Hayakawa et al. JEM 213:3007-3024 (2016)
KH-RH T1-TC+ St4-4	IGHV1-52	IGHD2-5	IGHJ1	ASYYSNYWYFDV	27899442	Hayakawa et al. JEM 213:3007-3024 (2016)
KH-RH T1-TC+ St4-5	IGHV1-52	IGHD2-5	IGHJ1	ASYYSNYWYFDV	27899442	Hayakawa et al. JEM 213:3007-3024 (2016)
KH-RH T1-TC+ St4-6	IGHV1-52	IGHD2-8	IGHJ1	AIYYGNYWYFDV	27899442	Hayakawa et al. JEM 213:3007-3024 (2016)
KH-RH T1-TC+ St4-7	IGHV1-52	IGHD2-8	IGHJ1	ARWGNPYWYFDV	27899442	Hayakawa et al. JEM 213:3007-3024 (2016)
KH-RH T1-TC+ St4-8	IGHV1-52	IGHD2-8	IGHJ1	ARHYYGNPYWYFDV	27899442	Hayakawa et al. JEM 213:3007-3024 (2016)
KH-RH T1-TC+ St4-9	IGHV1-52	IGHD2-13	IGHJ1	ARHYGVYWYFDV	27899442	Hayakawa et al. JEM 213:3007-3024 (2016)
KH-RH T1-TC+ St4-10	IGHV1-52	IGHD1-1	IGHJ4	ARSYYDGSYYYAMDY	27899442	Hayakawa et al. JEM 213:3007-3024 (2016)
KH-RH T1-TC+ St5-1	VH11-2	IGHD2-5	IGHJ1	MRYSNYWYFDV	27899442	Hayakawa et al. JEM 213:3007-3024 (2016)
KH-RH T1-TC+ St5-2	VH11-2	IGHD2-5	IGHJ1	MRYSNYWYFDV	27899442	Hayakawa et al. JEM 213:3007-3024 (2016)
KH-RH T1-TC+ St5-3	VH11-2	IGHD1-1	IGHJ1	MRYGHYWYFDV	27899442	Hayakawa et al. JEM 213:3007-3024 (2016)
KH-RH T1-TC+ St5-4	VH11-2	IGHD1-1	IGHJ1	MRYPYYYYGSSYWYFDV	27899442	Hayakawa et al. JEM 213:3007-3024 (2016)
KH-RH S3-TC+ OVH-1	IGHV1-9	IGHD1-1	IGHJ4	ARGYGSMDAMDY	27899442	Hayakawa et al. JEM 213:3007-3024 (2016)
KH-RH S3-TC+ OVH-2	IGHV1-9	IGHD1-1	IGHJ4	ARGYGSMDAMDY	27899442	Hayakawa et al. JEM 213:3007-3024 (2016)
KH-RH S3-TC+ OVH-3	IGHV1-9	IGHD1-1	IGHJ1	ANYGSSWYFDV	27899442	Hayakawa et al. JEM 213:3007-3024 (2016)
KH-RH S3-TC+ OVH-4	IGHV1-9	IGHD4-1	IGHJ4	ARLWDVGYYAMDY	27899442	Hayakawa et al. JEM 213:3007-3024 (2016)
KH-RH S3-TC+ OVH-5	IGHV1-82	IGHD2-4	IGHJ3	ARSGLRRGFGY	27899442	Hayakawa et al. JEM 213:3007-3024 (2016)
KH-RH S3-TC+ OVH-6	IGHV1-82	IGHD2-7	IGHJ3	ARSYGYSGGFAY	27899442	Hayakawa et al. JEM 213:3007-3024 (2016)
KH-RH S3-TC+ OVH-7	IGHV1-82	IGHD1-1	IGHJ3	AFSSGYITWFAY	27899442	Hayakawa et al. JEM 213:3007-3024 (2016)
KH-RH S3-TC+ OVH-8	IGHV1-82	IGHD1-1	IGHJ2	YYGSRYYFDY	27899442	Hayakawa et al. JEM 213:3007-3024 (2016)
KH-RH S3-TC+ OVH-9	IGHV1-19	IGHD1-1	IGHJ3	ARPYYYGSTWFAY	27899442	Hayakawa et al. JEM 213:3007-3024 (2016)
KH-RH S3-TC+ OVH-10	IGHV1-19	IGHD1-1	IGHJ2	ARGNYYGSSDY	27899442	Hayakawa et al. JEM 213:3007-3024 (2016)
KH-RH S3-TC+ OVH-11	IGHV1-19	IGHD1-1	IGHJ2	ARGIITTVVAYYFDY	27899442	Hayakawa et al. JEM 213:3007-3024 (2016)
KH-RH S3-TC+ OVH-12	IGHV1-18	IGHD4-1	IGHJ2	ARVPTGYYFDY	27899442	Hayakawa et al. JEM 213:3007-3024 (2016)
KH-RH S3-TC+ OVH-13	IGHV1-18	IGHD2-4	IGHJ3	ARSGDYGFAY	27899442	Hayakawa et al. JEM 213:3007-3024 (2016)
KH-RH S3-TC+ OVH-14	IGHV1-78	IGHD1-1	IGHJ2	ARSALRY	27899442	Hayakawa et al. JEM 213:3007-3024 (2016)
KH-RH S3-TC+ OVH-15	IGHV1-78	IGHD1-1	IGHJ2	ARGIYYGSSLDY	27899442	Hayakawa et al. JEM 213:3007-3024 (2016)
KH-RH S3-TC+ OVH-16	IGHV1-78	N/A	IGHJ4	SAMDY	27899442	Hayakawa et al. JEM 213:3007-3024 (2016)
KH-RH S3-TC+ OVH-17	IGHV1-63	IGHD1-1	IGHJ2	ARNYYGSSYYFDY	27899442	Hayakawa et al. JEM 213:3007-3024 (2016)
KH-RH S3-TC+ OVH-18	IGHV1-63	IGHD1-1	IGHJ2	ARWSDYGSSPYYFDY	27899442	Hayakawa et al. JEM 213:3007-3024 (2016)
KH-RH S3-TC+ OVH-19	IGHV1-63	IGHD1-1	IGHJ4	ARLNYYGSSYAMDY	27899442	Hayakawa et al. JEM 213:3007-3024 (2016)
KH-RH S3-TC+ OVH-20	IGHV1-12	IGHD2-5	IGHJ2	ASNYYYY	27899442	Hayakawa et al. JEM 213:3007-3024 (2016)
KH-RH S3-TC+ OVH-21	IGHV1-9	IGHD1-1	IGHJ4	SVYYGSSYYYAMDY	27899442	Hayakawa et al. JEM 213:3007-3024 (2016)
KH-RH S3-TC+ OVH-22	IGHV1-69	IGHD1-1	IGHJ1	AKRGYYGSRSWYFDV	27899442	Hayakawa et al. JEM 213:3007-3024 (2016)
KH-RH S3-TC+ OVH-23	IGHV1S33	IGHD1-1	IGHJ2	ARSALRY	27899442	Hayakawa et al. JEM 213:3007-3024 (2016)
KH-RH S3-TC+ OVH-24	IGHV1S74	IGHD2-14	IGHJ4	ARGGDRYDYAMDY	27899442	Hayakawa et al. JEM 213:3007-3024 (2016)
KH-RH S3-TC+ OVH-25	IGHV1S133	IGHD4-1	IGHJ2	ARLGRDY	27899442	Hayakawa et al. JEM 213:3007-3024 (2016)
KH-RH S3-TC+ OVH-26	IGHV1-37	IGHD2-3	IGHJ4	ARYYYAMDY	27899442	Hayakawa et al. JEM 213:3007-3024 (2016)
KH-RH S3-TC+ OVH-27	IGHV1-11	IGHD1-1	IGHJ1	ARGYYGSSYWYFDV	27899442	Hayakawa et al. JEM 213:3007-3024 (2016)
KH-RH S3-TC+ OVH-28	IGHV1-81	IGHD2-8	IGHJ4	ARREGNYLYYYAMDY	27899442	Hayakawa et al. JEM 213:3007-3024 (2016)
KH-RH S3-TC+ OVH-29	IGHV1-7	IGHD4-1	IGHJ3	ARWLGQAY	27899442	Hayakawa et al. JEM 213:3007-3024 (2016)

KH-RH S3-TC+ OVH-30	IGHV1S135	IGHD2-4	IGHJ2	ARHSDYDYSDY	27899442	Hayakawa et al. JEM 213:3007-3024 (2016)
KH-RH S3-TC+ OVH-31	IGHV1-74	IGHD1-1	IGHJ4	ARGGSSGTDYAMDY	27899442	Hayakawa et al. JEM 213:3007-3024 (2016)
KH-RH S3-TC+ OVH-32	IGHV1-74	IGHD2-5	IGHJ2	AIGGYSNYVDFDY	27899442	Hayakawa et al. JEM 213:3007-3024 (2016)
KH-RH S3-TC+ OVH-33	IGHV1-26	IGHD1-1	IGHJ1	ARYYGSSYWYFDV	27899442	Hayakawa et al. JEM 213:3007-3024 (2016)
KH-RH S3-TC+ OVH-34	IGHV1-7	IGHD1-1	IGHJ2	ARSHYGSSYGGYFDY	27899442	Hayakawa et al. JEM 213:3007-3024 (2016)
KH-RH S3-TC+ OVH-35	IGHV1-80	IGHD2-3	IGHJ3	ARSGGGYYWFAY	27899442	Hayakawa et al. JEM 213:3007-3024 (2016)
KH-RH S3-TC+ OVH-36	IGHV7-3	IGHD1-1	IGHJ4	ARYYYGSSYAMDY	27899442	Hayakawa et al. JEM 213:3007-3024 (2016)
KH-RH S3-TC+ OVH-37	IGHV7-3	IGHD1-1	IGHJ4	ARYYYGSSYAMDY	27899442	Hayakawa et al. JEM 213:3007-3024 (2016)
KH-RH S3-TC+ OVH-38	IGHV7-3	IGHD2-3	IGHJ3	ARDAMMVWFAY	27899442	Hayakawa et al. JEM 213:3007-3024 (2016)
KH-RH S3-TC+ OVH-39	IGHV7-3	IGHD2-14	IGHJ2	ARDRYDYFDY	27899442	Hayakawa et al. JEM 213:3007-3024 (2016)
KH-RH S3-TC+ OVH-40	IGHV7-3	IGHD4-1	IGHJ2	ARSLYWDEGYFDY	27899442	Hayakawa et al. JEM 213:3007-3024 (2016)
KH-RH S3-TC+ OVH-41	IGHV3-2	IGHD1-1	IGHJ2	ARYYYGSSYYFDY	27899442	Hayakawa et al. JEM 213:3007-3024 (2016)
KH-RH S3-TC+ OVH-42	IGHV3-2	IGHD1-1	IGHJ2	ARYYYGSSYYFDY	27899442	Hayakawa et al. JEM 213:3007-3024 (2016)
KH-RH S3-TC+ OVH-43	IGHV3-2	IGHD2-4	IGHJ4	ARFYDYDYYAMDY	27899442	Hayakawa et al. JEM 213:3007-3024 (2016)
KH-RH S3-TC+ OVH-44	IGHV3-2	IGHD2-1	IGHJ2	ARCGNYEDYFDY	27899442	Hayakawa et al. JEM 213:3007-3024 (2016)
KH-RH S3-TC+ OVH-45	IGHV3-8	IGHD1-1	IGHJ1	YYGSSYWYFDV	27899442	Hayakawa et al. JEM 213:3007-3024 (2016)
KH-RH S3-TC+ OVH-46	IGHV3-8	IGHD1-1	IGHJ2	ARMGKIFPYYGSSYFDY	27899442	Hayakawa et al. JEM 213:3007-3024 (2016)
KH-RH S3-TC+ OVH-47	IGHV3-8	IGHD1-1	IGHJ2	ARYYYGSSDWYFDV	27899442	Hayakawa et al. JEM 213:3007-3024 (2016)
KH-RH S3-TC+ OVH-48	IGHV3-6	IGHD1-1	IGHJ4	ARDYGSSLYYYAMDY	27899442	Hayakawa et al. JEM 213:3007-3024 (2016)
KH-RH S3-TC+ OVH-49	IGHV3-6	IGHD4-1	IGHJ2	AREGGNWYYFDY	27899442	Hayakawa et al. JEM 213:3007-3024 (2016)
KH-RH S3-TC+ OVH-50	IGHV2-2	IGHD1-1	IGHJ4	ARDYGAYAMDY	27899442	Hayakawa et al. JEM 213:3007-3024 (2016)
KH-RH S3-TC+ OVH-51	IGHV2-2	IGHD1-1	IGHJ4	ARDYGAYAMDY	27899442	Hayakawa et al. JEM 213:3007-3024 (2016)
KH-RH S3-TC+ OVH-52	IGHV2-2	IGHD1-1	IGHJ1	ARNKFSNFITTVGGYFDY	27899442	Hayakawa et al. JEM 213:3007-3024 (2016)
KH-RH S3-TC+ OVH-53	IGHV2-2	IGHD2-4	IGHJ4	ARVYYDYGAMDY	27899442	Hayakawa et al. JEM 213:3007-3024 (2016)
KH-RH S3-TC+ OVH-54	IGHV5-17	IGHD2-4	IGHJ2	ARHDYYYFDY	27899442	Hayakawa et al. JEM 213:3007-3024 (2016)
KH-RH S3-TC+ OVH-55	IGHV5-17	IGHD2-10	IGHJ2	ARSYYGNFDY	27899442	Hayakawa et al. JEM 213:3007-3024 (2016)
KH-RH S3-TC+ OVH-56	IGHV5-17	IGHD3-3	IGHJ3	ARGATADRGFAY	27899442	Hayakawa et al. JEM 213:3007-3024 (2016)
KH-RH S3-TC+ OVH-57	IGHV5-17	IGHD3-3	IGHJ1	ARGTFDV	27899442	Hayakawa et al. JEM 213:3007-3024 (2016)
KH-RH S3-TC+ OVH-58	IGHV5-9-1	IGHD2-14	IGHJ2	TRDRYDDGGFDY	27899442	Hayakawa et al. JEM 213:3007-3024 (2016)
KH-RH S3-TC+ OVH-59	IGHV5-4	IGHD4-1	IGHJ1	ARGGRDFDV	27899442	Hayakawa et al. JEM 213:3007-3024 (2016)
KH-RH S3-TC+ OVH-60	IGHV5-9-1	IGHD1-1	IGHJ4	TREDSPYYYGSSYAMDY	27899442	Hayakawa et al. JEM 213:3007-3024 (2016)
KH-RH S3-TC+ OVH-61	IGHV6-4	IGHD2-7	IGHJ1	TRDYYGYDWYFDV	27899442	Hayakawa et al. JEM 213:3007-3024 (2016)
KH-RH S3-TC+ OVH-62	IGHV6-4	IGHD2-7	IGHJ1	TRDYYGYDWYFDV	27899442	Hayakawa et al. JEM 213:3007-3024 (2016)
KH-RH S3-TC+ OVH-63	IGHV14-2	N/A	IGHJ3	ARLAY	27899442	Hayakawa et al. JEM 213:3007-3024 (2016)
KH-RH S3-TC+ OVH-64	IGHV4-1	IGHD1-1	IGHJ2	ARPYYGSSYFDY	27899442	Hayakawa et al. JEM 213:3007-3024 (2016)
KH-RH S3-TC+ OVH-65	IGHV9-1	IGHD1-1	IGHJ2	AIYYVVDWYFDV	27899442	Hayakawa et al. JEM 213:3007-3024 (2016)
KH-RH S3-TC+ OVH-66	IGHV8-5	IGHD2-3	IGHJ2	AQIDFYYFDY	27899442	Hayakawa et al. JEM 213:3007-3024 (2016)
KH-RH S3-TC+ OVH-67	IGHV13-2	IGHD2-11	IGHJ4	SGGIYAMDY	27899442	Hayakawa et al. JEM 213:3007-3024 (2016)
KH-RH S2-Eu-Bcl1+12 St1-1	IGHV12-3			N/A	27899442	Hayakawa et al. JEM 213:3007-3024 (2016)
KH-RH S2-Eu-Bcl1+12 St2-1	IGHV1-55			N/A	27899442	Hayakawa et al. JEM 213:3007-3024 (2016)
KH-RH S2-Eu-Bcl1+12 St2-2	IGHV1-55			N/A	27899442	Hayakawa et al. JEM 213:3007-3024 (2016)
KH-RH S2-Eu-Bcl1+12 St3-1	IGHV2-9			N/A	27899442	Hayakawa et al. JEM 213:3007-3024 (2016)
KH-RH S2-Eu-Bcl1+12 St4-1	IGHV1-52			N/A	27899442	Hayakawa et al. JEM 213:3007-3024 (2016)
KH-RH S2-Eu-Bcl1+12 St4-2	IGHV1-52			N/A	27899442	Hayakawa et al. JEM 213:3007-3024 (2016)
KH-RH S2-Eu-Bcl1+12 St4-3	IGHV1-52			N/A	27899442	Hayakawa et al. JEM 213:3007-3024 (2016)
KH-RH S2-Eu-Bcl1+12 St4-4	IGHV1-52			N/A	27899442	Hayakawa et al. JEM 213:3007-3024 (2016)
KH-RH S2-Eu-Bcl1+12 St4-5	IGHV1-52			N/A	27899442	Hayakawa et al. JEM 213:3007-3024 (2016)
KH-RH S2-Eu-Bcl1+12 St5-1	IGHV11-2			N/A	27899442	Hayakawa et al. JEM 213:3007-3024 (2016)
KH-RH S2-Eu-Bcl1+50 OVH-1	IGHV1-9			N/A	27899442	Hayakawa et al. JEM 213:3007-3024 (2016)
KH-RH S2-Eu-Bcl1+50 OVH-2	IGHV1-9			N/A	27899442	Hayakawa et al. JEM 213:3007-3024 (2016)
KH-RH S2-Eu-Bcl1+50 OVH-3	IGHV1-9			N/A	27899442	Hayakawa et al. JEM 213:3007-3024 (2016)
KH-RH S2-Eu-Bcl1+50 OVH-4	IGHV1-9			N/A	27899442	Hayakawa et al. JEM 213:3007-3024 (2016)
KH-RH S2-Eu-Bcl1+50 OVH-5	IGHV1-82			N/A	27899442	Hayakawa et al. JEM 213:3007-3024 (2016)
KH-RH S2-Eu-Bcl1+50 OVH-6	IGHV62-2/71			N/A	27899442	Hayakawa et al. JEM 213:3007-3024 (2016)
KH-RH S2-Eu-Bcl1+50 OVH-7	IGHV1-76			N/A	27899442	Hayakawa et al. JEM 213:3007-3024 (2016)
KH-RH S2-Eu-Bcl1+50 OVH-8	IGHV1-64			N/A	27899442	Hayakawa et al. JEM 213:3007-3024 (2016)
KH-RH S2-Eu-Bcl1+50 OVH-9	IGHV1-64			N/A	27899442	Hayakawa et al. JEM 213:3007-3024 (2016)
KH-RH S2-Eu-Bcl1+50 OVH-10	IGHV1-64			N/A	27899442	Hayakawa et al. JEM 213:3007-3024 (2016)
KH-RH S2-Eu-Bcl1+50 OVH-11	IGHV1-55			N/A	27899442	Hayakawa et al. JEM 213:3007-3024 (2016)
KH-RH S2-Eu-Bcl1+50 OVH-12	IGHV1-52			N/A	27899442	Hayakawa et al. JEM 213:3007-3024 (2016)
KH-RH S2-Eu-Bcl1+50 OVH-13	IGHV1-52			N/A	27899442	Hayakawa et al. JEM 213:3007-3024 (2016)
KH-RH S2-Eu-Bcl1+50 OVH-14	IGHV1-52			N/A	27899442	Hayakawa et al. JEM 213:3007-3024 (2016)
KH-RH S2-Eu-Bcl1+50 OVH-15	IGHV1-52			N/A	27899442	Hayakawa et al. JEM 213:3007-3024 (2016)
KH-RH S2-Eu-Bcl1+50 OVH-16	IGHV1-52			N/A	27899442	Hayakawa et al. JEM 213:3007-3024 (2016)
KH-RH S2-Eu-Bcl1+50 OVH-17	IGHV1-80			N/A	27899442	Hayakawa et al. JEM 213:3007-3024 (2016)
KH-RH S2-Eu-Bcl1+50 OVH-18	IGHV12-3			N/A	27899442	Hayakawa et al. JEM 213:3007-3024 (2016)
KH-RH S2-Eu-Bcl1+50 OVH-19	IGHV14-4			N/A	27899442	Hayakawa et al. JEM 213:3007-3024 (2016)
KH-RH S2-Eu-Bcl1+50 OVH-20	IGHV14-4			N/A	27899442	Hayakawa et al. JEM 213:3007-3024 (2016)
KH-RH S2-Eu-Bcl1+50 OVH-21	IGHV14-2			N/A	27899442	Hayakawa et al. JEM 213:3007-3024 (2016)
KH-RH S2-Eu-Bcl1+50 OVH-22	IGHV3-2			N/A	27899442	Hayakawa et al. JEM 213:3007-3024 (2016)
KH-RH S2-Eu-Bcl1+50 OVH-23	IGHV7-3			N/A	27899442	Hayakawa et al. JEM 213:3007-3024 (2016)
UK-RDF-miR+/– #4	IGHV1-55	IGHD4-1	IGHJ2	AREETGPYYFDY	20060366	Klein et al. Cancer Cell 17:28-40 (2010)
UK-RDF-MDR–/– #313	IGHV3-2	IGHD2-1	IGHJ2	ARMELLWSPFDY	20060366	Klein et al. Cancer Cell 17:28-40 (2010)
UK-RDF-MDR+/– #290	IGHV2-6-4	IGHD1-1	IGHJ2	ARGVTTVVAPYFDY	20060366	Klein et al. Cancer Cell 17:28-40 (2010)
UK-RDF-miR–/– #59	IGHV5-16	IGHD4-1	IGHJ1	AREGEGDDGYYWYFDV	20060366	Klein et al. Cancer Cell 17:28-40 (2010)
UK-RDF-MDRfl/–Cd19-Cre #45	IGHV1-39	IGHD2-3	IGHJ2	ARGGIYDGYYDYFDY	20060366	Klein et al. Cancer Cell 17:28-40 (2010)
UK-RDF-miR–/– #189	IGHV4-1	IGHD2-4	IGHJ1	ARIYYDYYWYFDV	20060366	Klein et al. Cancer Cell 17:28-40 (2010)
UK-RDF-miRfl/–CD19-Cre #10	IGHV1-52	IGHD2-1	IGHJ1	AIYYGNYWYFDV	20060366	Klein et al. Cancer Cell 17:28-40 (2010)
UK-RDF-MDRfl/–CD19Cre #99	IGHV1-52	IGHD2-5	IGHJ1	ARYYSNYWYFDV	20060366	Klein et al. Cancer Cell 17:28-40 (2010)
UK-RDF-miRfl/–CD19Cre #234	IGHV1-52	IGHD2-5	IGHJ1	ARGEKYSNYWYFDV	20060366	Klein et al. Cancer Cell 17:28-40 (2010)
UK-RDF-MDR+/– #27	IGHV11-2	IGHD2-5	IGHJ1	MRYSNYWYFDV	20060366	Klein et al. Cancer Cell 17:28-40 (2010)
UK-RDF-MDR–/– #138	IGHV11-2	IGHD2-1	IGHJ1	MRYGNYWYFDV	20060366	Klein et al. Cancer Cell 17:28-40 (2010)
UK-RDF-MDRfl/–CD19-Cre #219	IGHV3-8	IGHD1-1	IGHJ1	YYGSSYWYFDV	20060366	Klein et al. Cancer Cell 17:28-40 (2010)

UK-RDF-MDR+/– #175-PB	IGHV12-3	IGHD2-10	IGHJ1	AGDRYGYWYFDV	20060366	Klein et al. Cancer Cell 17:28-40 (2010)
UK-RDF-miR+/+ #136	IGHV1-62-2/71	IGHD4-1	IGHJ1	ARHEEGNWDMYFDV	20060366	Klein et al. Cancer Cell 17:28-40 (2010)
UK-RDF-MDR–/– #58	IGHV11-2	IGHD4-1	IGHJ1	MSHNWDGWYFDV	20060366	Klein et al. Cancer Cell 17:28-40 (2010)
UK-RDF-MDRfl/–CD19-Cre #197	IGHV4-1	IGHD4-1	IGHJ1	ASPNWDWYFDV	20060366	Klein et al. Cancer Cell 17:28-40 (2010)
UK-RDF-MDR+/+CD19-Cre #212	IGHV2-2	IGHD4-1	IGHJ1	ARNWDWYFDV	20060366	Klein et al. Cancer Cell 17:28-40 (2010)
UK-RDF-MDR–/– #143	IGHV2-2	IGHD1-1	IGHJ4	ARKNYYGSSYEGYYAMDY	20060366	Klein et al. Cancer Cell 17:28-40 (2010)
UK-RDF-MDRfl/+CD19-Cre #211	IGHV3-2	IGHD1-1	IGHJ2	ARYYYGSSYYFDY	20060366	Klein et al. Cancer Cell 17:28-40 (2010)
UK-RDF-MDRfl/–CD19-Cre #246	IGHV6-3	IGHD1-1	IGHJ3	TEGYYGSSFAY	20060366	Klein et al. Cancer Cell 17:28-40 (2010)
UK-RDF-MDRfl/+CD19-Cre #215	IGHV1-9/63	IGHD4-1	IGHJ2	ARRTNWDEGDY	20060366	Klein et al. Cancer Cell 17:28-40 (2010)
ML-UK-CDR+/+ #51	IGHV6-6	IGHD1-1	IGHJ4	TRRGSSYYYAMDY	22174151	Lia et al. Blood 119:2981-2990 (2012)
ML-UK-CDR+/− #67	IGHV1-55	IGHD1-1	IGHJ1	ARRDYGSSYWYFDV	22174151	Lia et al. Blood 119:2981-2990 (2012)
ML-UK-CDR+/+ #114	IGHV11-2	IGHD2-1	IGHJ1	MRYGNYWYFDV	22174151	Lia et al. Blood 119:2981-2990 (2012)
ML-UK-CDR+/− #147	IGHV1-48	IGHD1-1	IGHJ4	ARCTTVVATKENAMDY	22174151	Lia et al. Blood 119:2981-2990 (2012)
ML-UK-CDR+/− #174	IGHV1-39	IGHD2-3	IGHJ1	ARGVYYDGYYPPFDV	22174151	Lia et al. Blood 119:2981-2990 (2012)
ML-UK-CDR+/− #179-J1	IGHV2-3	IGHD2-5	IGHJ1	AKEPYYSNYDYWYFDV	22174151	Lia et al. Blood 119:2981-2990 (2012)
ML-UK-CDR+/− #179-J4	IGHV3-5	IGHD4-1	IGHJ4	ARDPNWDYYYAMDY	22174151	Lia et al. Blood 119:2981-2990 (2012)
ML-UK-CDR+/− #181	IGHV11-2	IGHD2-1	IGHJ1	MRYGNYWYFDV	22174151	Lia et al. Blood 119:2981-2990 (2012)
ML-UK-CDRfl/flCD19-Cre #14	IGHV1-52	IGHD4-1	IGHJ2	AGNWDFDY	22174151	Lia et al. Blood 119:2981-2990 (2012)
ML-UK-CDRfl/flCD19-Cre #27	IGHV3-6	IGHD1-1	IGHJ3	ARDHYGSSYAWFAY	22174151	Lia et al. Blood 119:2981-2990 (2012)
ML-UK-CDRfl/+CD19-Cre #47	IGHV2-2	IGHD2-3	IGHJ2	ARIYDGYYYFDY	22174151	Lia et al. Blood 119:2981-2990 (2012)
ML-UK-CDRfl/flCD19-Cre #178	IGHV3-2	IGHD1-1	IGHJ2	ARDPYYYGSSLYYFDY	22174151	Lia et al. Blood 119:2981-2990 (2012)
ML-UK-CDRfl/flCD19-Cre #202	IGHV1-55	IGHD1-1	IGHJ1	ARIYYGSSYWYFDV	22174151	Lia et al. Blood 119:2981-2990 (2012)
ML-UK-CDR+/+CD19-Cre #30	IGHV1-82	N/A	IGHJ1	ARWDFDV	22174151	Lia et al. Blood 119:2981-2990 (2012)
ML-UK-CDRfl/−CD19-Cre #39	IGHV1-48	IGHD2-14	IGHJ4	ARSDYRSQYYYAMDY	22174151	Lia et al. Blood 119:2981-2990 (2012)
ML-UK-CDRfl/−CD19-Cre #184-J1	IGHV5-6-2	IGHD1-1	IGHJ1	ASYYGYWYFDV	22174151	Lia et al. Blood 119:2981-2990 (2012)
ML-UK-CDRfl/−CD19-Cre #184-J4	IGHV10-3	IGHD2-3	IGHJ4	VRDDYDAMDY	22174151	Lia et al. Blood 119:2981-2990 (2012)
ML-UK-CDRfl/−CD19-Cre #224	IGHV5-9-4	IGHD2-3	IGHJ2	ARDDGYYYFDY	22174151	Lia et al. Blood 119:2981-2990 (2012)
ML-UK-CDR+/−CD19-Cre #225	IGHV11-2	IGHD2-5	IGHJ1	MRYSNYWYFDV	22174151	Lia et al. Blood 119:2981-2990 (2012)
ML-UK-CDRfl/−CD19-Cre #304-J1	IGHV1-42	IGHD2-4	IGHJ1	ASHDYDWYFDV	22174151	Lia et al. Blood 119:2981-2990 (2012)
ML-UK-CDRfl/−CD19-Cre #304-J2	IGHV1-64	IGHD2-5	IGHJ2	ARDYSNYDY	22174151	Lia et al. Blood 119:2981-2990 (2012)
ML-UK-CDRfl/−CD19-Cre #308-J1	IGHV1-52	IGHD5-5	IGHJ1	ARPSLPYWYFDV	22174151	Lia et al. Blood 119:2981-2990 (2012)
ML-UK-CDRfl/−CD19-Cre #308-J2	IGHV3-2	IGHD2-4	IGHJ2	ARGYDYDDYFDY	22174151	Lia et al. Blood 119:2981-2990 (2012)
ML-UK-CDR+/−CD19-Cre #328	IGHV1-55	IGHD6-4	IGHJ4	AREEAGYYAMDY	22174151	Lia et al. Blood 119:2981-2990 (2012)
ML-UK-CDRfl/−CD19-Cre #330	IGHV1-14	IGHD1-2	IGHJ3	ARERLDYGYEAWFAY	22174151	Lia et al. Blood 119:2981-2990 (2012)
tB-RH-T1-T1	IGHV11-2	IGHD3-1	IGHJ1	MRYSNYWYFDV	19332766	ter Brugge et al. Blood 114:119-127 (2009)
tB-RH-T1-T5	IGHV11-2	IGHD3-1	IGHJ1	MRYSNYWYFDV	19332766	ter Brugge et al. Blood 114:119-127 (2009)
tB-RH-T1-T20	IGHV11-2	IGHD1-1	IGHJ1	MRYGSSYWYFDV	19332766	ter Brugge et al. Blood 114:119-127 (2009)
tB-RH-T1-T23	IGHV11-2	IGHD2-1	IGHJ1	MRYGNYWYFDV	19332766	ter Brugge et al. Blood 114:119-127 (2009)
tB-RH-T1-T4	IGHV11-2	IGHD1-1	IGHJ1	MRYGSSYWYFDV	19332766	ter Brugge et al. Blood 114:119-127 (2009)
tB-RH-T1-T9	IGHV1-18	IGHD1-1	IGHJ3	ARRDYGSSYVGWFAY	19332766	ter Brugge et al. Blood 114:119-127 (2009)
tB-RH-T1-T16	IGHV1-55	N/A	IGHJ2	ARGFDY	19332766	ter Brugge et al. Blood 114:119-127 (2009)
tB-RH-T1-T3	IGHV1-58	IGHD2-4	IGHJ3	ARERVYDYDLAWFAY	19332766	ter Brugge et al. Blood 114:119-127 (2009)
tB-RH-T1-T23	IGHV1-69	IGHD1-2	IGHJ2	ARYDYYYYYC	19332766	ter Brugge et al. Blood 114:119-127 (2009)
tB-RH-T1-T32	IGHV1-74	IGHD3-3	IGHJ4	ARDDPLGYAMDY	19332766	ter Brugge et al. Blood 114:119-127 (2009)
RB-CMC-T2-9ma	IGHV2-2	IGHD2-9	IGHJ4	ARNGYDYAMDY	12011454	Bichi et al. PNAS 99:6955-6960 (2002)
RB-CMC-T2-9mb	IGHV2-2	IGHD1-1	IGHJ4	ARTVVALYAMDY	12011454	Bichi et al. PNAS 99:6955-6960 (2002)
RB-CMC-T2-8m	IGHV14-1	IGHD1-1	IGHJ1	TPHYGSSLWYFDV	12011454	Bichi et al. PNAS 99:6955-6960 (2002)
