# Supplementary material for: Fc Receptor-Like 6 (FCRL6) Discloses Progenitor B Cell Heterogeneity That Correlates With Pre-BCR Dependent and Independent Pathways of Natural Antibody Selection
Source: Front Immunol. 2020 Feb 14;11:82. doi: 10.3389/fimmu.2020.00082 (PMC7033751; doi:10.3389/fimmu.2020.00082)
Supplement: Supplementary file 5 [file Data_Sheet_1.pdf]

Key resources table

Reagent type (species) Reagent type (species) Or resource	Designation	Source or reference	Identifiers	Additional information
Antibody	BV421 rat anti-mouse CD19 (clone 6D5)	BioLegend	115537	(1:100)
Antibody	APC/Cy7 rat anti-mouse CD19 (clone 6D5)	BioLegend	115530	(1:50)
Antibody	PE rat anti-mouse/human B220 (clone RA3-6B2)	BD	553090	(1:200)
Antibody	PE/Cy7 rat anti-mouse/human B220 (clone RA3-6B2)	BioLegend	103222	(1:100)
Antibody	BV510 rat anti-mouse/human B220 (clone RA3-6B2)	BioLegend	103248	(1:25)
Antibody	FITC rat anti-mouse CD43 (clone S7)	BD	553270	(1:50)
Antibody	PE rat anti-mouse CD43 (clone S7)	BD	561857	(1:100)
Antibody	PE/Cy7 rat anti-mouse CD93 (clone AA4.1)	BioLegend	136505	(1:200)
Antibody	FITC rat anti-mouse/human CD11b (clone M1/70)	eBioscience	11-0112-81	(1:100)
Antibody	PerCP/Cy5.5 rat anti-mouse/human CD11b (clone M1/70)	BioLegend	101228	(1:100)
Antibody	PE hamster anti-mouse CD11c (clone HL3)	BD	561044	(1:200)
Antibody	PerCP/Cy5.5 rat anti-mouse TER-119 (clone TER-119)	BioLegend	116228	(1:100)
Antibody	PE rat anti-mouse Gr-1 (clone RB6-8C5)	eBioscience	12-5931-81	(1:400)
Antibody	PerCP/Cy5.5 rat anti-mouse Gr-1 (clone RB6-8C5)	BioLegend	108428	(1:100)
Antibody	FITC hamster anti-mouse CD3 (clone 145-2C11)	eBioscience	11-0031-82	(1:50)
Antibody	biotin hamster anti-mouse CD3 (clone 145-2C11)	eBioscience	13-0031-82	(1:400)
Antibody	PerCP/Cy5.5 rat anti-mouse CD3 (clone 17A2)	BioLegend	100218	(1:50)
Antibody	PE rat anti-mouse CD5 (clone 53-7.3)	BioLegend	100607	(1:200)

Antibody	APC rat anti-mouse CD21 (clone 7E9)	BioLegend	123412	(1:400)
Antibody	PE/Cy7 rat anti-mouse CD23 (clone B3B4)	BioLegend	101614	(1:200)
Antibody	PE rat anti-mouse CD24 (clone 30-F1)	BioLegend	138503	(1:800)
Antibody	AF647 hamster anti-mouse/rat CD29 (clone HMß1-1)	BioLegend	102213	(1:100)
Antibody	PE rat anti-mouse CD49d (clone R1-2)	BioLegend	103607	(1:100)
Antibody	PE hamster anti-mouse CD69 (clone H1.2F3)	BD	561932	(1:100)
Antibody	PE rat anti-mouse CD117 (clone 2B8)	BioLegend	105807	(1:200)
Antibody	PE rat anti-mouse CD127 (clone A7R34)	eBioscience	12-1271-81	(1:50)
Antibody	PE rat anti-mouse CD138 (clone 281-2)	BD	553714	(1:50)
Antibody	APC rat anti-mouse CD150 (clone TC15-12F12.2)	BioLegend	115909	(1:100)
Antibody	PE rat anti-mouse CD179a (clone R3)	BioLegend	143603	(1:200)
Antibody	biotin rat anti-mouse CD179b (clone LM34)	BD	551865	(1:100)
Antibody	biotin rat anti-mouse BP-1 (clone 6C3)	BioLegend	108303	(1:100)
Antibody	PE rat anti-mouse I-A/I-E (clone M5/114.15.2)	eBioscience	12-5321-81	(1:200)
Antibody	PE rat anti-mouse LFA-1 (clone H155-78)	BioLegend	141005	(1:100)
Antibody	PE rat anti-mouse CXCR5 (clone SPRCL5)	eBioscience	12-7185-80	(1:30)
Antibody	APC rat anti-mouse CCR7 (clone 4B12)	BioLegend	120107	(1:30)
Antibody	PE goat anti-mouse TSLPR (polyclonal)	R&D	FAB5461P	(1:10)
Antibody	Cy5 F(ab')2 goat anti-mouse IgM (polyclonal)	Jackson IR	115-176-075	(1:2000)
Antibody	FITC rat anti-mouse IgM (clone II/41)	eBioscience	11-5890-82	(1:100)
Antibody	FITC goat anti-mouse IgM (polyclonal)	Southern Biotech	1020-02	(1:400)
Antibody	PerCP/Cy5.5 rat anti-mouse IgM (clone RMM-1)	BioLegend	406512	(1:50)
Antibody	PE goat anti-mouse IgG+M (polyclonal)	Southern Biotech	3010-09	(1:200)

Antibody	PE rat anti-mouse kappa chain (clone 187.1)	BD	562021	(1:400)
Antibody	FITC mouse anti-BrdU (clone 3D4)	BioLegend	364103	(1:10)
Antibody	BV421 rat anti-mouse Pre-BCR (clone SL156)	BD	744898	(1:140)
Antibody	Pacific Blue rat anti-mouse Ki-67 (clone 16A8)	BioLegend	652421	(1:40)
Antibody	PE mouse anti-mouse Aiolos (clone 8B2)	BioLegend	653203	(1:50)
Antibody	PE mouse anti-mouse Ikaros (clone 2A9/Ikaros)	BioLegend	653303	(1:200)
Antibody	PE anti-mouse/human/rat NFAT2 (clone 7A6)	BioLegend	649605	(1:200)
Antibody	PE Rabbit anti-mouse/human c-Myc (clone D84C12)	Cell signaling	14819S	(1:300)
Antibody	A647 mouse anti-mouse/human/rat ERK1/2 (pT202/pY204) (clone 20A)	BD	612593	(1:10)
Antibody	A647 mouse anti-mouse/human/rat/sheep Stat5 (pY694) (clone 47/Stat5(pY694))	BD	612599	(1:10)
Antibody	Unlabeled rat anti-mouse CD16/32 (clone 93)	BioLegend	101302	(1:50)
Antibody	biotin rat anti-mouse FCRL6 (clone M6-3C1)	This paper	in-house	(1:300)
Antibody	AF647 rat anti-mouse FCRL6 (clone M6-3C1)	This paper	in-house	(1:50)
Antibody	biotin rat anti-mouse FCRL6 (clone M6-1C3)	This paper	in-house	(1:400)
Antibody	AF647 rat anti-mouse FCRL6 (clone M6-1C3)	This paper	in-house	(1:400)
Antibody	AF647 rat anti-mouse VH7 (clone TC68)	PMID: 6209566	J. Kearney	(1:10000)
Antibody	biotin rat anti-mouse VH11 (clone 3H7)	PMID: 17889506	K. Hayakawa	(1:14000)
Antibody	AF647 rat anti-mouse VH11 (clone 3H7)	PMID: 17889506	K. Hayakawa	(1:400)
Antibody	biotin rat anti-mouse VH12 (clone CH27Id.5C5)	PMID: 8163938	K. Rajewsky	(1:12000)
Other	FITC Annexin V	BioLegend	640906	(1:300)
Other	BV510 Streptavidin	BioLegend	405233	(1:100)
Other	BV421 Streptavidin	BioLegend	405226	(1:100)

Other	BV570 Streptavidin	BioLegend	405227	(1:100)
Other	BV650 Streptavidin	BioLegend	405231	(1:100)
Other	PE Streptavidin	BD	554061	(1:400)
Chemical compound, drug	Propidium Idodide	Sigma	P4864	(1:6000)
Chemical compound, drug	5-Bromo-2′-deoxyuridine	Sigma	B5002	1 mg/ injection
Chemical compound, drug	DNase I	Sigma	D5025	0.3 mg/ml
Peptide, recombinant protein	mouse TSLP	eBioscience	14-8498-62	10 ng/ml
Peptide, recombinant protein	Murine IL-3	Peprotech	213-13	5 ng/ml
Peptide, recombinant protein	Murine IL-6	Peprotech	216-16	10 ng/ml
Peptide, recombinant protein	Murine SCF	Peprotech	250-03	10 ng/ml
Peptide, recombinant protein	Murine Flt3L	Peprotech	250-31L	10 ng/ml
